# Oxidation, Coordination, and Nickel‐Mediated Deconstruction of a Highly Electron‐Rich Diboron Analogue of 1,3,5‐Hexatriene

**DOI:** 10.1002/anie.202006131

**Published:** 2020-07-01

**Authors:** Alexander Hermann, Felipe Fantuzzi, Merle Arrowsmith, Theresa Zorn, Ivo Krummenacher, Benedikt Ritschel, Krzysztof Radacki, Bernd Engels, Holger Braunschweig

**Affiliations:** ^1^ Institute for Inorganic Chemistry & the Institute for Sustainable, Chemistry & Catalysis with Boron Julius-Maximilians-University Würzburg Am Hubland 97074 Würzburg Germany; ^2^ Institute for Physical and Theoretical Chemistry Julius-Maximilians-University Würzburg Emil-Fischer-Stra*ß*e 42 97074 Würzburg Germany

**Keywords:** carbenes, conjugation, density-functional calculations, rearrangements, structure elucidation

## Abstract

The reductive coupling of an N‐heterocyclic carbene (NHC) stabilized (dibromo)vinylborane yields a 1,2‐divinyldiborene, which, although isoelectronic to a 1,3,5‐triene, displays no extended π conjugation because of twisting of the C_2_B_2_C_2_ chain. While this divinyldiborene coordinates to copper(I) and platinum(0) in an η^2^‐B_2_ and η^4^‐C_2_B_2_ fashion, respectively, it undergoes a complex rearrangement to an η^4^‐1,3‐diborete upon complexation with nickel(0).

## Graphical abstract

**A complex twist**: A highly electron‐richN‐heterocyclic carbene stabilized 1,2‐divinyldiborene, which displays no delocalized π conjugation because of twisting of the C_2_B_2_C_2_ chain, coordinates to Cu^I^ and Pt^0^ in an η^2^‐B_2_ and η^4^‐C_2_B_2_ mode, respectively, while undergoing a complex rearrangement to an η^4^‐1,3‐diborete upon complexation with Ni^0^.

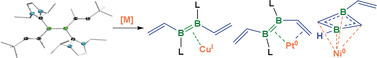

## Introduction

**Figure 1:**
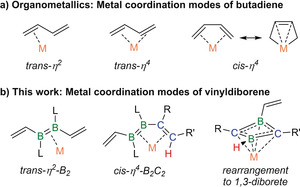
Metal coordination to dienes and divinyldiborenes.

Linear conjugated alkenes owe their intrinsic stability to the delocalization of their π electrons. Found in many natural products and biologically relevant compounds,^1^ they are also important building blocks in organic synthesis and materials chemistry. Conjugated trienes and higher oligoenes have attracted interest because of their photophysical properties, which enable applications in nonlinear optics and optical sensing.^2^ In an industrial setting, conjugated dienes (e.g. butadiene, isoprene) are mainly used as monomers for the Ziegler–Natta synthesis of synthetic rubbers.^3^ In organic chemistry, they are principally employed in 1,4‐addition^4^ and Diels–Alder reactions,^5^ as well as numerous other transformations.^6^ Many of these reactions are metal‐catalyzed and involve transition‐metal (TM) 1,3‐diene complexes as key reaction intermediates. Solution and solid‐state analyses of such complexes show that the diene ligand can be found either in the *cis* or *trans* conformation and switch between the *η* ^2^ and η^4^ coordination modes (Figure 1 a),^7^ which may determine its subsequent reactivity with incoming substrates.

The substitution of one or more carbon atoms with more electronegative heteroatoms (e.g. N, O) has long been exploited to generate polar conjugated systems, which are employed in numerous organic reactions (e.g. Michael additions^8^ and hetero‐Diels–Alder reactions).^9^ In contrast, however, the chemistry of conjugated heterodienes or heterotrienes in which one or more carbon atoms have been substituted with a more electropositive element is virtually unexplored.

One of the focuses of our research is on the synthesis, reactivity, and metal coordination of compounds displaying boron–element^10^ and boron–boron multiple bonds.^11^, ^12^, ^13^, ^14^, ^15^ Among the latter, doubly base‐stabilized diborenes, which are formally isoelectronic and isostructural to alkenes, have been relatively well studied since their first isolation by Robinson in 2007.^16^ Unlike most alkenes, diborenes undergo 1,2‐addition and [2+2] cycloaddition reactions without the need for a catalyst, owing to their high‐lying HOMO and relatively low‐lying LUMO.^11^ While the B=B bond coordinates to coinage metals in an η^2^ fashion reminiscent of metal–olefin π complexes,^12^ it is also sufficiently electron‐rich to bind to Zn^II^, Cd^II^,^13^ and even Mg^II^ centers,^17^ which do not tend to form stable π‐olefin complexes because of their limited capacity for π backdonation. DFT calculations have shown that the B_2_–M interaction in these complexes is mostly electrostatic in nature (ca. 60–70 %), with the electron‐rich B=B bond donating into empty orbitals of the metal center, and no or little π backbonding from the metal to the diborene unit.^12^, ^13^, ^17^ The only reactivity reported to date for such TM‐diborene complexes is that of a PMe_3_‐stabilized bis(9‐anthryl)diborene, which upon complexation to copper triflate undergoes an intramolecular hydroarylation.12b

Interested in expanding the coordination chemistry and reactivity of diborenes to conjugated systems isoelectronic to 1,3,5‐trienes, we set out to synthesize doubly base‐stabilized 1,2‐divinyl‐substituted diborenes. In this contribution we describe the synthesis of a 3,4‐dibora‐1,3,5‐triene, explore its electronic configuration and its oxidation chemistry, and present its various coordination modes to Cu^I^ and Pt^0^ metal centers (Figure 1 b), as well as its Ni^0^‐mediated rearrangement into a 1‐vinyl‐1,3‐diborete.

## Results and Discussion

**Figure 2:**
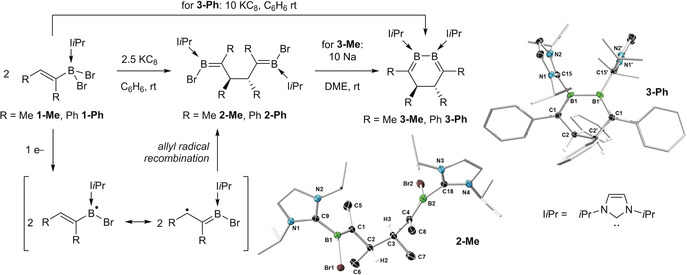
Reduction of **1‐Me** and **1‐Ph** with postulated radical mechanism and crystallographically derived molecular structures of **2‐Me** and **3‐Ph**. Thermal ellipsoids set at 50 % probability.^39^ Thermal ellipsoids of ligand periphery and hydrogen atoms omitted for clarity.

The NHC‐stabilized dibromovinylboranes (I*i*Pr)BBr_2_(C(R)=CHR′) (I*i*Pr=1,3‐di*iso*propylimidazol‐2‐ylidene; R=R′=Me **1‐Me**, Ph **1‐Ph**; R=Me, R′=tBu **1‐*t*Bu**) were synthesized by hydroboration of the corresponding RC≡CR′ alkyne precursors with HBCat (Cat=catecholate),^18^ followed by adduct formation with I*i*Pr and bromination with BBr_3_.^19^ The reduction of **1‐Me** and **1‐Ph** with 2.5 equivalents KC_8_ in benzene at room temperature resulted in relatively unselective reactions from which the only isolable crystalline products were the bis(I*i*Pr)‐stabilized 1,4‐bis(bromoboraneylidene)butanes **2‐Me** [δ(^11^B)=12.5 ppm, broad] and **2‐Ph** [δ(^11^B)=18.4 ppm, broad], resulting from radical C−C coupling of two vinylborane units at the *β* position. **1‐Me** and **1‐Ph** could be further reduced with 10 equivalents KC_8_, albeit unselectively,^20^ to the corresponding doubly I*i*Pr‐stabilized 4,5‐dihydro‐1,2‐diborinines **3‐Me** [δ(^11^B)=19.9 ppm, broad] and **3‐Ph** [δ(^11^B)=27.5 ppm, broad; Figure 2].^21^

While repeated attempts at isolating analytically pure samples of **2‐R** and **3‐R** (R=Me, Ph) failed because of co‐crystallization with other unidentified reaction products, the solid‐state structures of **2‐Ph** (see Figure S52 in the Supporting Information), **2‐Me**, and **3‐Ph** (Figure 2) were unequivocally determined by X‐ray diffraction analyses. These confirmed the presence of the B=C bonds [**2‐Me** B1‐C1 1.423(2), B2‐C4 1.426(2); **2‐Ph** B1‐C1 1.439(3), B2‐C4 1.436(3); **3‐Ph** B1‐C1 1.468(4) Å],^22^ the newly formed C−C single bonds [**2‐Me** C2‐C3 1.551(2); **2‐Ph** C2‐C3 1.558(2); **3‐Ph** C2‐C2′ 1.530(4) Å], and the endocyclic B−B bond in **3‐Ph** [1.694(5) Å]. Unfortunately, all attempts to synthesize these compounds more selectively failed.

**Scheme 1:**
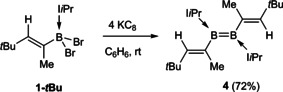
Reduction of **1‐*t*Bu** to **4**.

In contrast, the reduction of **1‐*t*Bu** with 4 equivalents KC_8_ in benzene at room temperature led to the selective formation of the red‐colored divinyldiborene **4**, which was isolated in 72 % yield as a brown solid (Scheme 1). The presence of the sterically demanding β‐*tert*‐butyl substituent prevents the recombination of the intermediate β‐carbon‐centered radical, favoring instead further reduction of the boron center and boron–boron bond formation. The diborene **4** presents a broad ^11^B NMR resonance at *δ*=25.1 ppm, in the range typical for NHC‐stabilized diborenes.^11^ The ^1^H NMR spectrum displays a characteristic 2H quartet at *δ*=4.80 ppm (^4^ *J*=1.2 Hz) for the vinylic protons coupling to the *tert*‐butyl protons and correlating by HSQC to a ^13^C{^1^H} NMR resonance at *δ*=132.4 ppm.

**Figure 3:**
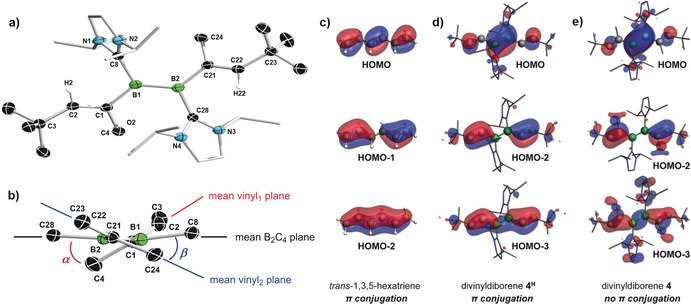
a) Crystallographically derived molecular structure of **4**.^39^ Thermal ellipsoids at 50 % probability. Thermal ellipsoids of ligand periphery and hydrogen atoms omitted for clarity. Selected bond lengths [Å] and angles [°]: B1–B2 1.601(2), B1–C1 1.600(2), B1–C8 1.581(2), B2–C21 1.589(2), B2–C28 1.584(2), C1–C2 1.354(2), C21–C22 1.354(2), Σ∡_B1_ 359.57(14), Σ∡_B2_ 359.89(14), torsion (C1,B1,B2,C21) 177.06(16), (C8,B1,B2,C28) 164.82(14). b) Truncated view of **4** along the B_2_ plane. Angles between mean planes: *α*=37.9°, *β*=30.7°. Plots of the π bonding frontier molecular orbitals of 1,3,5‐hexatriene (c), **4^H^** (d), an analogue of **4** in which the methyl groups at the *α*‐vinyl positions have been truncated, and **4** (e) at the B3LYP/6–311G** level of theory.

The solid‐state structure of **4** is shown in Figure 3 a. The B−B bond length of 1.601(2) Å lies in the upper range of B=B bonds^11^ and is similar to that found in doubly I*i*Pr‐stabilized di(2‐thienyl)^23^ or ferrocenediyl‐bridged diborenes,^24^ for example. The diborene core is slightly distorted from planarity, with (C1,B1,B2,C21) and (C8,B1,B2,C28) torsion angles of 177.06(16) and 164.82(14)°, respectively. The vinyl groups display localized B−C single [1.581(2) and 1.589(2) Å] and C=C double bonds [1.354(2) and 1.354(2) Å], and a *Z* configuration of the alkyl substituents. Figure 3 b shows that the vinyl units are not coplanar with the mean plane of the diborene, and are rotated by *α*=37.9° and *β*=30.7°. This conformation may arise from steric repulsion between the NHC *i*Pr and the vinyl α‐methyl groups. As a result, there is no π conjugation possible between the C=C and B=B bonds. This lack of π conjugation is supported by DFT calculations at the B3LYP/6–311G** level,^25^ which show that the π‐bonding molecular orbitals of **4** are largely localized: the HOMO on the B=B bond, the HOMO‐2 on the two C=C bonds and the HOMO‐3 slightly delocalized over each of the C_2_B fragments (Figure 3 e). This localization contrasts with the extensive π delocalization observed in the related and entirely planar 1,3,5‐hexatriene molecule (Figure 3 c). It is noteworthy that calculations on an analogue of **4** in which the methyl groups at the α‐vinyl positions have been replaced by protons, the compound **4^H^**, yield a quasi‐planar C_2_B_2_C_2_ core with significantly shortened B−C bonds (1.566 Å), which displays more extensive π conjugation than **4** (Figure 3 d). Consequently, the lack of planarity and π conjugation in **4** can be ascribed mainly to the sterics at the α‐vinyl positions. The UV‐vis spectrum of **4** displays two absorption maxima at 453 and 573 nm, which account for the brown color of the compound. TDDFT calculations at the same level of theory provide a good match for the absorption maxima (439 and 582 nm) and show that the absorption at 573 nm results from a HOMO→LUMO transition (89 %), while that at 453 nm is related to a HOMO→LUMO+1 transition (89 %).

**Figure 4:**
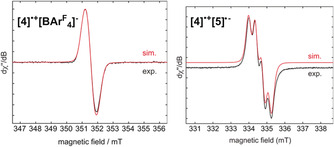
Experimental (black) and simulated (red) EPR spectra of **[4]^.+^[BAr^F^**_**4**_ **]^−^** in *o*‐difluorobenzene (left) and **[4]^.+^[5]^.−^** in hexanes (right) at room temperature. Simulated parameters are as follows: **[4]^.+^[BAr^F^**_**4**_ **]^−^** *g*_iso_=2.0023, *a*(B)=ca. 1.7 G (peak‐to‐peak width=7 G), **[4]^.+^[5]^.−^** *g*_iso_=2.0025, *a*(B)=3.5 G (**[5]^.−^**); *g*_iso_=2.0023, *a*(B)=<1 G (**[4]^.+^**).

**Scheme 2:**
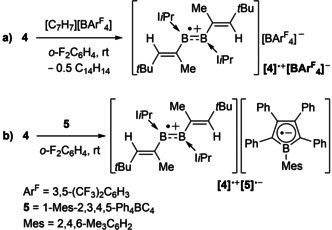
One‐electron oxidation of **4**.

While **4** was indefinitely stable under inert conditions in the solid state and in benzene solution up to 80 °C, it decomposed entirely within minutes in polar solvents (THF, *o*‐difluorobenzene), thereby preventing the acquisition of cyclic voltammetry data. Its reducing power was, however, confirmed by one‐electron oxidation with [C_7_H_7_][BAr^F^_4_] (Ar^F^=3,5‐trifluoromethylphenyl), which yielded the red‐purple radical cation **[4]^.+^[BAr^F^**_**4**_ **]^−^** (Scheme 2 a). Like other [(NHC)_2_B_2_R_2_]^⋅+^ radical cations with [BAr^F^_4_]^−^ as the counteranion,^26^ the EPR spectrum of **[4]^.+^[BAr^F^**_**4**_ **]^−^** in *o*‐difluorobenzene showed a broad signal, for which a simulation provided a hyperfine coupling parameter of *a*(B)=ca. 1.7 G (Figure 4, left). We have shown that the doubly I*i*Pr‐stabilized 1,2‐di*iso*propyldiborene is sufficiently reducing to react with 1‐mesityl‐2,3,4,5‐tetraphenylborole (*E*_red_=−1.69 V, mesityl=2,4,6‐Me_3_C_6_H_2_)^27^ to form the radical cation/radical anion pair [(I*i*Pr)_2_B_2_ *i*Pr_2_]^⋅+^[MesBC_4_Ph_4_]^.−^.26b Similarly, **4** reacted with the borole **5** to yield the radical cation/radical anion pair **[4]^.+^[5]^.−^** (Scheme 2 b), which displays a broad EPR signal consisting of the overlap of the radical cation and radical anion resonances (Figure 4, right). Given the success of this single‐electron transfer reaction, we can conclude that the redox potential of **4** must be lower than that of **5**, that is, −1.69 V. The lowest redox potential of a diborene measured to date remains that of [(I*i*Pr)_2_B_2_ *i*Pr_2_] at −1.95 V.26b Since the oxidation potential of conjugated alkenes to radical cations occurs generally above 1 V,^28^ we can conclude that the oxidation of **4** occurs exclusively at the diboron core and not at the vinyl moieties.

**Scheme 3:**
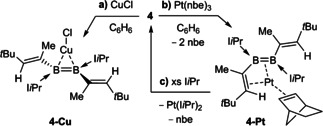
Synthesis of the π‐diborene copper complex **4‐Cu** and π‐(1,2‐diborabutadiene) Pt^0^ complex **4‐Pt** (nbe=norbornene).

Like other diborenes, **4** formed a π‐diborene complex with CuCl, **4‐Cu** (Scheme 3 a).12a, 12b, 12c The bright‐yellow compound displays a broad ^11^B NMR resonance at *δ*=19.5 ppm, slightly upfield with respect to **4** [δ(^11^B)=25.1 ppm] in accordance with other known coinage metal diborene complexes.^12^ Similarly, the ^1^H NMR quartet of the vinylic protons (2 H) is upfield‐shifted from *δ*=4.80 ppm in **4** to *δ*=4.29 ppm in **4‐Cu**. The solid‐state structure of **4‐Cu** (Figure 6) is similar to those reported for other π‐diborene copper complexes,12a, 12b, 12c with a slight elongation of the B−B bond [1.627(4) Å] compared to that in **4** [1.601(2) Å] and increased distortion of the diborene core away from planarity [Σ∡_B1_ 357.8(3), Σ∡_B2_ 357.7(3), torsion angles (C1,B1,B2,C21) −166.6(3), (C8,B1,B2,C28) 156.1(3)°].

**Figure 5:**
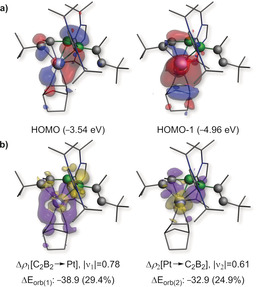
a) Plot of the HOMO and HOMO‐1 of **4‐Pt** at the B3LYP/TZV2P level of theory. b) Plot of deformation densities (Δ*ρ*_k_), at the same level of theory, of the orbital interactions of the C_2_B_2_ fragment π‐donating to the Pt^0^ center (left) and the Pt center π‐backdonating to the C_2_B_2_ fragment (right). The |ν_k_| values correspond to the eigenvalues of the complementary eigenfunctions (*ψ*_−k_, *ψ*_k_) in the NOCV representation, while Δ*E*_orb(k)_ is the *k* ^th^ orbital interaction energy (kcal mol^−1^), with the percentage contribution to the total orbital interaction energy (Δ*E*_orb_) shown within parentheses. The electron density flows from yellow to purple.

In contrast, the reaction of Pt(nbe)_3_ (nbe=norbornene) with **4** yielded the pink‐colored 1,2‐diborabutadiene complex **4‐Pt** (Scheme 3 b). The solid‐state structure of **4‐Pt** shows that the divinyldiborene has displaced two of the nbe ligands and coordinates to platinum through π interactions with both the B=B [B1‐Pt1 2.248(4), B2‐Pt1 2.343(4) Å] and one of the C=C bonds [C1‐Pt1 2.227(3), C2‐Pt1 2.236(3) Å; Figure 5], which is rotated in the direction of the metal center to give a *cis*‐η^4^ configuration. This cis‐η^4^ binding mode contrasts with the bonding of Pt^0^ to conjugated olefins, which is usually limited to η^2^,^29^ and highlights the much better π‐donor and π‐acceptor capacities of the B=B bond relative to those of the C=C bond.^11^ **4‐Pt** is the first complex of a doubly base‐stabilized diborene with a transition metal outside groups 11 and 12, the other two known platinum diborene complexes being of the base‐free diborene DurB=BDur (Dur=2,3,5,6‐Me_4_C_6_H).^14^ The B1−B2 [1.637(5) Å] and C1−C2 bonds [1.418(5) Å] are significantly longer than those of **4** [B1‐B2 1.601(2), C1‐C2 1.354(2) Å], while the B1−C1 bond [1.572(5) Å] is shorter [**4** 1.600(2) Å], which suggests some amount of π delocalization over the platinum‐bound B2‐B1‐C1‐C2 unit.

**Figure 6:**
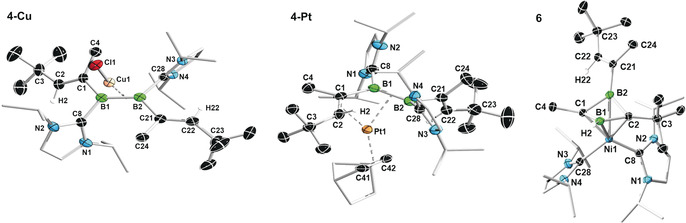
Crystallographically derived molecular structures of **4‐Cu** (the non‐disordered one of the two distinct molecules present in the asymmetric unit), **4‐Pt**, and **6**.^39^ Thermal ellipsoids set at 50 % probability. Thermal ellipsoids of ligand periphery and hydrogen atoms omitted for clarity. Selected bond lengths [Å] and angles [°] for **4‐Cu**: B1–B2 1.627(4), B1–Cu1 2.148(3), B2–Cu1 2.144(3), Cu1–Cl1 2.1615(10), B1–C1 1.594(4), B1–C8 1.595(4), B2–C21 1.592(4), B2–C28 1.600(4), C1–C2 1.344(4), C21–C22 1.345(4), Σ∡_B1_ 357.8(3), Σ∡_B2_ 357.7(3), torsion (C1,B1,B2,C21) −166.6(3), (C8,B1,B2,C28) 156.1(3), *α*=29.4, *β*=27.7, angle between mean B_2_C_4_ and B_2_CuCl planes=89.3; **4‐Pt**: B1–B2 1.637(5), B1–Pt1 2.248(4), B2–Pt1 2.343(4), C1–Pt1 2.227(3), C2–Pt1 2.236(3), Pt1–C41 2.116(3), Pt1–C42 2.076(3), B1–C1 1.572(5), B1–C8 1.602(5), B2–C21 1.621(5), B2–C28 1.630(5), C1–C2 1.418(5), C21–C22 1.348(5); B2‐B1‐C1 123.3(3), B1‐C1‐C2 122.0(3), Σ∡_B1_ 359.4(3) Σ∡_B2_ 156.1(3), *α*=16.2, *β*=72.0; **6**: B1–B2 1.890(2), B1–C1 1.5584(19), B1–C2 1.5430(19), B2–C1 1.5584(19), B2–C2 1.5506(19), B1–H2 1.106(17), B1–Ni1 2.2491(15), B2–Ni1 2.2466(14), C1–Ni1 1.9710(12), C2–Ni1 2.0000(13), Ni1–C8 1.9194(13), Ni1–C28 1.9111(14), C21–C22 1.3396(19).

DFT calculations show that the HOMO of **4‐Pt** is a π orbital mostly localized on the B=B bond donating into an empty *d* orbital at the platinum center, with only a small contribution of the C=C bond and a node at the B1−C1 bond (Figure 5 a). The HOMO‐1 consists mainly of the π orbital of the nbe ligand donating to the Pt center, with a small π‐bonding component localized on the B1−C1 bond, as already suggested by its shortened bond length in the solid‐state structure (Figure 6). The nature of the Pt–C_2_B_2_ bonding was further analyzed by energy decomposition analysis combined with the natural orbitals for chemical valence theory (EDA‐NOCV).^30^ The results suggest that the bonding in **4‐Pt** is dominated by electrostatics (65.0 %), with non‐negligible orbital interaction contributions (35.0 %). These arise from a combination of equal amounts of the C_2_B_2_ π‐symmetrized fragment orbital (SFO), mostly centered on the B=B bond, donating into an empty platinum d SFO, and the platinum $d_{z^{2}}$ SFO π‐donating into an empty π* SFO of the C_2_B_2_ fragment, with a strong B1−C1 bonding component (Figure 5 b). This bonding picture is also reflected in the calculated Mayer bond orders (MBOs) of **4‐Pt** and the metal‐free optimized *cis*‐η^4^‐like structure of **4**, namely ***cis*** **‐4**. While the bond order of the B=B and C1=C2 bonds decrease from 1.50 and 1.75, respectively, in ***cis*** **‐4** to 1.13 and 1.28, respectively, in **4‐Pt**, only a very small increase from 0.87 in ***cis*** **‐4** to 0.89 in **4‐Pt** is observed in the MBO of B1–C1.

In solution, the ^11^B NMR spectrum of **4‐Pt** showed a broad resonance at *δ*=10.6 ppm, which is strongly upfield‐shifted from that of **4** [δ(^11^B)=25.1 ppm] and **4‐Cu** [δ(^11^B)=19.5 ppm], presumably owing to the strong π backdonation of the Pt^0^ center. The room‐temperature ^1^H NMR spectrum showed very broad signals and those for the vinylic protons were undetectable. At low temperature (−90 to −40 °C) at least four different conformers are visible with vinylic proton resonances around *δ*=5 ppm. These conformers could be rapidly exchanging *cis*/*trans*‐η^4^‐C_2_B_2_‐Pt and η^2^‐B_2_‐Pt conformers, in which the C1=C2 and the C21=C28 bonds are alternatingly bound to the Pt center, similarly to the bonding motifs found in 1,3‐diene complexes (Figure 1 a). Moreover, at temperatures above 40 °C **4‐Pt** decomposed rapidly in solution. An attempt to stabilize **4‐Pt** by replacing the remaining nbe ligand with I*i*Pr resulted in complete release of free **4**, as observed by ^11^B and ^1^H NMR spectroscopic analyses (Scheme 3 c; see Figures S34 and S35).

**Scheme 4:**
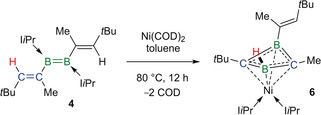
Nickel‐mediated rearrangement and complexation of **4**.

Unlike its reactions with CuCl and Pt(nbe)_3_, the reaction of **4** with Ni(COD)_2_ (COD=1,5‐cyclooctadiene) did not result in simple coordination to the metal center. Instead a complex rearrangement of the B=B unit and one vinyl group took place, resulting in the formation of the NiC_2_B_2_ complex **6** [δ(^11^B)=13.3 ppm] as the major reaction product (Scheme 4).^31^ Unlike for **4‐Pt**, the addition of I*i*Pr to **6** did not result in the liberation of the diborete ligand and no reaction was observed.

The X‐ray crystallographically derived structure of **6** (Figure 6) shows the nickel center bound to all four atoms of a 1‐vinyl‐1,3‐diborete ligand, which displays a butterfly structure with the carbon atoms located at the tips of the wings, a puckering angle of 40.2° and a B–B distance of 1.890(2) Å. Furthermore, the β‐vinyl hydrogen H2 has migrated from C2 to B1 [B1‐H2 1.106(17) Å]^32^ and the two I*i*Pr ligands have migrated from boron to nickel, displacing the COD ligands. The B−C bond lengths are all relatively similar [1.5430(19) to 1.5584(19) Å] and shorter by about 0.05 Å compared to typical B−C bonds, suggesting some π delocalization over the C_2_B_2_ ring. This delocalization is also confirmed by the ^13^C NMR resonances of the *C_2_*B_2_ ring, which appear in the aromatic region at *δ*=132.3 (B_2_ *Ct*Bu) and *δ*=112.0 ppm (B_2_ *C*Me).

**Figure 7:**
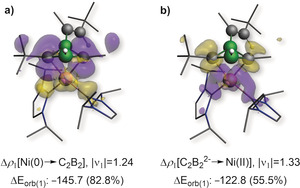
Plot of the main deformation densities of **6** (B3LYP/TZV2P) considering a) Ni^0^ and neutral C_2_B_2_ fragments (total Δ*E*_orb_=−175.9 kcal mol^−1^) and b) Ni^II^ and [C_2_B_2_]^2−^ fragments (total Δ*E*_orb_=−221.3 kcal mol^−1^). The |ν_k_| values correspond to the eigenvalues of the complementary eigenfunctions (*ψ*_−k_, *ψ*_k_) in the NOCV representation, Δ*E*_orb(k)_ is the *k* ^th^ orbital interaction energy (kcal mol^−1^), with the percentage contribution to the total orbital interaction energy (Δ*E*_orb_) shown in parentheses. The electron density flows from yellow to purple.

To assess the electronic situation of **6**, the nature of bonding was examined by EDA‐NOCV. Two distinct scenarios were assessed: a) The donor‐acceptor interaction of a Ni^0^ fragment with a neutral 2π‐electron 1,3‐diborete ligand, and b) the interaction of a Ni^II^ center and a dianionic 4π‐electron [C_2_B_2_]^2−^ ligand. The calculations reveal that, irrespective of the choice of fragments, the main bonding contribution arises from σ interactions between Ni and the carbon atoms of the C_2_B_2_ ring (Figure 7). The scenario involving Ni^0^ and a neutral C_2_B_2_ 1,3‐diborete, however, yields a lower absolute value of the total orbital interaction energy, Δ*E*_orb_ (Figure 7; see Table S5),^33^ which indicates a more appropriate choice of fragments. These data suggest that the bonding in **6** is best described as the result of the Ni^0^ fragment donating into an empty π* SFO of the neutral diborete ligand located at the carbon centers. This donor–acceptor interaction accounts for more than 80 % of Δ*E*_orb_, thereby suggesting that the valence electrons of the C_2_B_2_ ring are bystanders. The calculated MBOs of roughly unity for all endocyclic B−C bonds in **6** suggest delocalization of the two π electrons over the C_2_B_2_ ring despite the lack of planarity. Furthermore, the MBO of only 0.25 for B1–B2 confirms the absence of B−B bonding. The complex **6** may also be viewed as a 22 electron C_2_B_2_Ni *closo*‐cluster according to the Wade–Mingos rules and is the smallest nickel‐carborane cluster reported to date. The average bond lengths within the C_2_B_2_Ni fragment [Ni‐C_(avg)_ 1.99; Ni‐B_(avg)_ 2.25; B⋅⋅⋅B 1.890(2); B‐C_(avg)_ 1.55 Å] are within the range of other nickel carborane clusters.^34^

**Figure 8:**
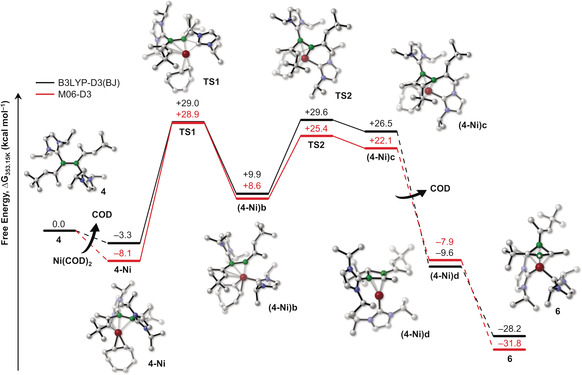
Relative Gibbs free‐energy profile at 353.15 K (reaction temperature) of a plausible mechanism for the reaction of **4** to **6** at the B3LYP‐D3(BJ)/def2‐TZVPP+SMD(toluene) and M06‐D3/def2‐TZVPP+SMD(toluene) levels. Dashed lines indicate parts in which the transformation is not an elementary step.

Considering the number of strong bonds broken (one C=C bond and the B=B bond, one C−H and two B−C bonds, as well as four Ni–COD π interactions) and reformed (three B−C, one B−H, two Ni−B, and four Ni−C bonds) during the formation of **6**, the reaction is surprisingly selective.^31^ We therefore decided to undertake a computational analysis of the mechanism of formation of **6** at two different levels of theory, the results of which are shown in Figure 8 (see the Supporting Information for details). We propose that in the first step, Ni coordinates to the divinyldiborene in an analogous manner to Pt, yielding the slightly favorable intermediate **4‐Ni**. The next step, which is the rate‐determining one, consists of the migration of the first NHC ligand to the Ni center and liberation of one molecule of COD. This step is followed by an intramolecular [2+2] cycloaddition of the alkene moiety to the B=B bond, starting from intermediate **(4‐Ni)b** and leading to the 1,2‐diborete complex **(4‐Ni)c**. While a handful of cycloaddition reactions of alkynes to B–B multiple bonds accompanied by C_2_B_2_ rearrangements have been reported,^35^ this is the first example of cycloaddition of an alkene to a B‐B multiple bond. The rearrangement of **(4‐Ni)c** to its 1,3‐diborete isomer **(4‐Ni)d** may be expected: extensive experimental studies in the 1980s^36^, ^37^ and later computational investigations^38^ have shown that in the absence of electronic stabilization 1,2‐diboretes rearrange to their thermodynamically more stable 1,3‐isomers. The final formation of **6** by migration of the second NHC to Ni and of H2 from C2 to B1 is calculated to be highly favorable from a thermodynamic point of view (Δ*G*=−28.2 kcal mol^−1^ at the B3LYP‐D3(BJ)/def2‐TZVPP level), and the barrier heights obtained are consistent with a reaction temperature of 80 °C.

## Conclusion

The synthesis of **4** from the reductive coupling of two NHC‐stabilized (dibromo)vinylboranes was only rendered possible by suppressing *β*‐carbon radical recombination through the introduction of a sterically hindering *tert*‐butyl group in this position. While formally isoelectronic to a 1,3,5‐hexatriene, experimental and theoretical data show that **4** does not display any delocalization of π electron density over the C_2_B_2_C_2_ core. Calculations show that this lack of delocalization is mainly a result of the sterics of the methyl groups at the α‐vinyl positions preventing planarization.

We have shown that the coordination mode of such a 3,4‐dibora‐1,3,5‐hexatriene is strongly dependent on the nature of the metal used, unexpectedly resulting in three different outcomes with three different late transition metals. Whereas with CuCl, **4** forms a typical π‐diborene complex, it coordinates to Pt^0^ in a fashion reminiscent of 1,3‐dienes by forming a *cis*‐η^4^‐vinyldiborene complex, the coordination of which is fluxional in solution. EDA‐NOCV calculations show that, despite a stronger degree of planarization in the metal‐bound C_2_B_2_ unit, there still is little delocalization of the π electron density: π donation to platinum occurs mostly from the B=B double bond, while the Pt center π‐backdonates into the empty π* orbital of the C_2_B_2_ ligand. In contrast, coordination of the vinyldiborene unit to a Ni^0^ complex induces a complex rearrangement into an η^4^‐1,3‐diborete complex, which proceeds by a novel metal‐templated cycloaddition of the alkene moiety to the adjacent diborene.

This study demonstrates once more that the replacement of a C=C bond by an isoelectronic, yet much more electron‐rich B=B bond considerably alters the chemistry of the resulting olefin analogue, opening up new avenues for reactivity. Furthermore, the hitherto undocumented coordination of B=B bonds to group 10 metals known for their catalytic performance in olefin functionalization is promising for future applications in catalytic diborene functionalization reactions.

## Conflict of interest

The authors declare no conflict of interest.

## Supporting information

As a service to our authors and readers, this journal provides supporting information supplied by the authors. Such materials are peer reviewed and may be re‐organized for online delivery, but are not copy‐edited or typeset. Technical support issues arising from supporting information (other than missing files) should be addressed to the authors.

Supplementary
