# Supplementary material for: Oxidation, Coordination, and Nickel‐Mediated Deconstruction of a Highly Electron‐Rich Diboron Analogue of 1,3,5‐Hexatriene
Source: Angew Chem Int Ed Engl. 2020 Jul 1;59(36):15717–25. doi: 10.1002/anie.202006131 (PMC7497145; doi:10.1002/anie.202006131)
Supplement: Supplementary file 1 — Supplementary [file ANIE-59-15717-s001.pdf]

Angewandte Chemie
Eine Zeitschrift der Gesellschaft Deutscher Chemiker

Supporting Information

Oxidation, Coordination, and Nickel-Mediated Deconstruction of a Highly Electron-Rich Diboron Analogue of 1,3,5-Hexatriene

*Alexander Hermann+, Felipe Fantuzzi+, Merle Arrowsmith, Theresa Zorn, Ivo Krummenacher, Benedikt Ritschel, Krzysztof Radacki, Bernd Engels, and Holger Braunschweig**

anie_202006131_sm_miscellaneous_information.pdf

Supporting information

Table of Contents

General Considerations 2

Synthetic procedures 3

NMR spectra of isolated new compounds.. 10

NMR spectra of reaction mixtures 49

EPR spectra 52

UV-vis spectra... 54

IR spectra... 56

Crystallographic details... 59

Computational details... 64

References 124

General Considerations

All manipulations were performed either under an atmosphere of dry argon or *in vacuo* using standard Schlenk line or glovebox techniques. Deuterated solvents were dried over molecular sieves and degassed by three freeze-pump-thaw cycles prior to use. All other solvents were distilled and degassed from appropriate drying agents. Solvents (both deuterated and non-deuterated) were stored under argon over activated 4 Å molecular sieves. NMR spectra were acquired on a Bruker Avance 400 NMR spectrometer (^{1}H: 400.1 MHz, ^{11}B: 128.4 MHz, ^{13}C: 100.6 MHz) or on a Bruker Avance 500 NMR spectrometer (^{1}H: 500.1 MHz, ^{1}H: 400.1 MHz, ^{11}B{^{1}H}: 160.5 MHz, ^{13}C{^{1}H}: 125.8 MHz). Chemical shifts (δ) are given in ppm and internally referenced to the carbon nuclei (^{13}C{^{1}H}) or residual protons (^{1}H) of the solvent. ^{11}B{^{1}H} NMR spectra were referenced to [$BF_3 \cdot OEt_2$] as an external standard. ^{1}H NMR and ^{13}C{^{1}H} NMR resonances were assigned with assistance from DEPT-135, HSQC, HMBC, and HMQC experiments. Microanalyses (C, H, N) were performed on an Elementar vario MICRO cube elemental analyzer. UV-vis spectra were acquired on a JASCO-V660 UV-vis spectrometer. Cyclic voltammetry experiments were performed using a Gamry Instruments Reference 600 potentiostat. A standard three-electrode cell configuration was employed using a platinum disk working electrode, a platinum wire counter electrode, and a silver wire, separated by a *Vycor* tip, serving as the reference electrode. Formal redox potentials are referenced to the ferrocene/ferrocenium (Fc/Fc^{+}) redox couple. Tetra-*n*-butylammonium hexafluorophosphate ([nBu$_4$N][PF_6]) was employed as the supporting electrolyte. Compensation for resistive losses (*iR* drop) was employed for all measurements.

Solvents and reagents were purchased from Sigma Aldrich or Alfa Aesar. Deuterated solvents were degassed with three freeze-pump-thaw cycles and stored over molecular sieves in J. Young-style ampoules or in a glovebox. I*i*Pr (1,3-di*iso*propylimidazol-2-ylidene),[1] HBCat[2] and Pt(nbe)$_3$[3] were synthesized using literature procedures. Potassium graphite (KC_8) was prepared by heating graphite (20.36 g, 1.695 mol; first heated to 180 °C under reduced pressure for ten hours) and freshly-cut potassium (8.35 g, 214 mmol) at 180 °C with stirring for two days, followed by filtering the powder through a tea strainer.

Synthetic procedures

(Me)HC=C(Me)BCat

Neat HBCat (3.38 g, 28.0 mmol, 3.00 mL) and 2-butyne (1.59 g, 29.3 mmol, 2.30 mL) were mixed in a Young flask. The flask was closed and the reaction mixture heated to 75 °C for 4 h. (Me)HC=C(Me)BCat was isolated by short path distillation at 70 °C ($1\cdot10^{-3}$ mbar) as a colorless liquid (4.23 g, 24.3 mmol, 87%). ^{1}H NMR (500.1 MHz, 296 K, C_6D_6): δ = 7.06–7.02 (m, 2H, Cat*H*), 6.89 (dq, ^{3}J = 6.7 Hz, ^{4}J = 1.5 Hz, 1H, *H*C=C), 6.81–6.77 (m, 2H, Cat*H*), 1.84 (m, 3H, CH_3), 1.55 (dq, ^{3}J = 6.77 Hz, ^{4}J = 1.2 Hz, 3H, CH_3) ppm. ^{11}B{^{1}H} NMR (160.5 MHz, 296 K, C_6D_6): δ = 31.9 (s) ppm.

(Me)HC=C(Me)BCat(I*i*Pr)

A solution of I*i*Pr (1.45 g, 9.55 mmol) in hexane (5 mL) was added slowly to a solution of (Me)HC=C(Me)BCat (1.66 g, 9.55 mmol) in hexane (20 mL) at 0 °C. The resulting yellow suspension was warmed to rt and stirred for an hour. The solid was collected and washed with hexane (3 x 15 mL). (Me)HC=C(Me)BCat(I*i*Pr) was isolated as a light yellow solid (2.75 g, 8.43 mmol, 88%). ^{1}H NMR (500.1 MHz, 296 K, C_6D_6): δ = 7.04–7.01 (m, 2H, Cat*H*), 6.87–6.83 (m, 2H, Cat*H*), 6.08 (s, 2H, C*H*=C*H*), 5.61–5.53 (sept, ^{3}J = 6.8 Hz, 2H, C*H*(CH_3)$_2$), 5.26 (dq, ^{3}J = 6.5 Hz, ^{4}J = 1.5 Hz, 1H, *H*C=C), 2.06 (m, 3H, CH_3), 1.76 (dq, ^{3}J = 6.5 Hz, ^{4}J = 1.03 Hz, 3H, CH_3), 0.95 (d, ^{3}J = 6.7 Hz, 12H, CH(CH_3)$_2$) ppm. ^{13}C{^{1}H} NMR (12 5.8 MHz, 296 K, C_6D_6): δ = 154.9 (Ar-*C*-O), 124.6 (H*C*=C), 118.6 (Cat-*C*H), 116.1 (*C*H=*C*H), 109.2 (Cat-*C*H), 49.9 (N*C*H(CH_3)$_2$), 23.1 (CH(*C*H_3)$_2$), 14.2 (*C*H_3), 14.1 (*C*H_3) ppm. ^{11}B{^{1}H} NMR (160.5 MHz, 296 K, C_6D_6): δ = 9.93 (s) ppm. Elemental analysis: calculated for $C_{19}H_{27}BN_2O_2$: C 69.95, H 8.34, N 8.59; found: C 69.98, H 8.34, N 8.16%.

(Me)HC=C(Me)BBr$_2$(I*i*Pr), 1-Me

Neat BBr_3 (2.04 g, 8.12 mmol) was added slowly to a solution of (Me)HC=C(Me)BCat(I*i*Pr) (2.65 g, 8.12 mmol) in hexane (40 mL) at 0 °C. The mixture was stirred for 15 min, warmed to rt and stirred for another hour. The supernatant was decanted and the oily orange residue dried and extracted with toluene (2 x 15 mL). The toluene solution was concentrated *in vacuo* and stored at –30 °C overnight. **1-Me** was isolated as a colorless crystalline solid (2.40 g, 6.35 mmol, 78%). ^{1}H NMR (500.1 MHz, 296 K, C_6D_6): δ = 6.53 (dq, ^{3}J = 6.7 Hz, ^{4}J = 1.2 Hz, 1H, *H*C=C), 6.14 (s, 2H, C*H*=C*H*), 5.93–5.85 (sept, ^{3}J = 6.7 Hz, 2H, C*H*(CH_3)$_2$), 2.08 (m, 3H, CH_3), 1.24 (s, 9H, CCH_3), 1.74 (dq, ^{3}J = 6.7 Hz, ^{4}J = 1.0 Hz, 3H, CH_3), 0.97 (d, ^{3}J = 6.8 Hz,

12H, CH(CH_3)$_2$) ppm. $^{13}C\{^1H\}$ NMR (125.8 MHz, 296 K, C_6D_6): δ = 129.2 (H*C*=C), 117.4 (*C*H=*C*H), 50.4 (N*C*H(CH$_3$)$_2$), 22.9 (CH(*C*H$_3$)$_2$), 17.8 (*C*H$_3$), 14.9 (*C*H$_3$) ppm. $^{11}B\{^1H\}$ NMR (160.5 MHz, 296 K, C_6D_6): δ = –3.2 (s) ppm. Elemental analysis: calculated for $C_{13}H_{23}BN_2Br_2$: C 41.31, H 6.13, N 7.41; found: C 41.46, H 6.07, N 7.23%.

(I*i*Pr)BrB=C(Me)CH(Me)CH(Me)C(Me)=BBr(I*i*Pr), 2-Me

1-Me (1.00 g, 2.65 mmol) and KC_8 (0.90 g, 6.66 mmol) were combined in benzene (20 mL). The suspension was stirred for 6 h at rt, filtered and the residue washed with benzene (2 x 5 mL). After removal of the solvent in *vacuo*, the crude product was isolated as a light red solid (0.38 g, 6.35 mmol, 39%). Although single crystals of **2-Me** suitable for X-ray structural analysis were obtained from a concentrated benzene solution, the isolation of analytically pure **2-Me** failed despite several recrystallization attempts. $^{11}B\{^1H\}$ NMR (128.4 MHz, 296 K, C_6H_6): δ = 12.5 (br s) ppm.

[(I*i*Pr)B=C(Ph)CH(Ph)]$_2$, 3-Me

Sodium powder (38.4 mg, 1.68 mmol) and crude **2-Me** (100 mg, 0.17 mmol) were combined in DME (2 mL), whereupon the suspension turned deep pink. After 6 h stirring at rt, the reaction mixture was filtered and the solvent was removed in vacuo. The isolation of analytically pure **3-Me** did not succeed due to the presence of large amounts of byproducts. $^{11}B\{^1H\}$ NMR (128.4 MHz, 296 K, C_6H_6): δ = 19.8 (br s) ppm.

(Ph)HC=C(Ph)BCat(I*i*Pr)

Diphenylacetylene (1.67 g, 9.55 mmol) and [($Ph_3P)_3$RhCl] (87.8 mg, 95.5 μmol) were combined in benzene (20 mL) and HBCat (1.13 g, 9.55 mmol) was added. After heating the reaction mixture for 4 h to 60 °C, insoluble materials were removed by filtration and a solution of I*i*Pr (1.45 g, 9.55 mmol) in hexane (25 mL) was added slowly. The resulting yellow suspension was stirred for 40 min, filtered and washed with hexane (3 x 20 mL). (Me)HC=C(Me)BCat(I*i*Pr) was isolated as a light yellow solid (3.34 g, 8.43 mmol, 78%). ^1H NMR (500.1 MHz, 296 K, C_6D_6): δ = 7.42 (s, 1H, *H*C=C), 7.21–7.17 (m, 3H, Ph-*H*), 7.10–7.08 (m, 2H, Ph-*H*), 7.10–7.08 (m, 2H, Cat*H*), 7.00–6.95 (m, 4H, Ph-*H*), 6.88–6.86 (m, 2H, Cat*H*), 6.88–6.86 (m, 1H, Ph-*H*), 5.98 (s, 2H, C*H*=C*H*), 5.55–5.47 (sept, ^3J = 6.8 Hz, 2H, C*H*(CH$_3$)$_2$), 0.85 (d, ^3J = 6.6 Hz, 12H, CH(CH_3)$_2$) ppm. $^{13}C\{^1H\}$ NMR (125.8 MHz, 296 K, C_6D_6): δ = 154.2 (Ar-*C*-O), 146.2 (Ph-C_q), 139.6 (Ph-C_q), 131.0 (H*C*=C), 129.9, 128.6, 126.2, 125.2 (Ph-*C*), 119.0 (Cat-*C*H), 116.1 (*C*H=*C*H), 109.7 (Cat-*C*H), 501.0 (N*C*H(CH$_3$)$_2$), 23.1

(CH(CH_3)$_2$) ppm. ^{11}B{^{1}H} NMR (160.5 MHz, 296 K, C_6D_6): δ = 9.11 (s) ppm. Elemental analysis: calculated for $C_{19}H_{31}BN_2O_2$: C 77.34, H 6.94, N 6.22; found: C 77.58, H 7.06, N 5.79%.

(Ph)HC=C(Ph)BBr$_2$(I*i*Pr), 1-Ph

A suspension of (Ph)HC=C(Ph)BCat(I*i*Pr) (2.7 g, 5.99 mmol) in hexane (40 mL) was cooled to –40 °C and neat BBr_3 (1.50 g, 5.99 mmol) was added slowly. After stirring for 20 min, the reaction mixture was warmed to rt and stirred for another hour. The precipitate was collected by centrifugation, washed with hexane (2 x 20 mL) and dried in vacuo. The crude product was extracted with toluene (4 x 20 mL) and the solution concentrated. Colorless crystals of **1-Ph** were isolated after storing the solution at –30 °C overnight (1.50 g, 2.99 mmol, 50%). ^{1}H NMR (500.1 MHz, 296 K, C_6D_6): δ = 7.42 (s, 1H, *H*C=C), 7.35–7.33 (m, 2H, Ph-*H*), 7.15–7.09 (m, 4H, Ph-*H*), 7.06–7.03 (m, 1H, Ph-*H*), 6.97–6.94 (m, 2H, Ph-*H*), 6.88–6.85 (m, 1H, Ph-*H*), 6.21 (s, 2H, C*H*=C*H*), 6.18–6.10 (sept, ^3J = 6.6 Hz, 2H, C*H*(CH$_3$)$_2$), 0.96 (d, ^3J = 6.6 Hz, 12H, CH(CH_3)$_2$) ppm. ^{13}C{^{1}H} NMR (125.8 MHz, 296 K, C_6D_6): δ = 144.3 (Ph-C_q), 139.3 (Ph-C_q), 134.5 (H*C*=C), 130.1, 129.9, 128.3, 128.2, 126.6, 126.2 (Ph-*C*), 117.6 (*C*H=*C*H), 51.1 (N*C*H(CH$_3$)$_2$), 23.0 (CH(CH_3)$_2$) ppm. ^{11}B{^{1}H} NMR (160.5 MHz, 296 K, C_6D_6): δ = –4.2 (s) ppm. Elemental analysis: calculated for $C_{23}H_{27}BN_2Br_2$: C 55.02, H 5.42, N 5.58; found: C 55.16, H 5.46, N 5.51%.

(I*i*Pr)BrB=C(Ph)CH(Ph)CH(Ph)C(Ph)=BBr(I*i*Pr), 2-Ph

(Ph)HC=C(Ph)BBr$_2$(I*i*Pr) (50.0 mg, 99.6 μmol) and KC_8 (40.4 mg, 299 μmol) were combined in benzene (2 mL) and the reaction mixture was stirred for 6 h at rt. After filtration and slow evaporation of the solvent, crystals of **2-Ph** suitable for X-ray structure analysis were obtained. The isolation of an analytically pure sample of **2-Ph** failed despite multiple recrystallization attempts and washings. ^{11}B{^{1}H} NMR (128.4 MHz, 296 K, C_6H_6): δ = 18.4 (br s) ppm.

[(I*i*Pr)B=C(Ph)CH(Ph)]$_2$, 3-Ph

1-Ph (50.0 mg, 99.6 μmol) and KC_8 (135 mg, 996 μmol) were combined in benzene (2 mL) and the reaction mixture was stirred for 6 h at rt. After filtration and slow evaporation of the solvent crystals of **3-Ph** suitable for X-ray structural analysis could be obtained. The isolation of analytically pure substance failed despite multiple recrystallization attempts and washings. ^{11}B{^{1}H} NMR (128.4 MHz, 296 K, C_6H_6): δ = 27.5 (br s) ppm.

(tBu)HC=C(Me)BCat

The experimental procedure was carried out following a known synthetic method.[4] Neat HBCat (2.00 g, 16.7 mmol, 1.78 mL) and 4,4-dimethyl-2-pentyne (1.60 g, 16.7 mmol, 2.23 mL) were combined and the reaction mixture was heated for 4 h to 75 °C in a closed round-bottom flask. (tBu)HC=C(Me)BCat was isolated by short path distillation at 90 °C ($1{\cdot}10^{-3}$ mbar) as a colorless liquid (2.27 g, 10.0 mmol, 63%). ^{1}H NMR (400.1 MHz, 296 K, C_6D_6): δ = 7.07–7.03 (m, 2H, Cat*H*), 6.89 (s, 1H, *H*C=C), 6.82–6.78 (m, 2H, Cat*H*), 1.99 (s, 3H, C=CCH_3), 1.10 (s, 9H, C(CH_3)$_3$) ppm. ^{11}B{^{1}H} NMR (128.4 MHz, 296 K, C_6D_6): δ = 32.6 (s) ppm.

(tBu)HC=C(Me)BCat(IiPr)

A solution of IiPr (1.60 g, 10.0 mmol) in hexane (5 mL) was added slowly to a solution of (tBu)HC=C(Me)BCat (2.27g, 10.0 mmol) in hexane (30 mL) at –40 °C. The resulting yellow suspension was warmed to rt, stirred for an hour and filtered. After washing the solid with hexane (2 x 15 mL) (tBu)HC=C(Me)BCat(IiPr) was isolated as a colorless solid (2.96 g, 8.04 mmol, 80%). ^{1}H NMR (500.1 MHz, 296 K, C_6D_6): δ = 7.04–7.01 (m, 2H, Cat*H*), 6.87–6.83 (m, 2H, Cat*H*), 6.05 (s, 2H, C*H*=C*H*), 5.62–5.54 (sept, ^{3}J = 6.7 Hz, 2H, C*H*(CH$_3$)$_2$), 5.06 (s, 1H, *H*C=C), 2.17 (d, ^{4}J = 1.6 Hz, 3H, C=CCH_3), 1.22 (s, 9H, C=CC(CH_3)$_3$), 0.97 (d, ^{3}J = 6.7 Hz, 12H, CH(CH_3)$_2$) ppm. ^{13}C{^{1}H} NMR (125.8 MHz, 296 K, C_6D_6): δ = 154.9 (Ar-*C*-O), 141.1 (H*C*=C), 118.6 (Cat-*C*H), 116.1 (*C*H=*C*H), 109.2 (Cat-*C*H), 49.8 (N*C*H(CH$_3$)$_2$), 33.6 (*C*(CH$_3$)$_3$), 31.7 (C(*C*H$_3$)$_3$), 23.0 (CH(*C*H$_3$)$_2$), 15.1 (C=C*C*H$_3$) ppm. ^{11}B{^{1}H} NMR (160.5 MHz, 296 K, C_6D_6): δ = 10.1 (s) ppm. Elemental analysis: calculated for $C_{22}H_{33}BN_2O_2$: C 71.74, H 9.03, N 7.61; found: C 71.80, H 9.01, N 7.59%.

(tBu)HC=C(Me)BBr$_2$(IiPr), 1-tBu

A suspension of (tBu)HC=C(Me)BCat(IiPr) (2.83 g, 7.68 mmol) in hexane (40 mL) was cooled to –70 °C and neat BBr$_3$ (1.92 g, 7.68 mmol) was added slowly. The mixture was stirred for an hour and warmed to rt. After stirring for another hour, the supernatant was decanted and the orange oily residue was dried in *vacuo*. After extraction with toluene (2 x 15 mL), the solution was concentrated and stored overnight at –30 °C. After crystallization, **1-tBu** was obtained as a colorless solid (2.08 g, 4.95 mmol, 64%). ^{1}H NMR (500.1 MHz, 296 K, C_6D_6): δ = 6.53 (q, ^{4}J = 1.2 Hz, 1H, *H*C=C), 6.15 (s, 2H, C*H*=C*H*), 6.01–5.93 (sept, ^{3}J = 6.7 Hz, 2H, C*H*(CH$_3$)$_2$), 2.17 (d, ^{4}J = 1.3 Hz, 3H, C=CCH_3), 1.24 (s, 9H, C=CC(CH_3)$_3$), 1.00 (d, ^{3}J = 6.7 Hz, 12H, CH(CH_3)$_2$) ppm. ^{13}C{^{1}H} NMR (125.8 MHz, 296 K, C_6D_6): δ = 144.3 (H*C*=C), 117.4 (*C*H=*C*H), 50.3 (N*C*H(CH$_3$)$_2$), 33.7 (*C*(CH$_3$)$_3$), 30.9 (C(*C*H$_3$)$_3$), 22.9 (CH(*C*H$_3$)$_2$), 18.9

(C=CCH_3) ppm. $^{11}B\{^1H\}$ NMR (160.5 MHz, 296 K, C_6D_6): $\delta = -2.7$ (s) ppm. Elemental analysis: calculated for $C_{16}H_{29}BN_2Br_2$: C 45.75, H 6.96, N 6.67; found: C 45.77, H 7.08, N 6.67%.

(*t*Bu)HC=C(Me)(I*i*Pr)B=B(I*i*Pr)(Me)C=CH(*t*Bu), 4

In a round-bottom flask **1-*t*Bu** (1.34 g, 3.19 mmol) and KC_8 (1.72 g, 12.7 mmol) were combined in benzene (35 mL), whereupon the suspension turned red-brown. The reaction mixture was stirred for 5 h at rt, filtered and washed with benzene (3 x 5 mL). The solution was concentrated and freeze-dried. **4** was isolated as dark red-brown solid (0.60 g, 1.15 mmol, 72%). ^1H NMR (500.1 MHz, 296 K, C_6D_6): $\delta = 6.43$ (s, 4H, C*H*=C*H*), 5.82–5.74 (sept, $^3J = 6.7$ Hz, 4H, C*H*(CH_3)$_2$), 4.80 (q, $^4J = 1.2$ Hz, 2H, *H*C=C), 1.89 (d, $^4J = 1.3$ Hz, 6H, C=CCH_3), 1.33 (s, 18H, C=CC(CH_3)$_3$), 1.00 (t, $^3J = 6.7$ Hz, 24H, CH(CH_3)$_2$) ppm. $^{13}C\{^1H\}$ NMR (125.8 MHz, 296 K, C_6D_6): $\delta = 152.2$ (HC=*C*(CH_3)), 132.4 (H*C*=C), 114.0 (*C*H=*C*H), 49.2 (N*C*H(CH_3)$_2$), 33.8 (*C*(CH_3)$_3$), 32.7 (C(*C*H_3)$_3$), 23.7, 22.4 (CH(*C*H_3)$_2$), 22.0 (C=C*C*H_3) ppm. $^{11}B\{^1H\}$ NMR (160.5 MHz, 296 K, C_6D_6): $\delta = 25.1$ (br s) ppm. UV-vis (benzene): $\lambda_{max} = 453$ nm, $\lambda = 573$ nm. IR (solid-state): ν = 2937–2858 (C=CH), 1626–1541 (C=C) cm^{-1}. Elemental analysis: calculated for $C_{32}H_{58}B_2N_4$: C 73.85, H 11.23, N 10.77; found: C 72.90, H 11.19, N 10.75%. LIFDI-MS [$C_{32}H_{58}B_2N_4$]: m/z (calculated): 520.4842 $gmol^{-1}$; m/z (found): 520.4837 $gmol^{-1}$.

[(*t*Bu)HC=C(Me)(I*i*Pr)B=B(I*i*Pr)(Me)C=CH(*t*Bu)][BAr^F_4], $[4]^{\bullet+}[BAr^F_4]^-$

A solution of **4** (10 mg, 19.2 μmol) in *o*-difluorobenzene (0.5 mL) was treated with solid [C_7H_7][BAr^F_4] (18.3 mg, 19.2 μmol) resulting in a red-purple solution. After removal of all volatiles in vacuo, the sticky residue was washed with hexanes (3 x 1 mL), dried and dissolved in Et_2O to be analysed by EPR spectroscopy. The isolation of an analytically pure substance failed despite multiple attempts to crystallize and wash the compound.

[(*t*Bu)HC=C(Me)(I*i*Pr)B=B(I*i*Pr)(Me)C=CH(*t*Bu)][$MesBC_4Ph_4$], $[4]^{\bullet+}[5]^{\bullet-}$

To a solution of 1-mesityl-2,3,4,5-tetraphenylborole (**5**, 9.35 mg, 19.2 µmol) in *o*-difluorobenzene (0.5 mL) were added 10 mg of **4** (19.2 µmol), resulting in a red solution. After removal of all volatiles in vacuo, the sticky residue was washed with hexanes (3 x 1 mL), dried and dissolved in *o*-difluorobenzene to be analysed by EPR spectroscopy. The isolation of an analytically pure substance failed despite multiple attempts to crystallize and wash the compound.

[{(*t*Bu)HC=C(Me)(I*i*Pr)B=B(I*i*Pr)(Me)C=CH(*t*Bu)}CuCl], 4-Cu

4 (20.0 mg, 38.4 μmol) and CuCl (3.8 mg, 38.4 μmol) were combined in benzene (2 mL) and the mixture was stirred for 30 min, whereupon the solution turned orange and a dark solid started to precipitate. Insoluble materials were removed by filtration and **4-Cu** was isolated as a bright yellow, crystalline solid after slow evaporation of the solvent (14.3 mg, 23.1 μmol, 60%). ^{1}H NMR (500.1 MHz, 296 K, C_6D_6): δ = 6.36 (s, 4H, C*H*=C*H*), 6.08–6.00 (sept, ^{3}J = 6.5 Hz, 4H, C*H*(CH_3)$_2$), 5.53–5.45 (sept, ^{3}J = 6.5 Hz, 4H, C*H*(CH_3)$_2$), 4.29 (q, ^{4}J = 1.1 Hz, 2H, *H*C=C), 1.86 (d, ^{4}J = 1.1 Hz, 6H, C=CC*H*$_3$), 1.63 (d, ^{3}J = 6.7 Hz, 6H, CH(C*H*$_3$)$_2$), 1.21 (d, ^{3}J = 6.7 Hz, 6H, CH(C*H*$_3$)$_2$), 1.2 (d, ^{3}J = 6.7 Hz, 6H, CH(C*H*$_3$)$_2$), 1.16 (s, 18H, C=CC(C*H*$_3$)$_3$), 1.08 (d, ^{3}J = 6.7 Hz, 6H, CH(C*H*$_3$)$_2$) ppm. ^{13}C{^{1}H} NMR (125.8 MHz, 296 K, C_6D_6): δ = 150.5 (HC=*C*(CH_3)), 134.9 (H*C*=C), 115.8, 114.7 (*C*H=*C*H), 50.8, 48.6 (N*C*H(CH_3)$_2$), 33.3 (*C*(CH_3)$_3$), 32.0 (C(*C*H$_3$)$_3$), 24.3, 24.0, 23.1 (CH(*C*H$_3$)$_2$), 22.4 (C=C*C*H$_3$), 21.9 (CH(*C*H$_3$)$_2$) ppm. ^{11}B{^{1}H} NMR (160.5 MHz, 296 K, C_6D_6): δ = 19.5 (br s) ppm. UV-vis (toluene): λ_{max} = 305 nm, λ = 345 nm, λ = 417 nm. Elemental analysis: calculated for $C_{32}H_{58}B_2N_4ClCu$: C 62.05, H 9.44, N 9.04; found: C 62.12, H 9.26, N 8.33%. LIFDI-MS [$C_{32}H_{58}B_2N_4CuCl$]: m/z (calculated): 618.3827 gmol^{-1}; m/z (found): 618.3814 gmol^{-1}.

[{(*t*Bu)HC=C(Me)(I*i*Pr)B=B(I*i*Pr)(Me)C=CH(*t*Bu)}Pt(nbe)], 4-Pt

4 (100 mg, 192 μmol) and Pt(nbe)$_3$ (90.6 mg, 192 μmol) were combined in benzene (2 mL) and the mixture was stirred for 3 h, whereupon the solution turned orange-red. Insoluble materials were removed by filtration and the solvent was removed in *vacuo*. The residue was extracted with hexane and the extract stored 48 h at –30 °C product to yield **4-Pt** as a pink-red crystalline solid (60.0 mg, 74.1 μmol, 39%). ^{1}H NMR (500.1 MHz, 296 K, C_6D_6): δ = 6.24 (s, 2H, C*H*=C*H*), 6.20 (s, 2H, C*H*=C*H*), 6.05 (br s, 4H, C*H*(CH_3)$_2$), 3.23 (br s, 1H, nbe-*H*CC*H*), 3.17 (br s, 1H, nbe-*H*CC*H*), 2.74–2.62 (m, 1H, nbe-*H*), 2.40–2.28 (m, 1H, nbe-*H*), 2.19–2.11 (m, 2H, nbe-*H*), 1.90 (m, 2H, *H*C=C), 1.82 (br s, 6H, C=CC*H*$_3$), 1.42 (br s, 18H, C=CC(C*H*$_3$)$_3$), 1.20–1.17 (m, 24H, CH(C*H*$_3$)$_2$), 1.13 (br s, 3H, nbe-*H*), 0.73–0.71 (m, 1H, nbe-*H*). *Note: The rt ^{13}C{^{1}H} NMR spectrum was too broad to analyze, presumably due to fluctuations between conformers. At low T (< –40 °C) ^{1}H NMR spectra showed at least four distinct conformers, while at higher T (> 40 °C)* ***4-Pt*** *decomposed rapidly, making the analysis of the corresponding ^{13}C{^{1}H} NMR spectra impossible.* ^{11}B{^{1}H} NMR (128.4 MHz, 296 K, C_6D_6): δ = 10.6 (br s) ppm. UV-vis (toluene): λ_{max} = 340 nm, λ = 421 nm, λ = 529 nm. IR (solid state): ν = 2938–2856 (C=CH), 1635–1518 (C=C) cm^{-1}. Elemental analysis: calculated for $C_{39}H_{68}B_2N_4Pt$: C

57.85, H 8.47, N 6.92; found: C 57.85, H 8.68, N 6.88%. LIFDI-MS [$C_{39}H_{68}B_2N_4Pt$]: m/z (calculated): 809.5272 gmol^{-1}; m/z (found): 809.5268 gmol^{-1}.

[(*t*Bu)HC=C(Me){BC(Me)B(H)C(*t*Bu)]}Ni(I*i*Pr)$_2$], 6

4 (100 mg, 192 μmol) and Ni(COD)$_2$ (52.8 mg, 192 μmol) were combined in toluene (3 mL). The mixture was heated overnight to 80 °C and the solvent removed in *vacuo*. The residue was extracted with hexane (3 x 2 mL). The solvent was evaporated slowly at –30 °C to yield **6** as a colorless crystalline solid (28.7 mg, 49.6 μmol, 26%). ^{1}H NMR (500.1 MHz, 296 K, C_6D_6): δ = 6.47 (d, ^3J = 2.0 Hz, 1H, C*H*=C*H*), 6.40 (d, ^3J = 2.0 Hz, 1H, C*H*=C*H*), 6.30 (d, ^3J = 2.0 Hz, 1H, C*H*=C*H*), 6.29 (d, ^3J = 2.0 Hz, 1H, C*H*=C*H*), 5.93 (q, ^4J = 1.5 Hz, 1H, *H*C=C), 5.50–5.41 (sept, ^3J = 6.7 Hz, 2H, C*H*(CH$_3$)$_2$), 5.11–5.03 (sept, ^3J = 6.7 Hz, 1H, C*H*(CH$_3$)$_2$), 5.01–4.93 (sept, ^3J = 6.7 Hz, 1H, C*H*(CH$_3$)$_2$), 4.57 (br s, 1H, B*H*), 2.20 (d, ^4J = 1.8 Hz, 3H, C=CC*H*$_3$), 1.94 (s, 3H, BC(C*H*$_3$)B), 1.61 (s, 9H, BC(CC*H*$_3$)$_3$B), 1.42 (d, ^3J = 6.6 Hz, 3H, CH(C*H*$_3$)$_2$), 1.40 (s, 9H, C=CC(C*H*$_3$)$_3$), 1.33 (d, ^3J = 6.6 Hz, 3H, CH(C*H*$_3$)$_2$), 1.22 (d, ^3J = 6.9 Hz, 3H, CH(C*H*$_3$)$_2$), 1.21 (d, ^3J = 6.5 Hz, 3H, CH(C*H*$_3$)$_2$), 1.16 (d, ^3J = 6.9 Hz, 3H, CH(C*H*$_3$)$_2$), 1.00 (d, ^3J = 6.9 Hz, 3H, CH(C*H*$_3$)$_2$), 0.64 (d, ^3J = 6.7 Hz, 3H, CH(C*H*$_3$)$_2$), 0.47 (d, ^3J = 6.6 Hz, 3H, CH(C*H*$_3$)$_2$) ppm. ^{13}C{^{1}H} NMR (125.8 MHz, 296 K, C_6D_6): δ = 195.4 (C_{Carbene}), 195.1 (C_{Carbene}), 143.0 (H*C*=C), 132.3 (B*C*(CCH$_3$)$_3$B), 116.8, 116.7, 115.2, 115.0 (*C*H=*C*H), 112.0 (B*C*(CH$_3$)B), 51.0, 50.9, 50.8, 50.2 (N*C*H(CH$_3$)$_2$), 35.8 (BC(C*C*H$_3$)$_3$B), 35.4 (BC(*C*CH$_3$)$_3$B), 33.9 (C=C*C*(CH$_3$)$_3$), 31.9 (C=CC(*C*H$_3$)$_3$), 24.3, 24.1, 23.5, 23.1, 23.1, 22.7, 22.7, 22.7 (CH(*C*H$_3$)$_2$), 18.7 (BC(*C*H$_3$)B), 18.6 (C=C*C*H$_3$) ppm. ^{11}B{^{1}H} NMR (160.5 MHz, 296 K, C_6D_6): δ = 13.3 (br s) ppm. UV-vis (benzene): λ_{max} = 341 nm. IR (solid state): ν = 2960–2838 (C=CH), 2396 (B–H), 1655–1546 (C=C) cm^{-1}. Elemental analysis: calculated for [$C_{32}H_{58}B_2N_4Ni$ + 0.5 C_6H_{12}]: C 67.67, H 10.38, N 9.02; found: C 67.35, H 10.63, N 9.12%.

NMR spectra of isolated new compounds

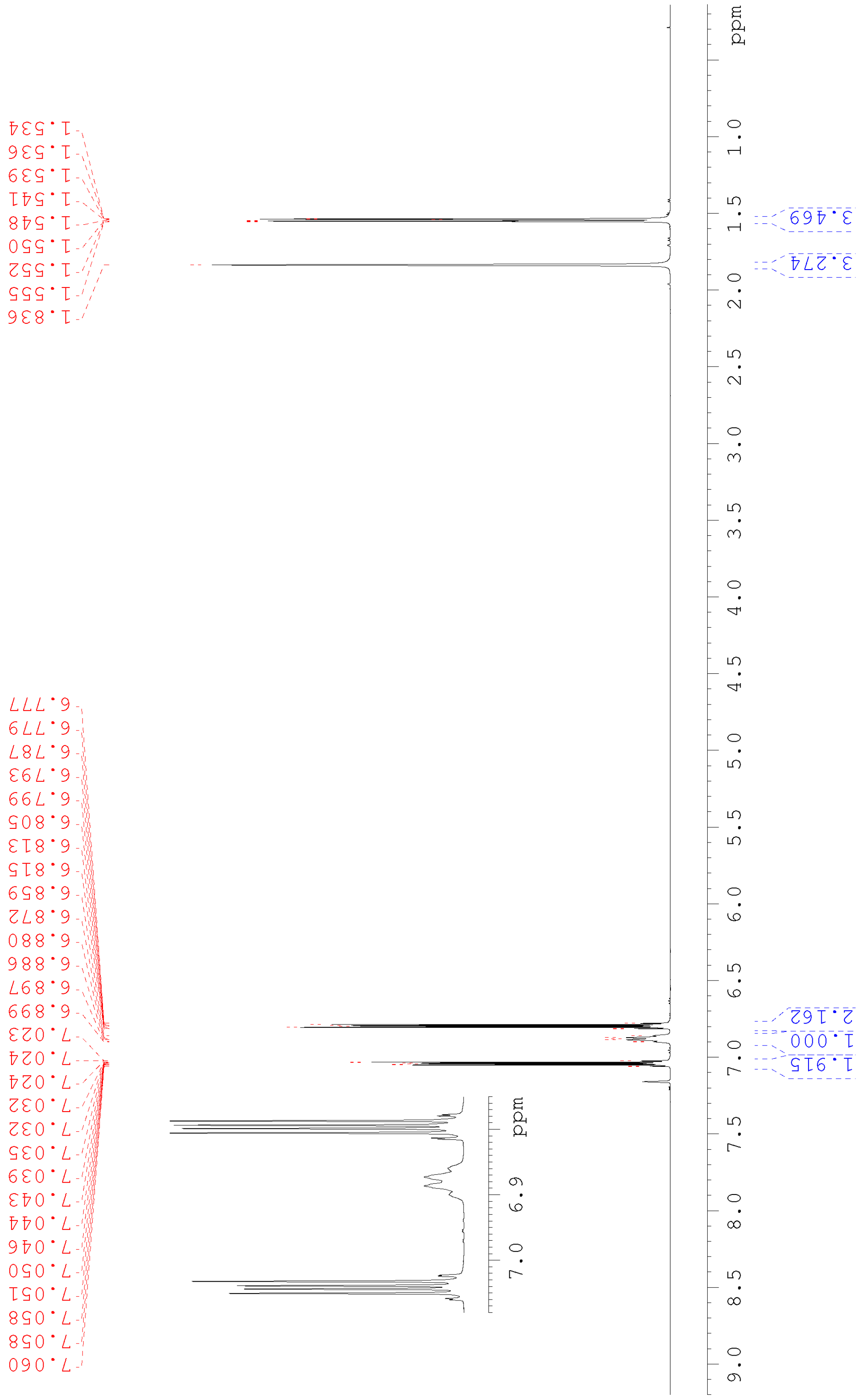

Figure S1. ^{1}H NMR spectrum of **(Me)HC=C(Me)BCat** in C_6D_6.

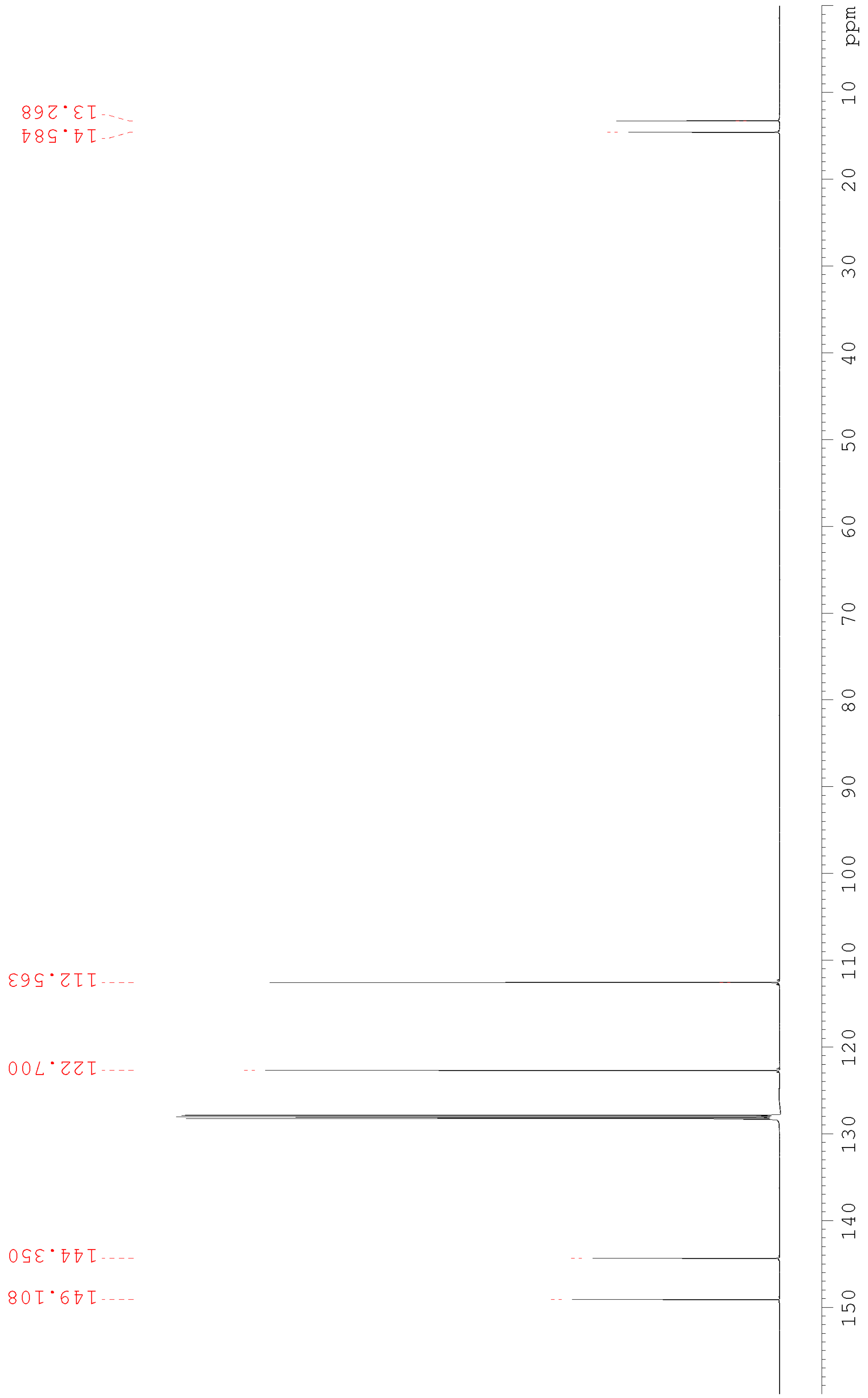

Figure S2. $^{13}C\{^1H\}$ NMR spectrum of **(Me)HC=C(Me)BCat** in C_6D_6.

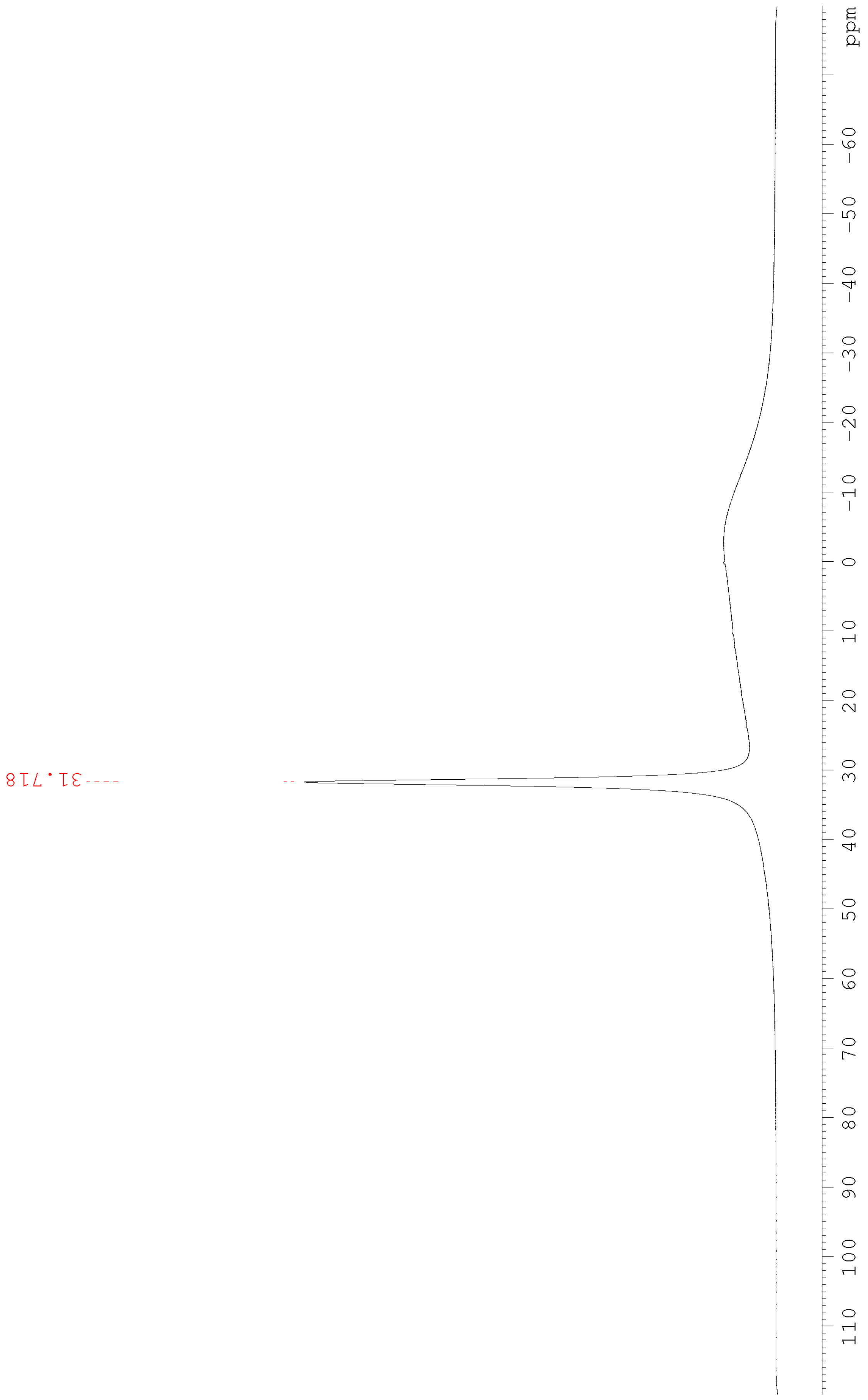

Figure S3. $^{11}B\{^{1}H\}$ NMR spectrum of **(Me)HC=C(Me)BCat** in C_6D_6.

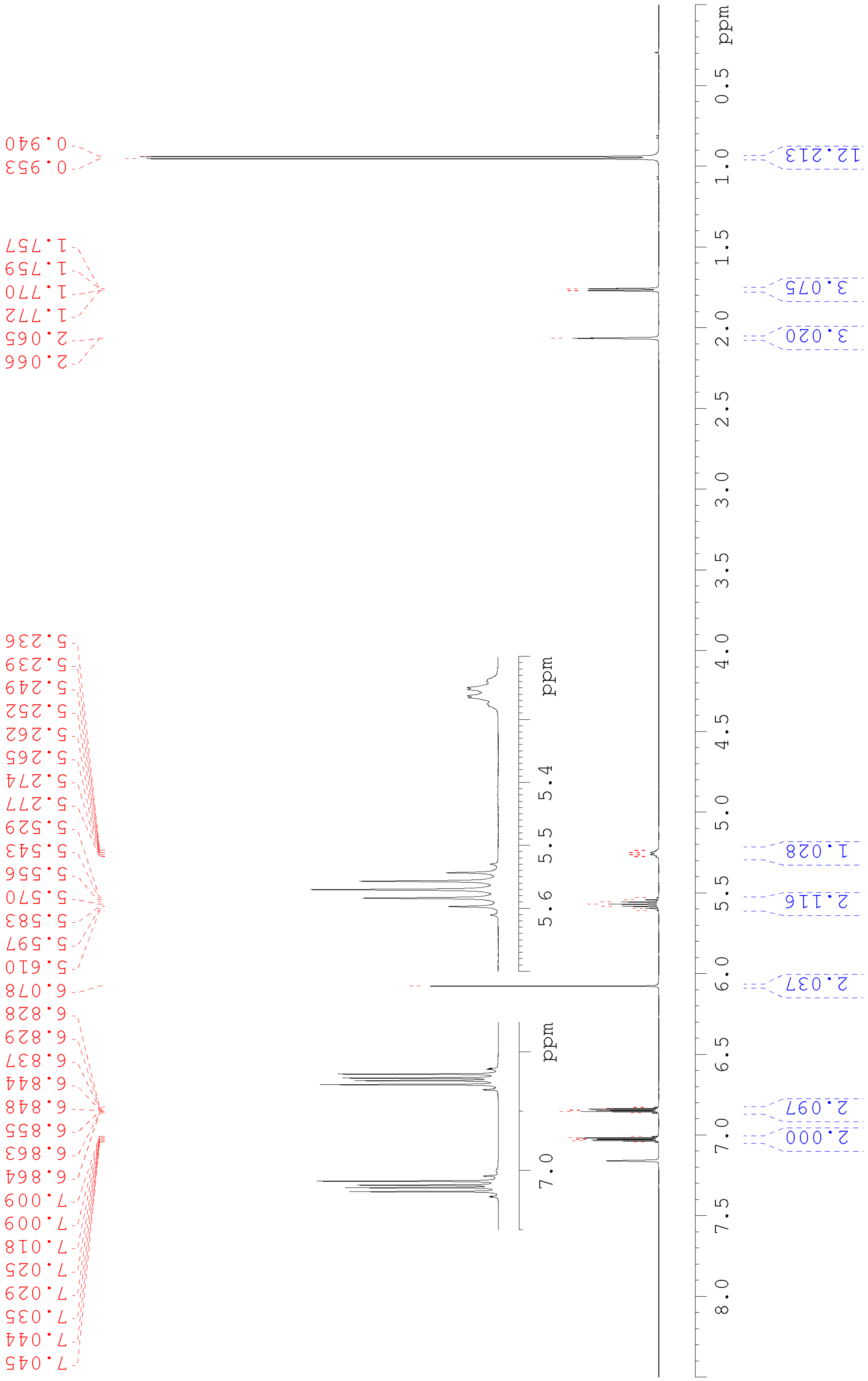

Figure S4. ^{1}H NMR spectrum of **(Me)HC=C(Me)BCat(I*i*Pr)** in C_6D_6.

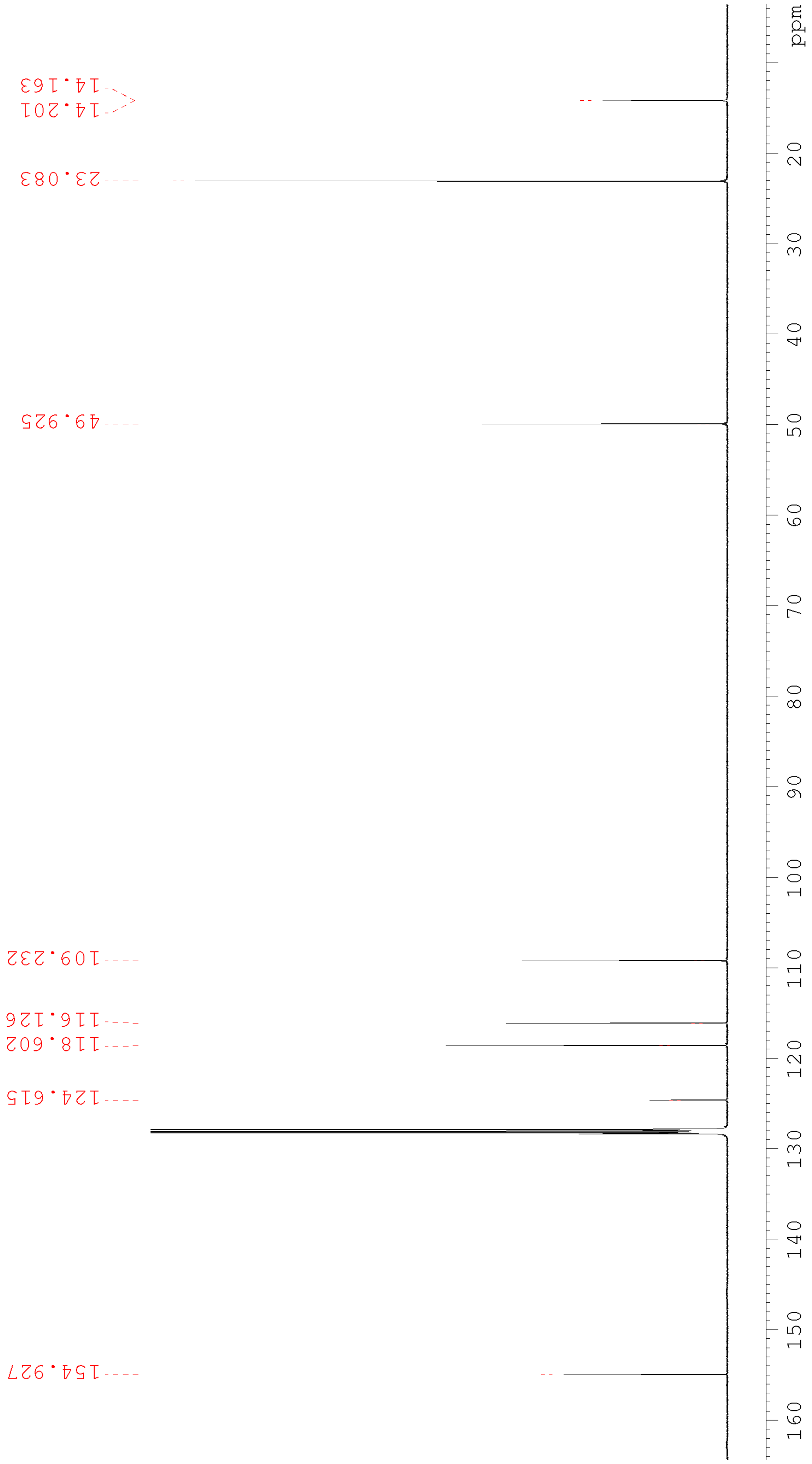

Figure S5. ${}^{13}C\{{}^{1}H\}$ NMR spectrum of **(Me)HC=C(Me)BCat(I*i*Pr)** in C_6D_6.

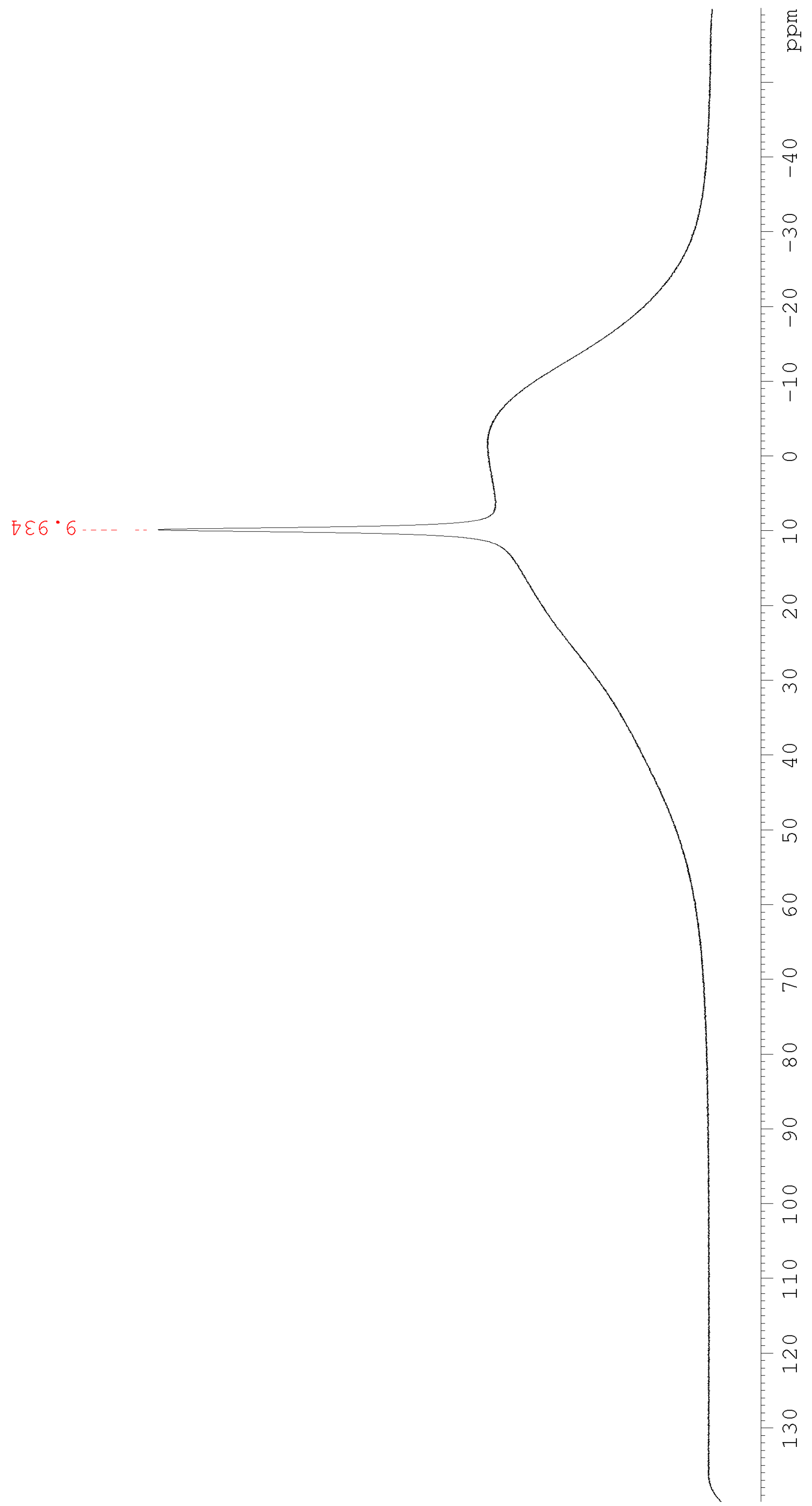

Figure S6. $^{11}B\{^{1}H\}$ NMR spectrum of **(Me)HC=C(Me)BCat(I*i*Pr)** in C_6D_6.

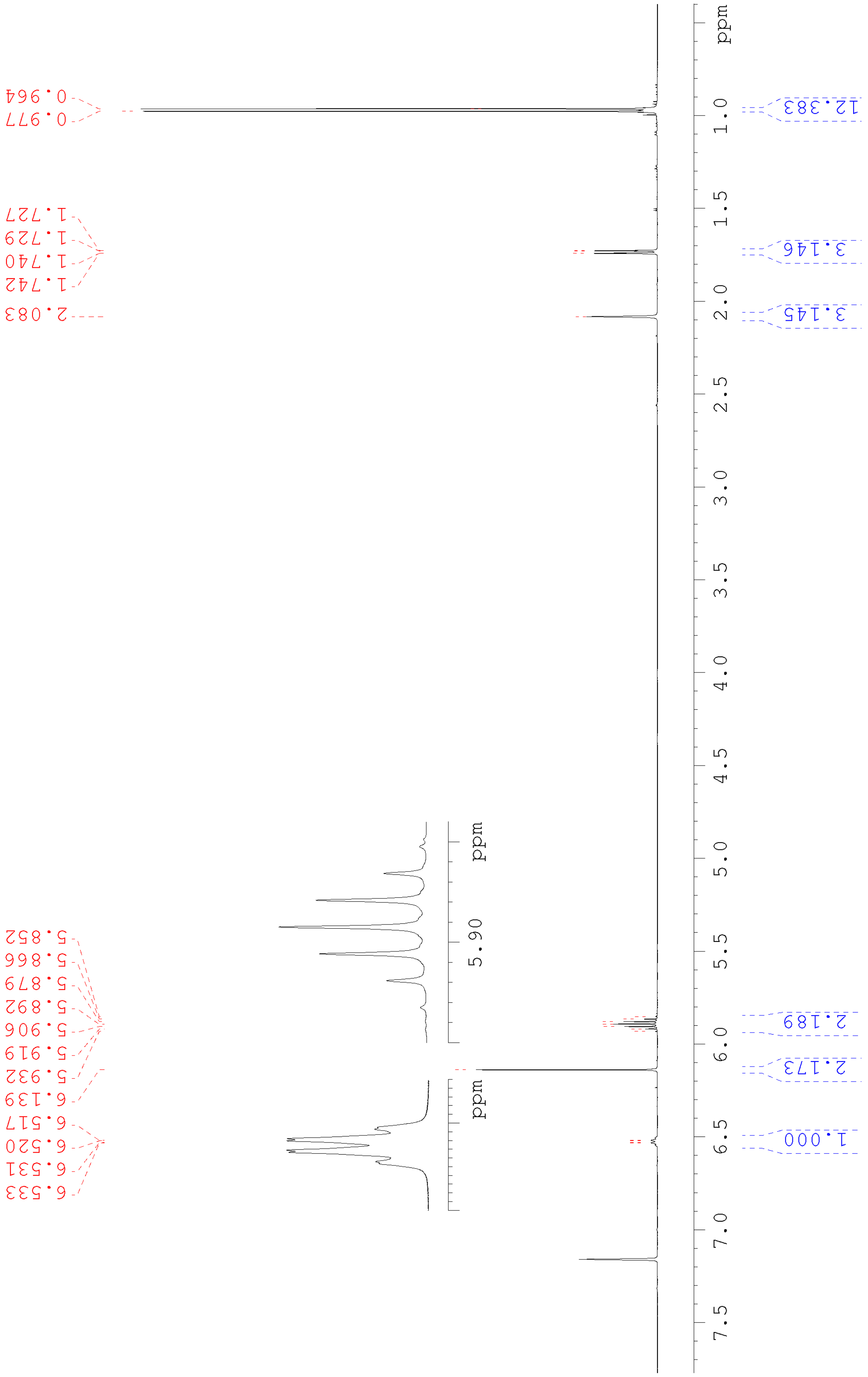

Figure S7. ^{1}H NMR spectrum of **1-Me** in C_6D_6.

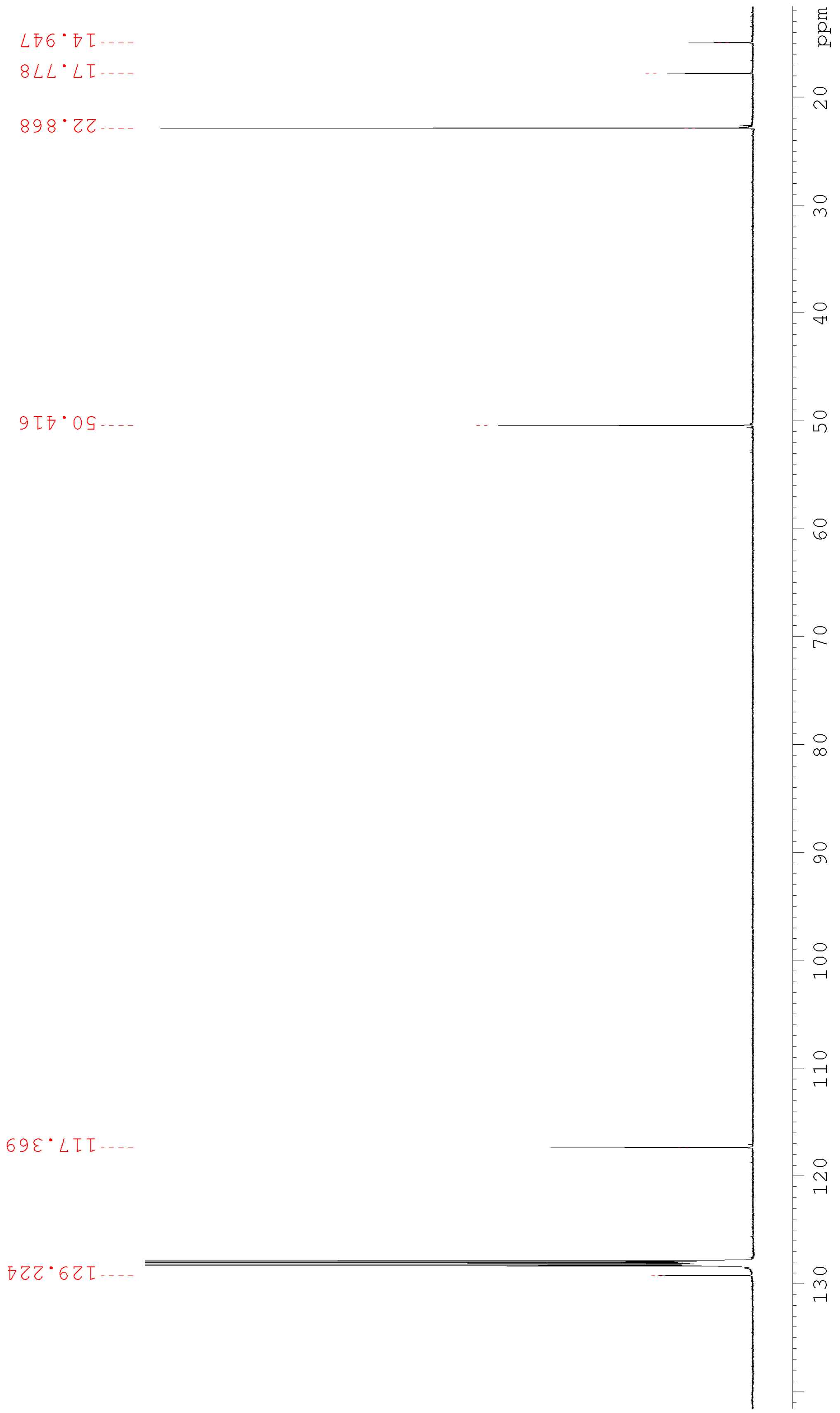

Figure S8. $^{13}C\{^{1}H\}$ NMR spectrum of **1-Me** in C_6D_6.

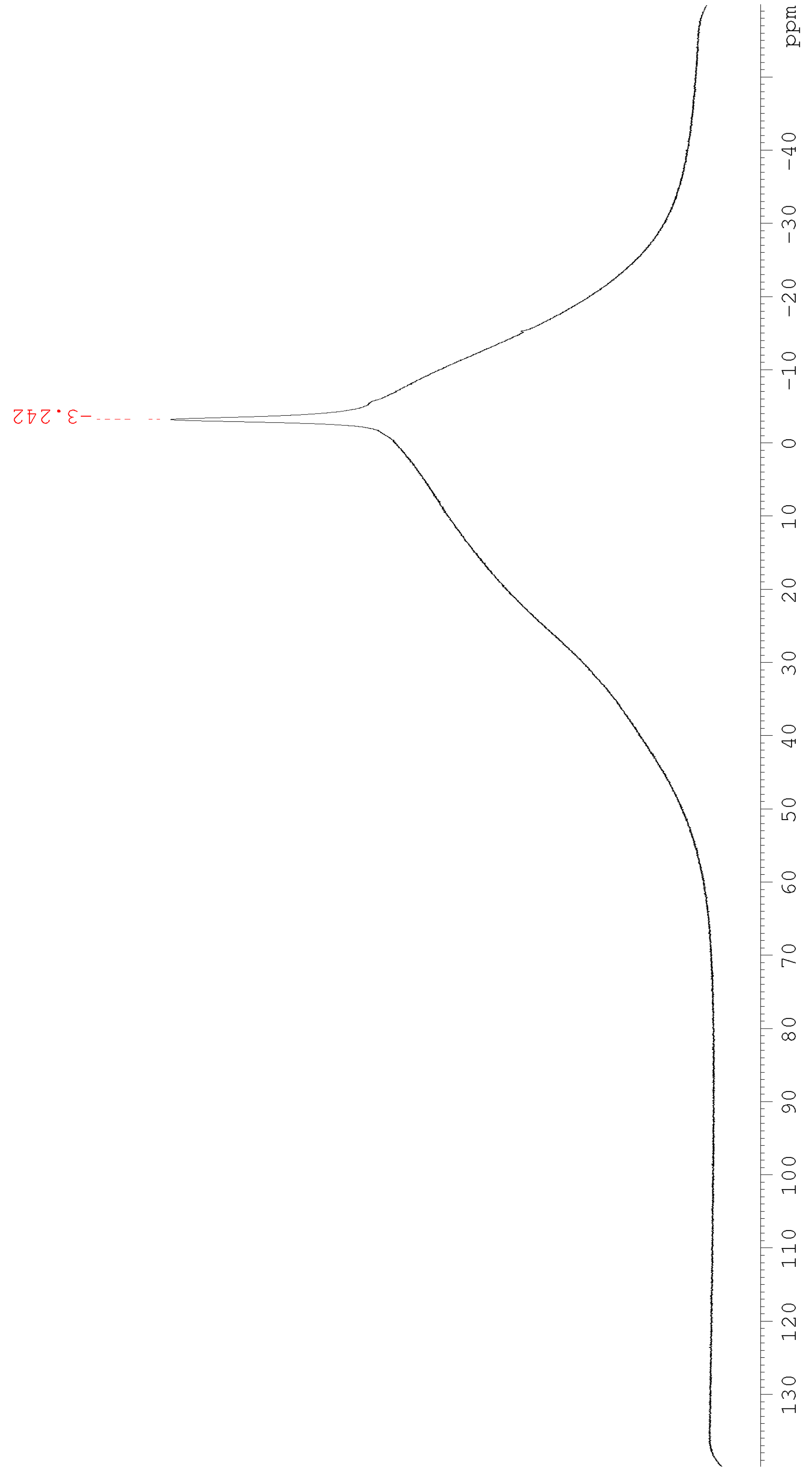

Figure S9. $^{11}B\{^{1}H\}$ NMR spectrum of **1-Me** in C_6D_6.

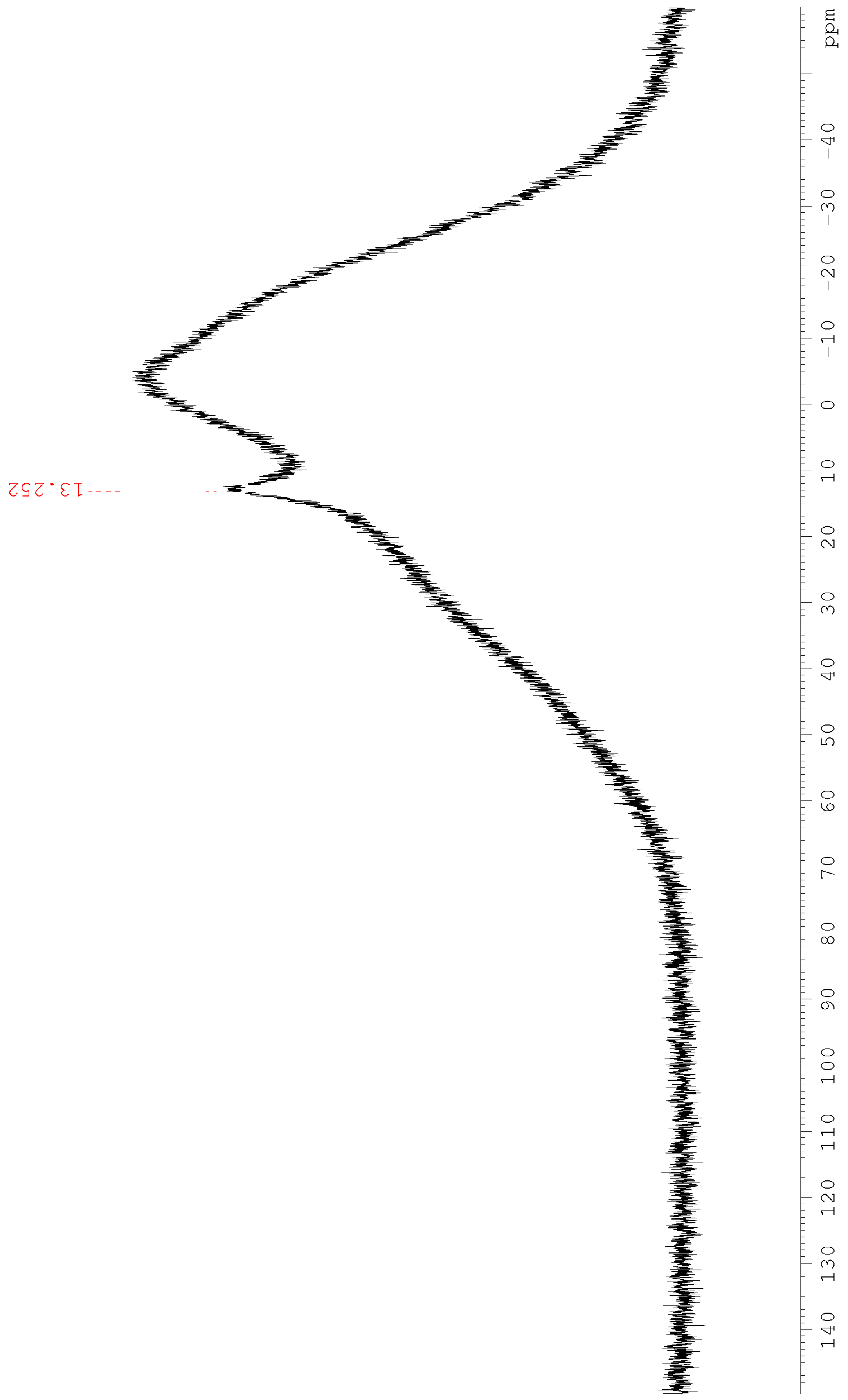

Figure S10. $^{11}B\{^{1}H\}$ NMR spectrum of **2-Me** in C_6H_6.

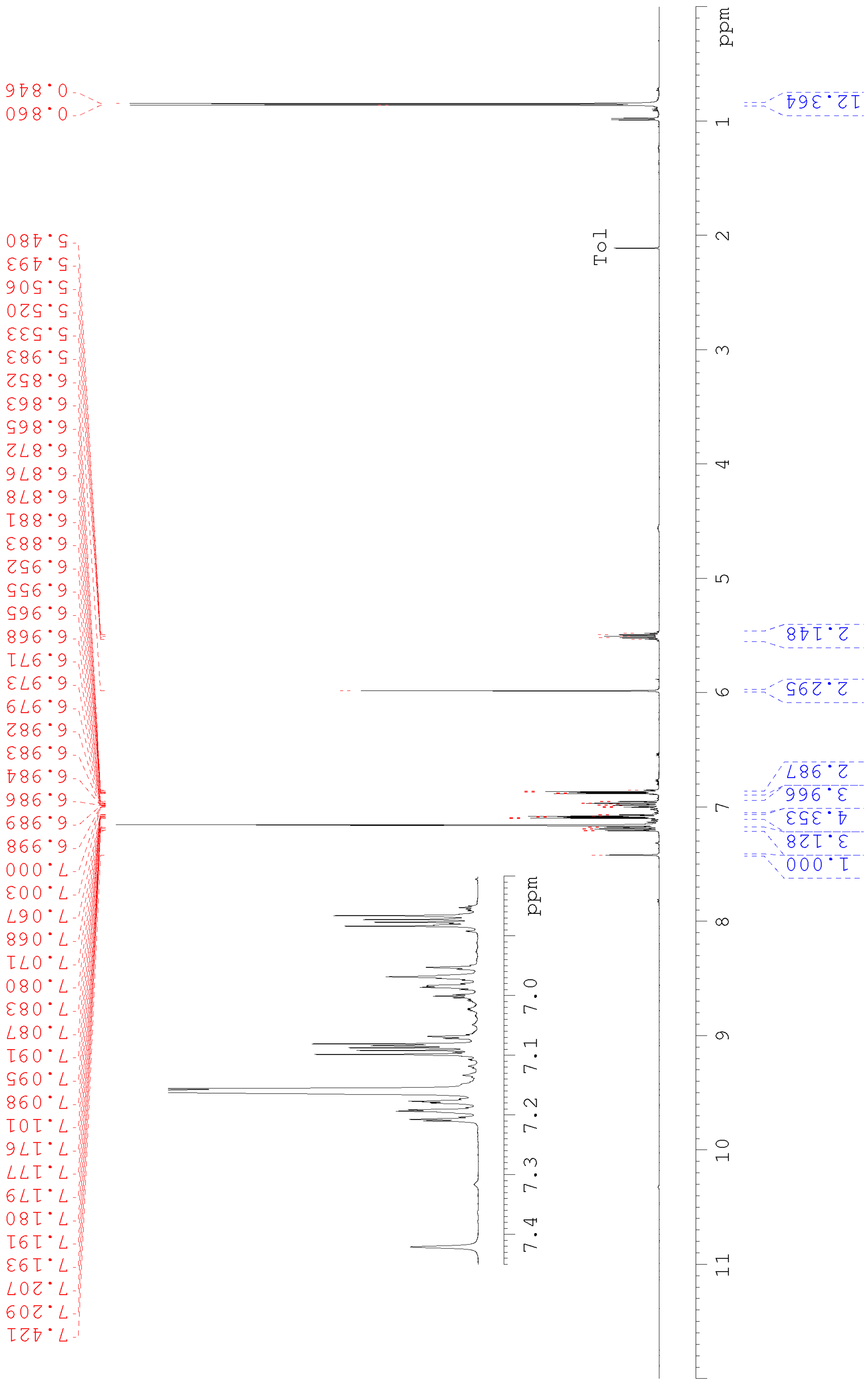

Figure S11. ^{1}H NMR spectrum of **(Ph)HC=C(Ph)BCat(I*i*Pr)** in C_6D_6.

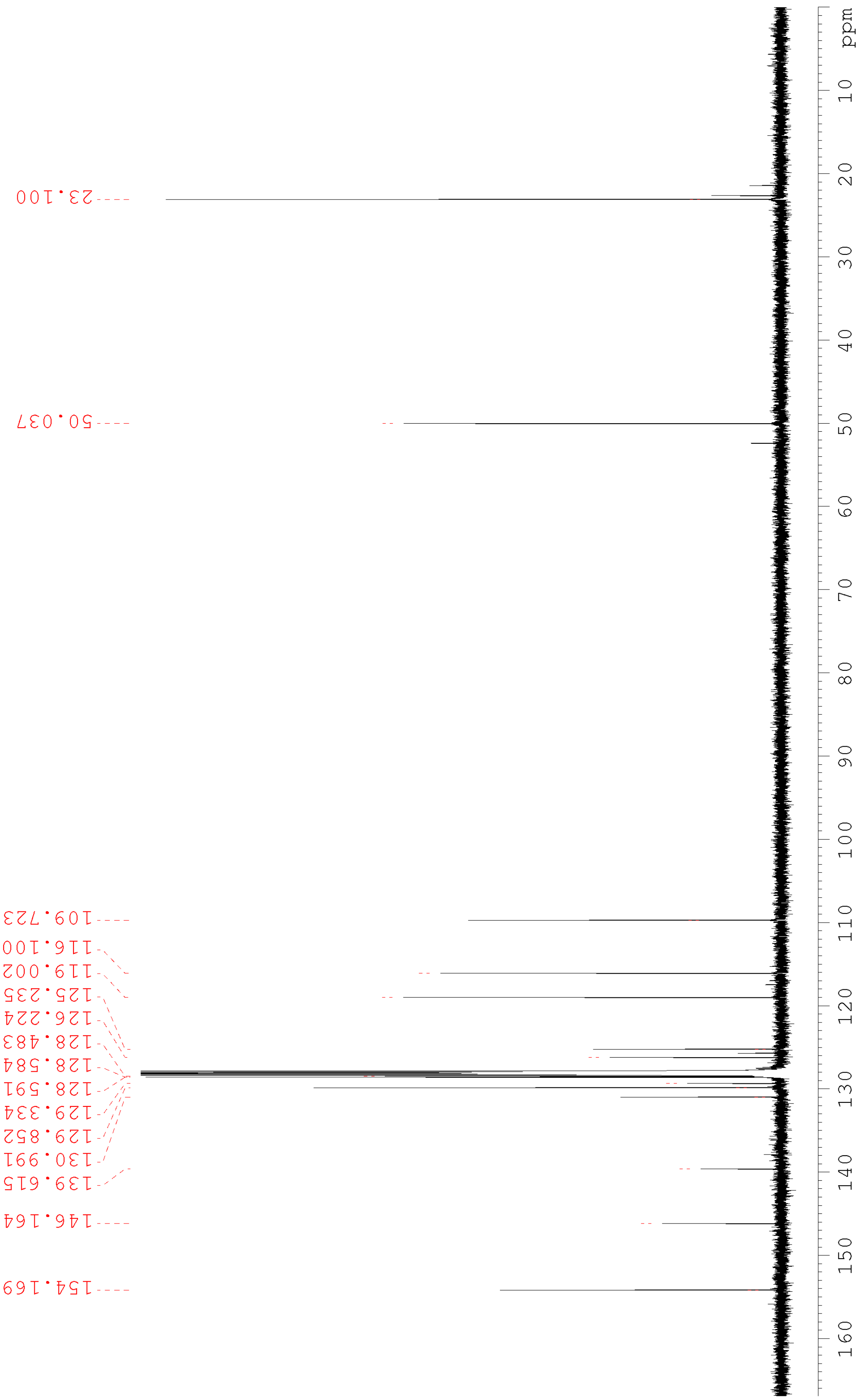

Figure S12. $^{13}C\{^{1}H\}$ NMR spectrum of **(Ph)HC=C(Ph)BCat(I*i*Pr)** in C_6D_6.

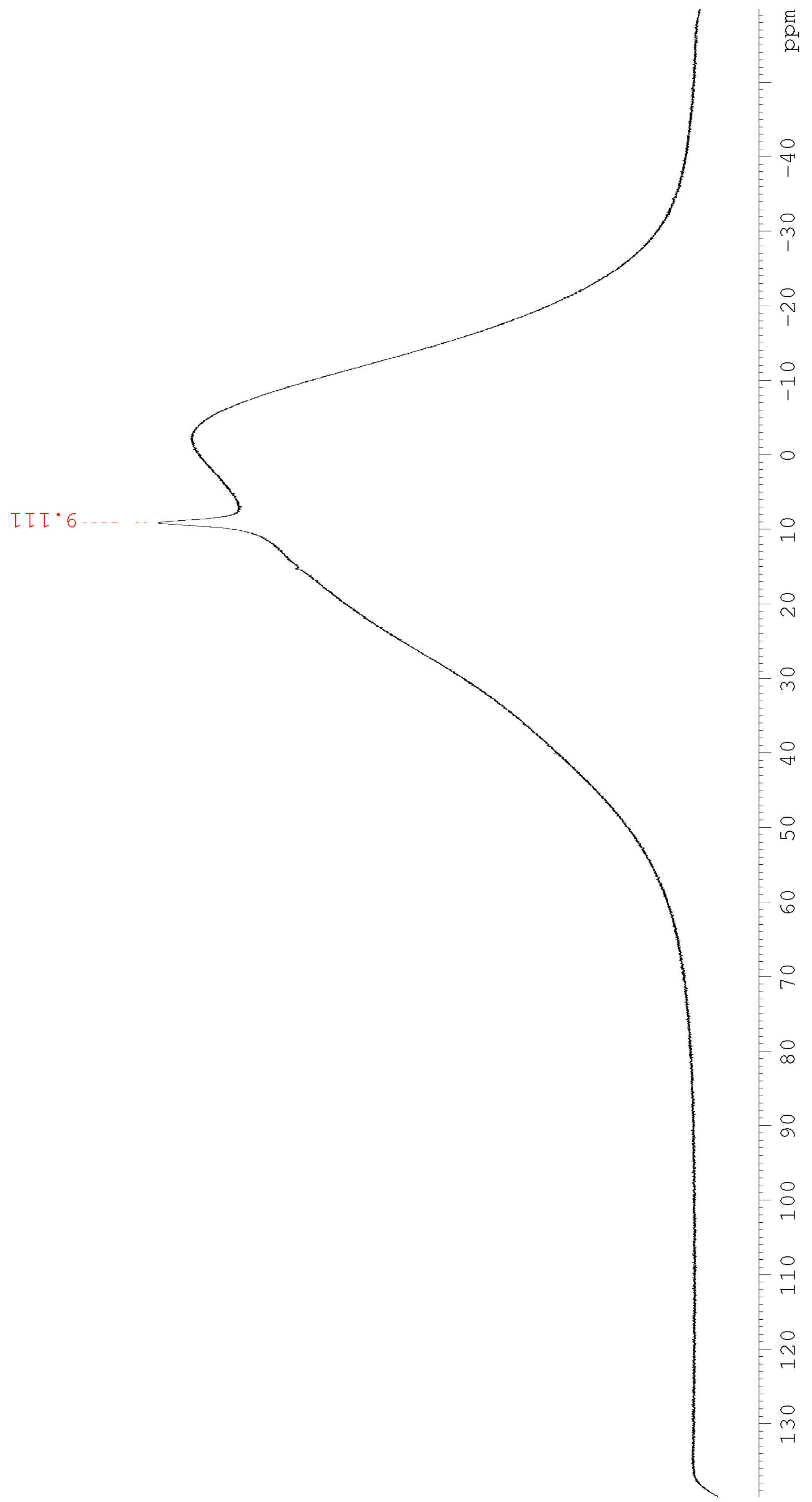

Figure S13. $^{11}B\{^{1}H\}$ NMR spectrum of **(Ph)HC=C(Ph)BCat(I*i*Pr)** in C_6D_6.

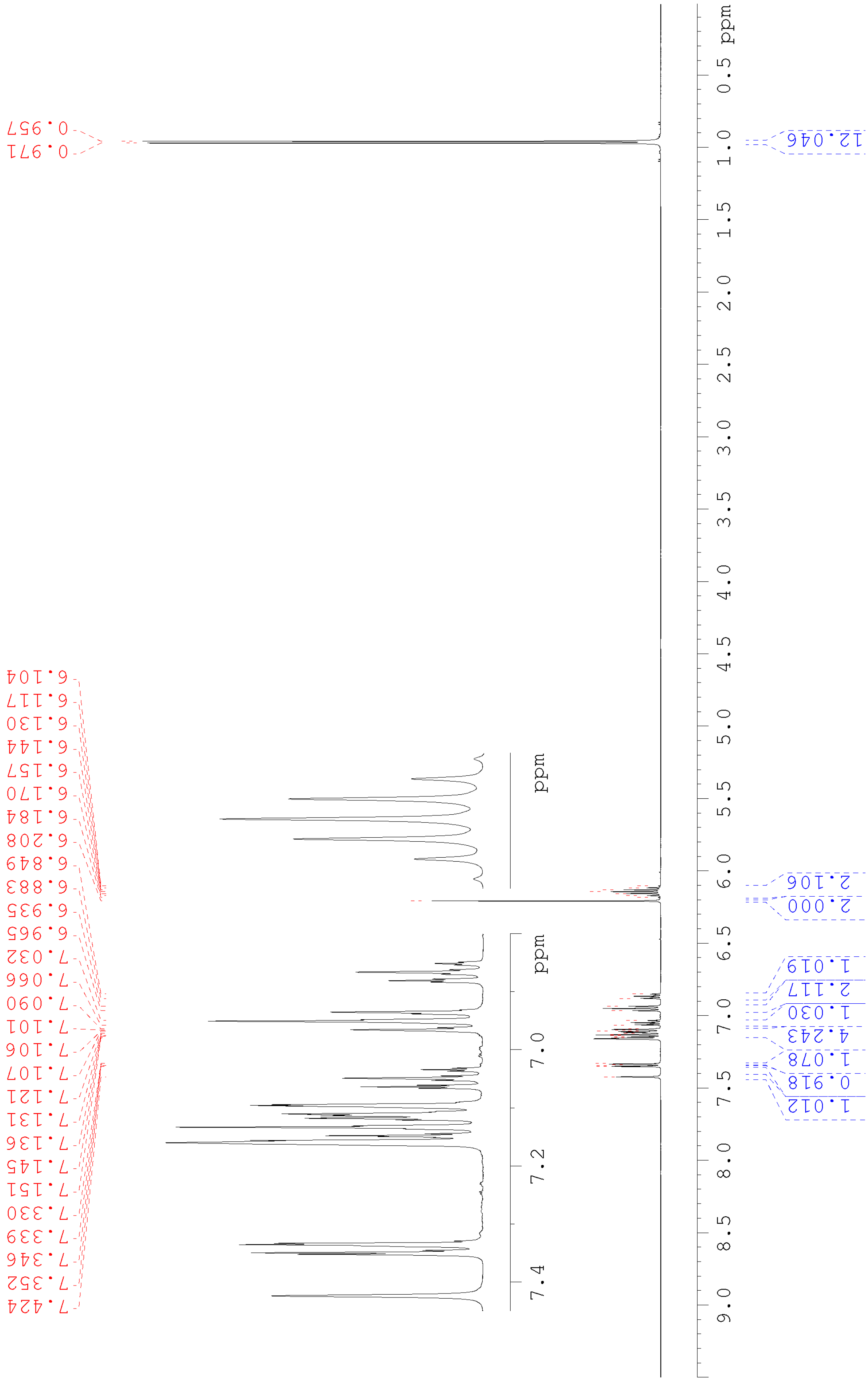

Figure S14. ^{1}H NMR spectrum of **1-Ph** in C_6D_6.

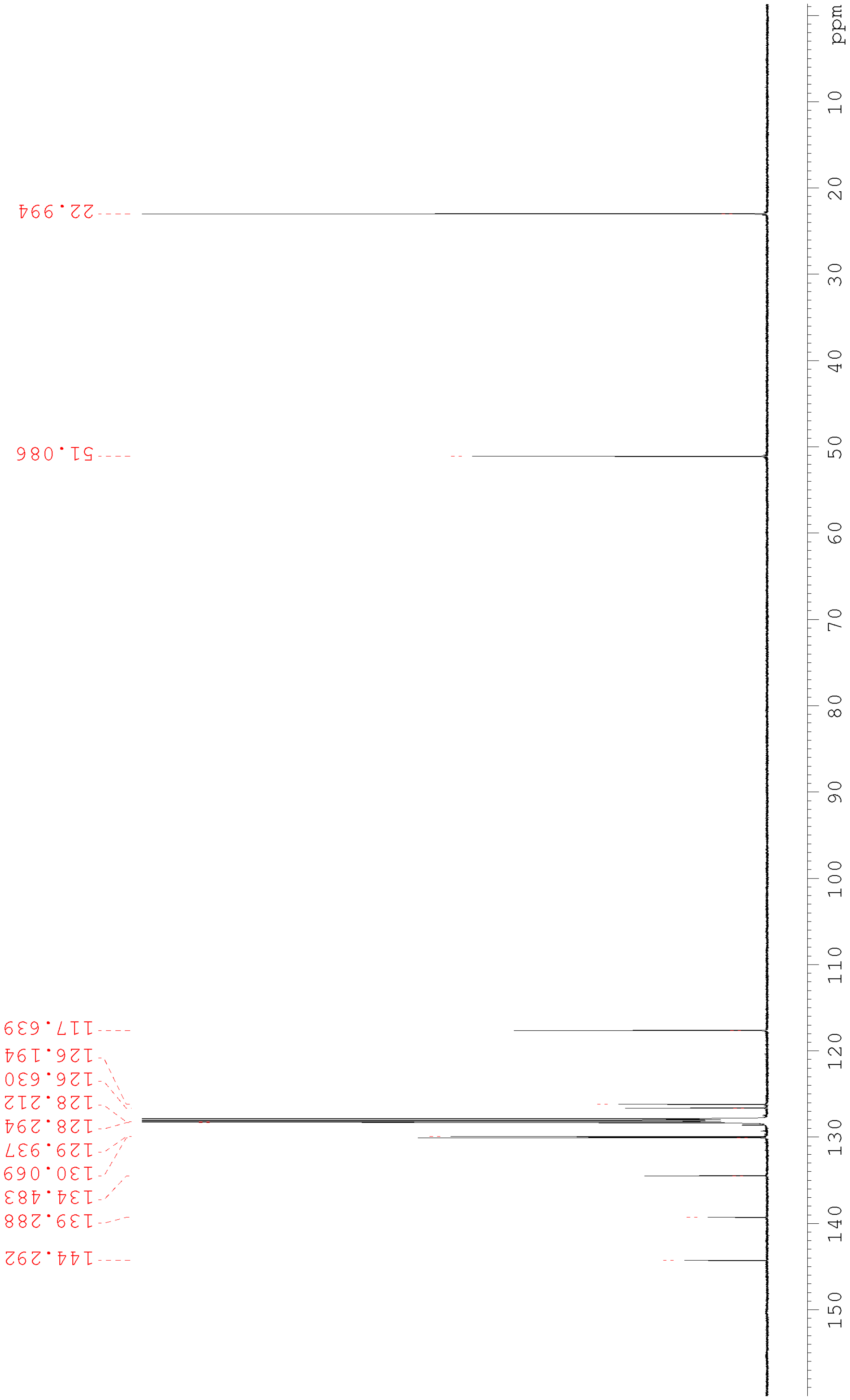

Figure S15. $^{13}C\{^{1}H\}$ NMR spectrum of **1-Ph** in C_6D_6.

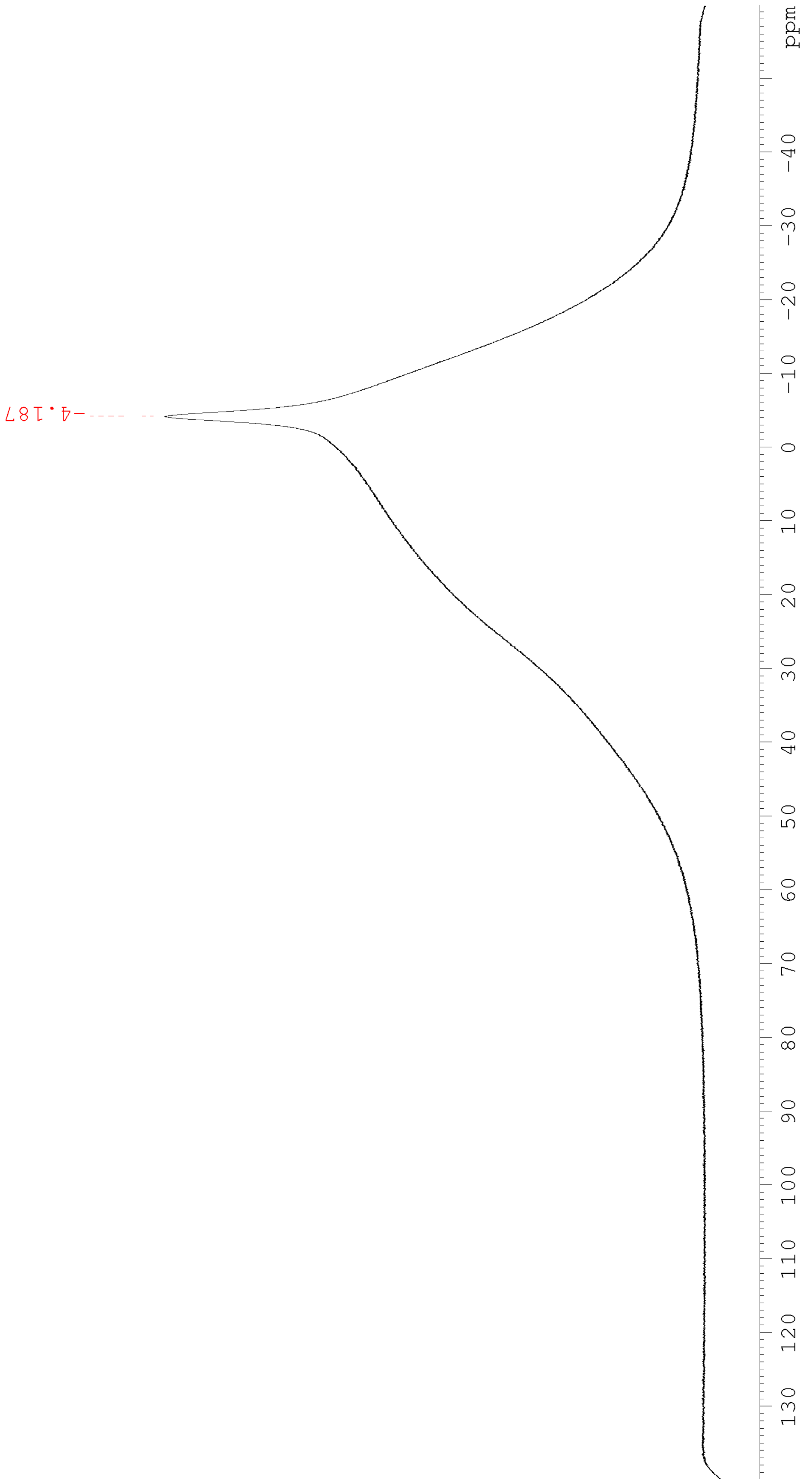

Figure S16. $^{11}B\{^{1}H\}$ NMR spectrum of **1-Ph** in C_6D_6.

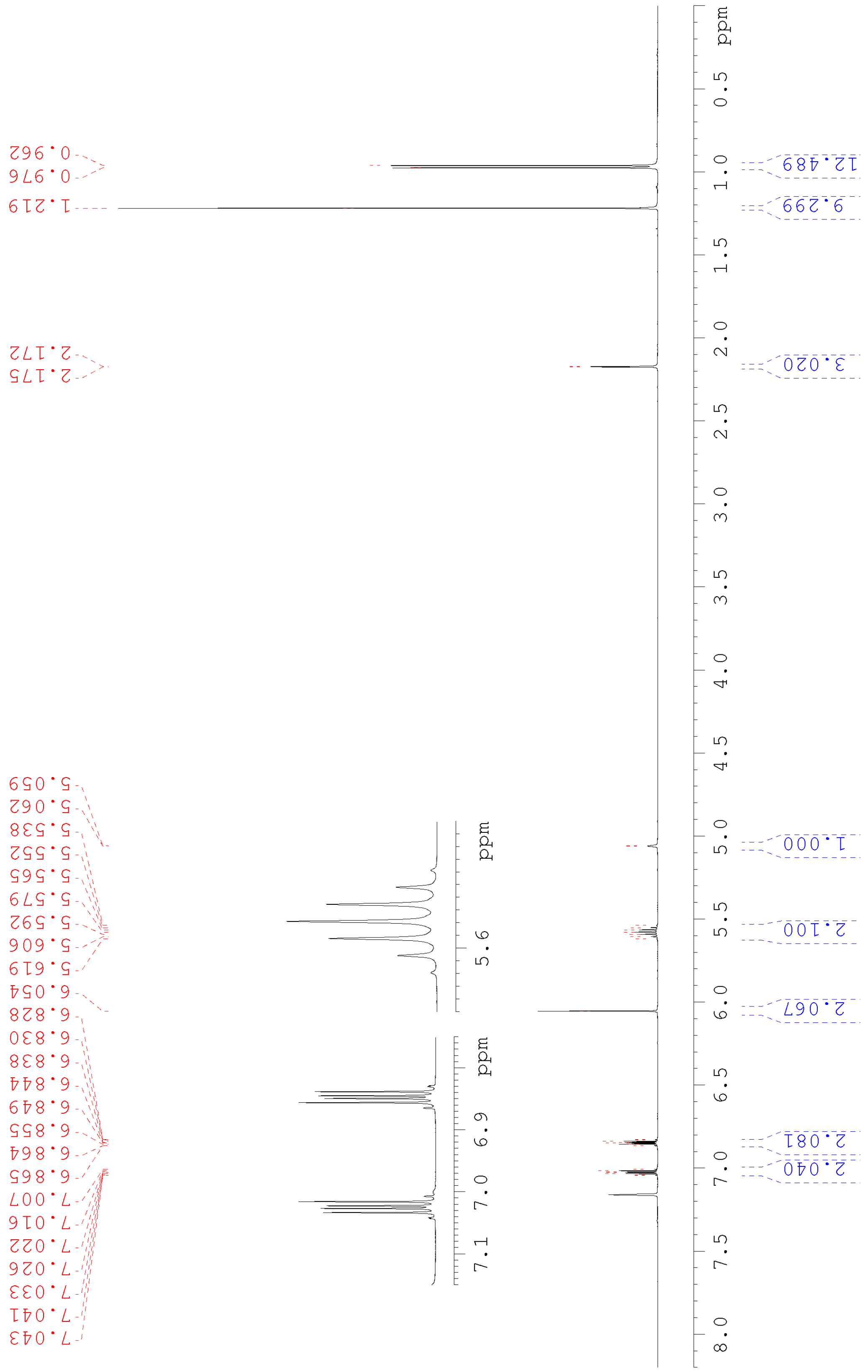

Figure S17. ^{1}H NMR spectrum of **(*t*Bu)HC=C(Me)BCat(I*i*Pr)** in C_6D_6.

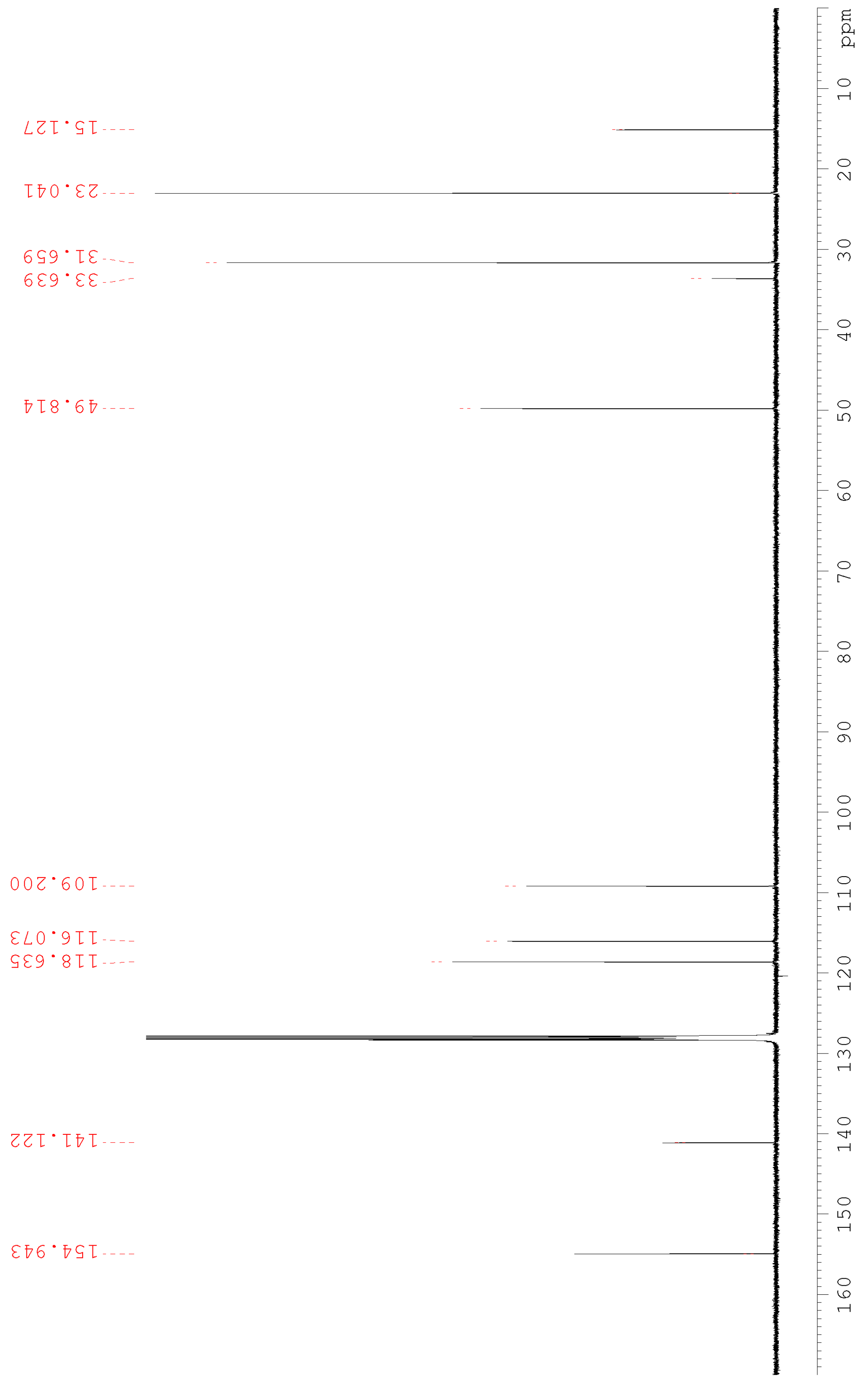

Figure S18. $^{13}C\{^1H\}$ NMR spectrum of **(*t*Bu)HC=C(Me)BCat(I*i*Pr)** in C_6D_6.

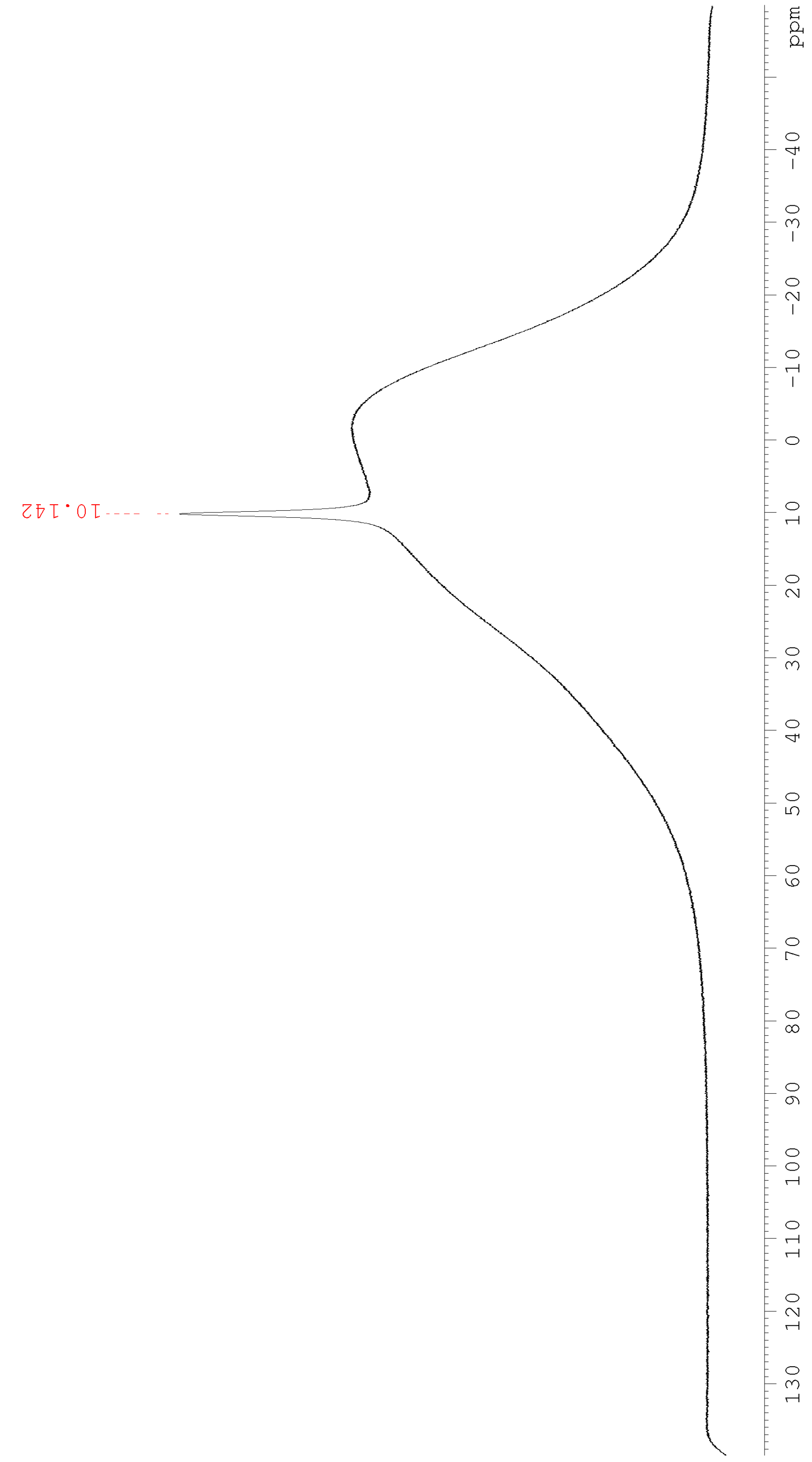

Figure S19. $^{11}B\{^{1}H\}$ NMR spectrum of **(*t*Bu)HC=C(Me)BCat(I*i*Pr)** in C_6D_6.

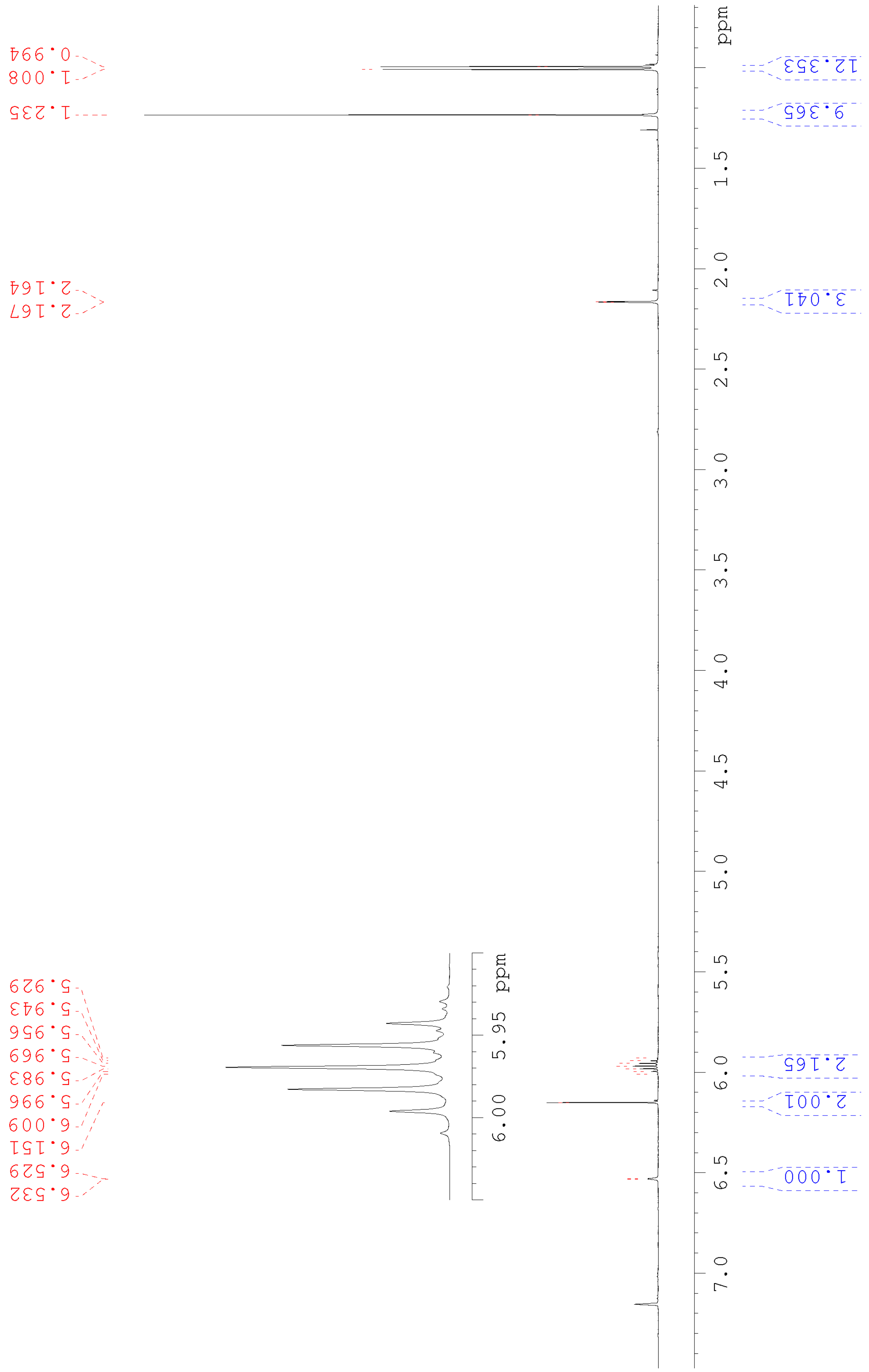

Figure S20. ^{1}H NMR spectrum of **1-*t*Bu** in C_6D_6.

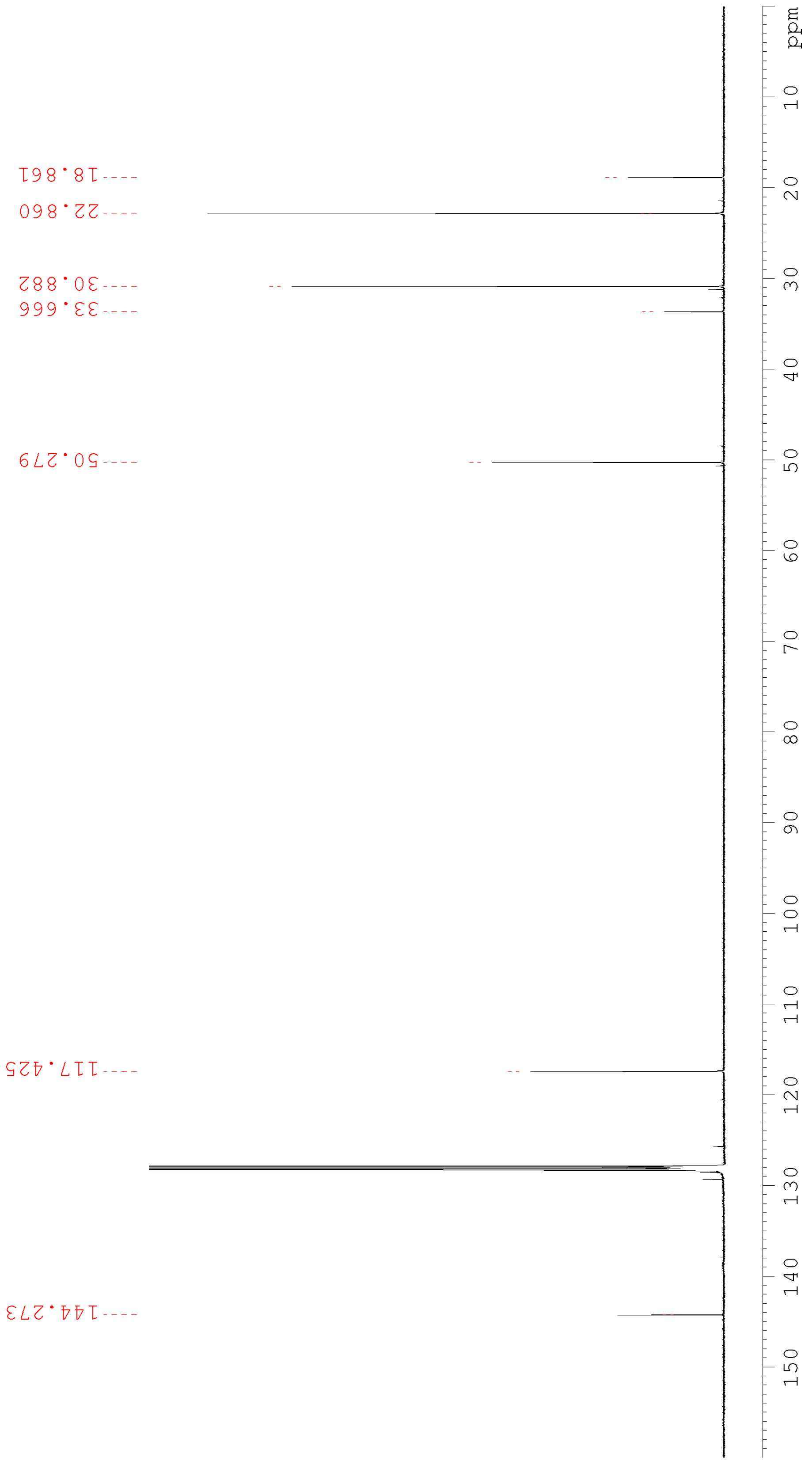

Figure S21. $^{13}C\{^{1}H\}$ NMR spectrum of **1-*t*Bu** in C_6D_6.

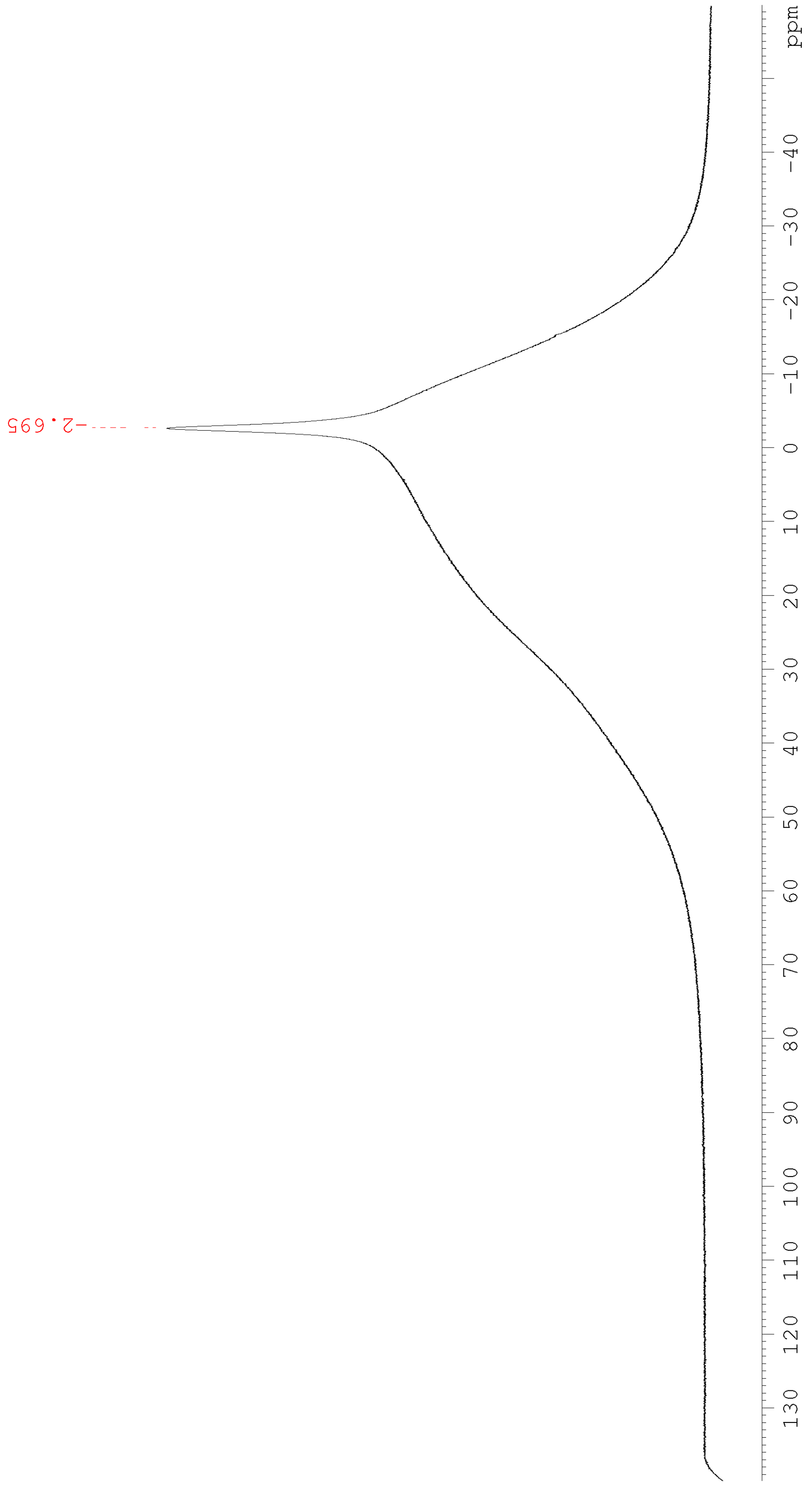

Figure S22. $^{11}B\{^{1}H\}$ NMR spectrum of **1-*t*Bu** in C_6D_6.

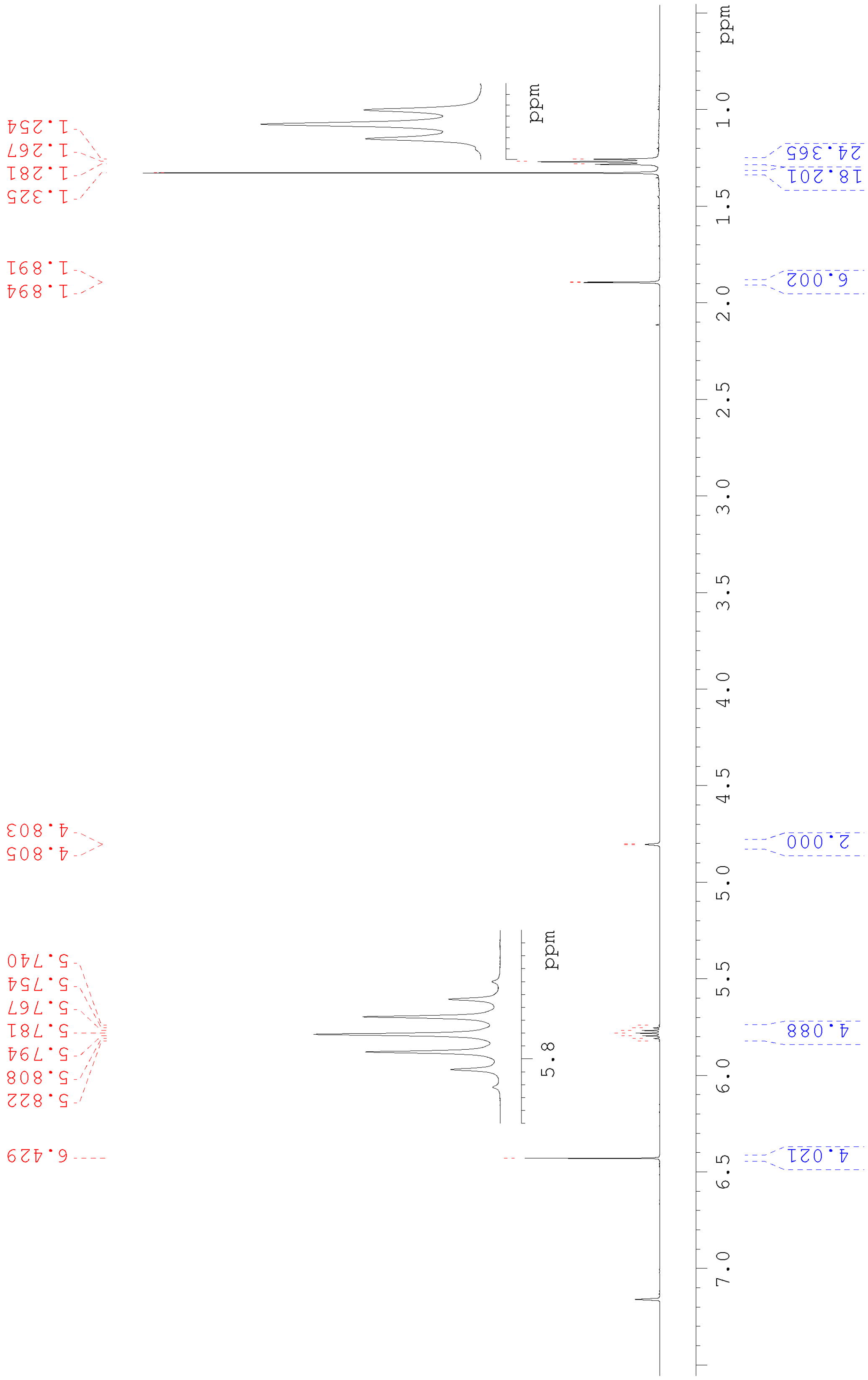

Figure S23. ^{1}H NMR spectrum of **4** in C_6D_6.

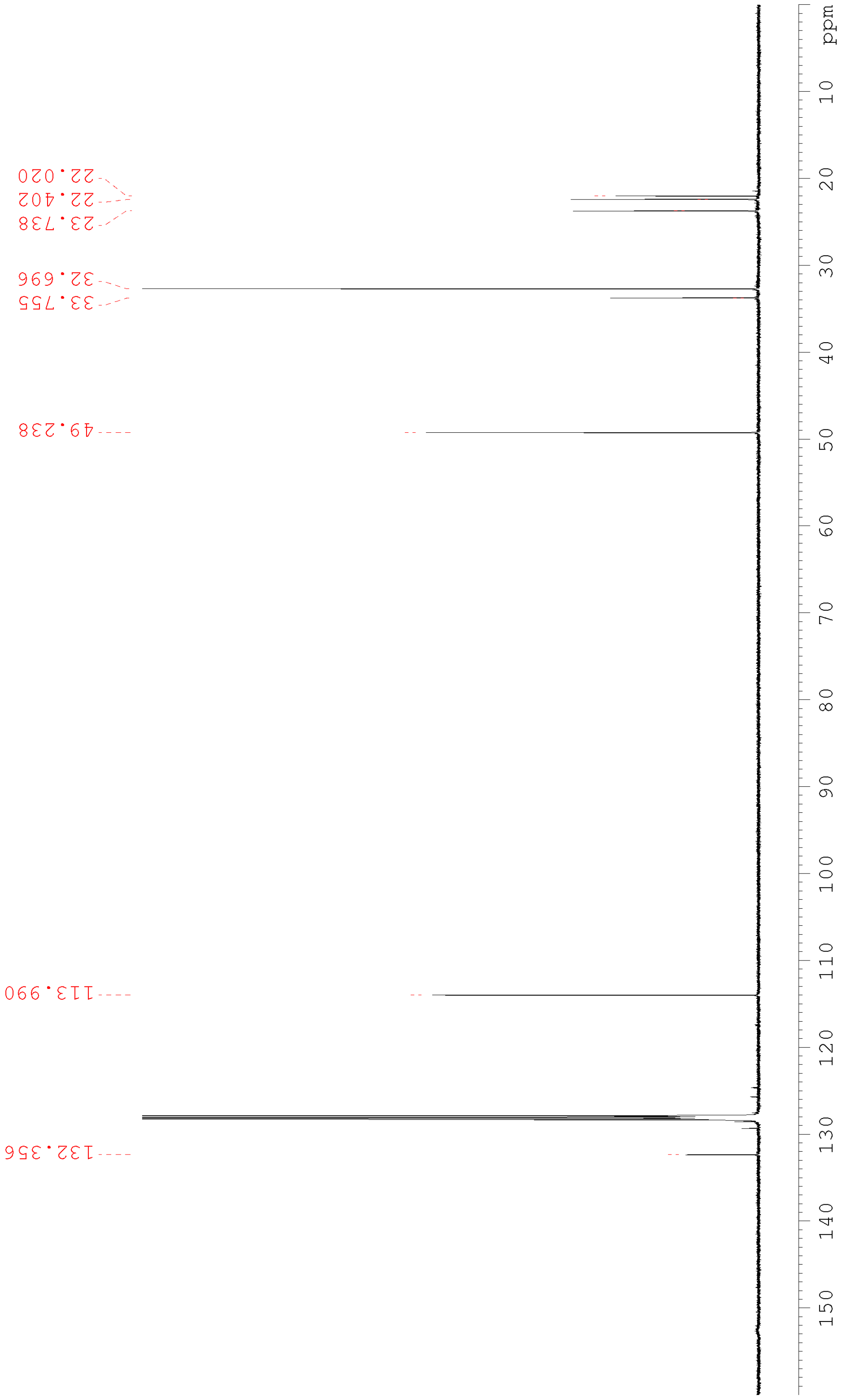

Figure S24. $^{13}C\{^{1}H\}$ NMR spectrum of **4** in C_6D_6.

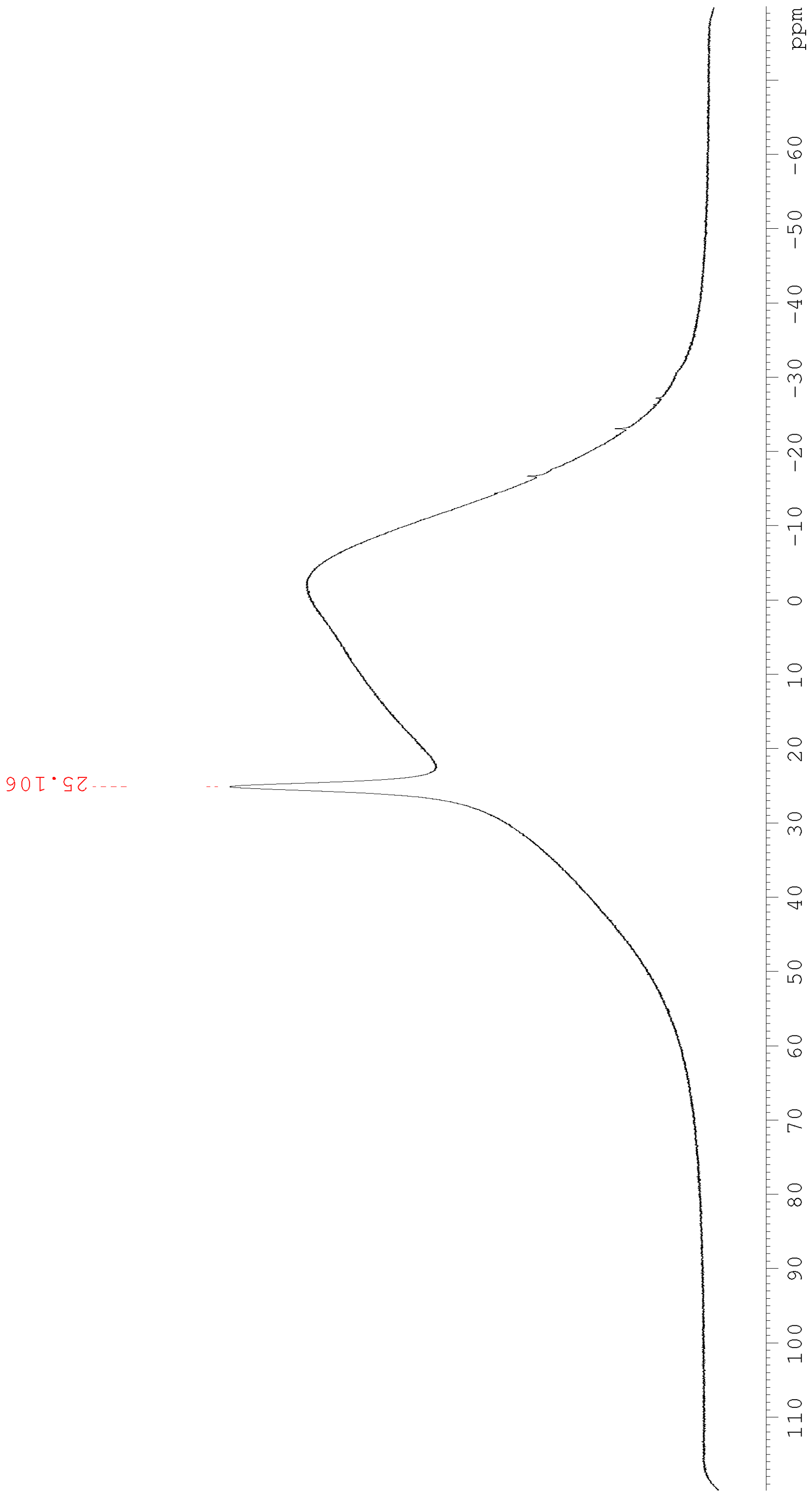

Figure S25. $^{11}B\{^{1}H\}$ NMR spectrum of **4** in C_6D_6.

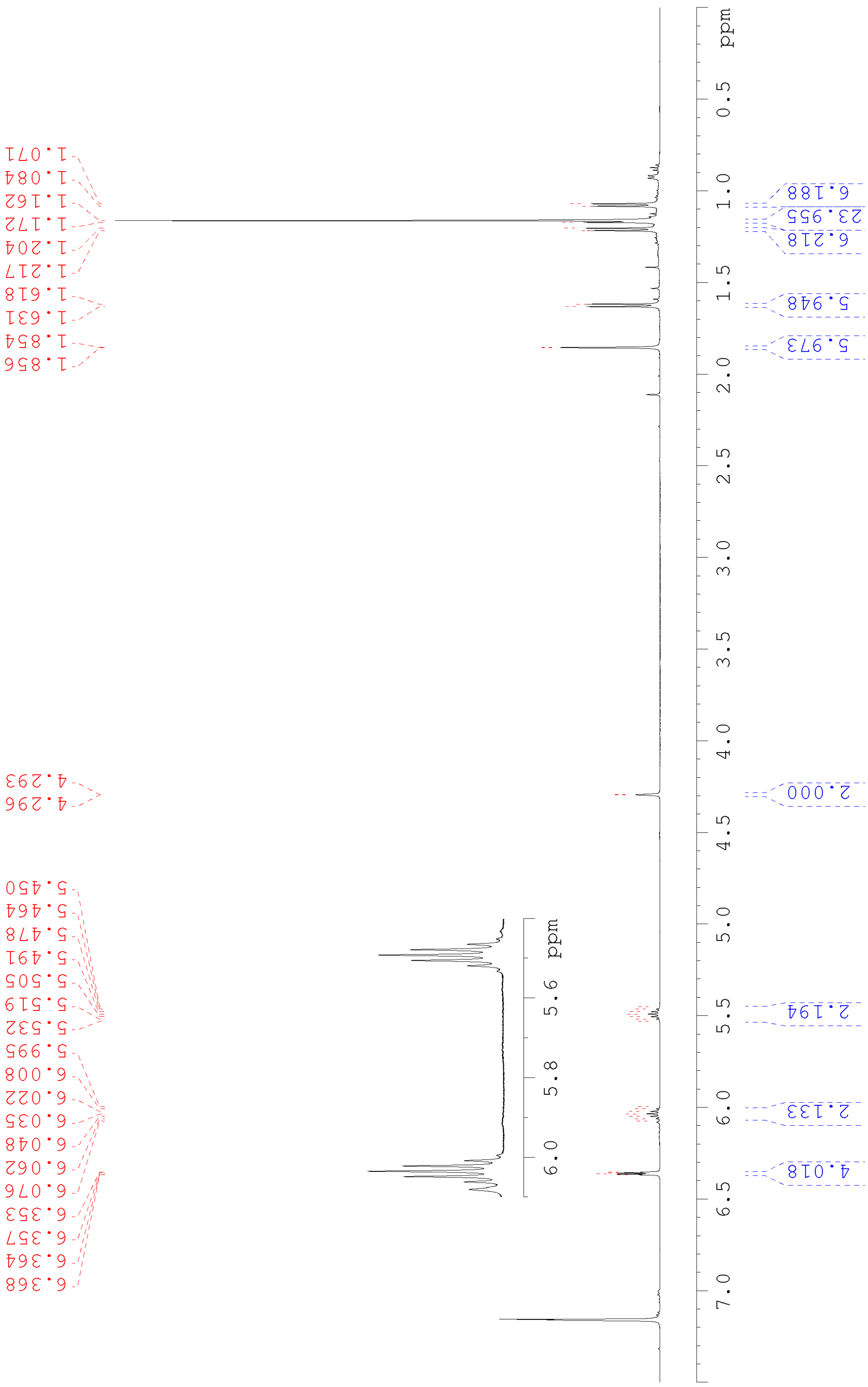

Figure S26. ^{1}H NMR spectrum of **4-Cu** in C_6D_6.

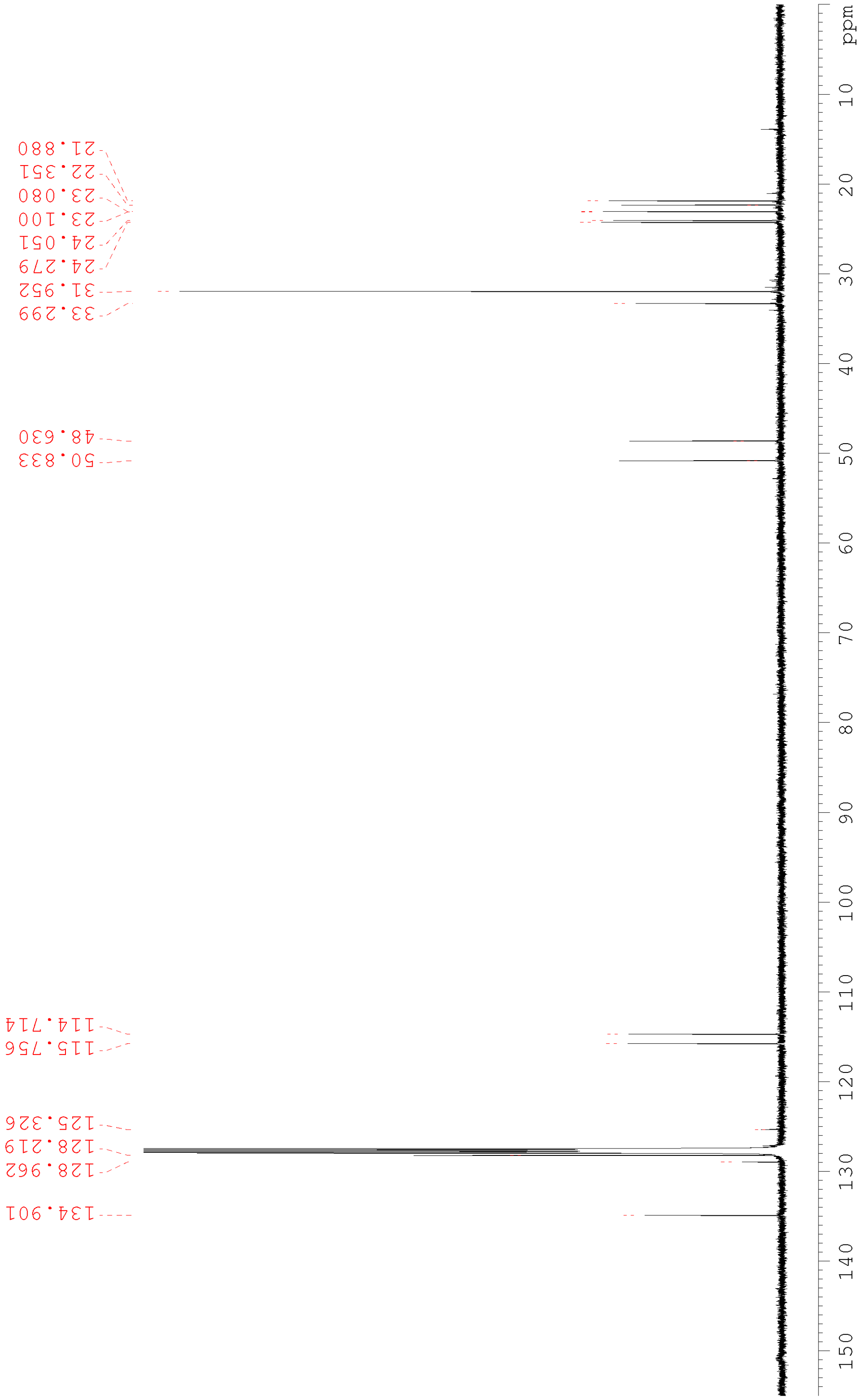

Figure S27. $^{13}C\{^{1}H\}$ NMR spectrum of **4-Cu** in C_6D_6.

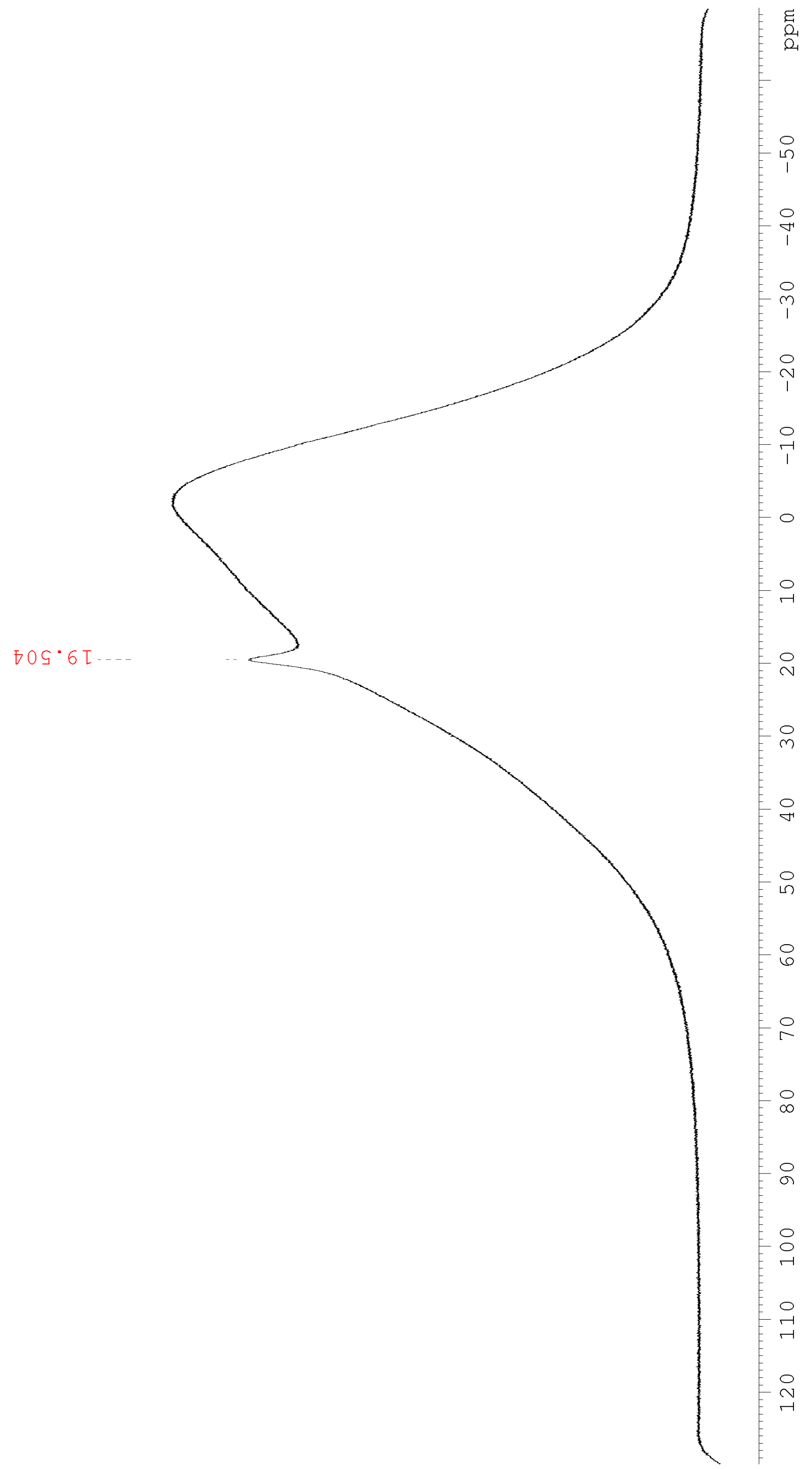

Figure S28. $^{11}B\{^{1}H\}$ NMR spectrum of **4-Cu** in C_6D_6.

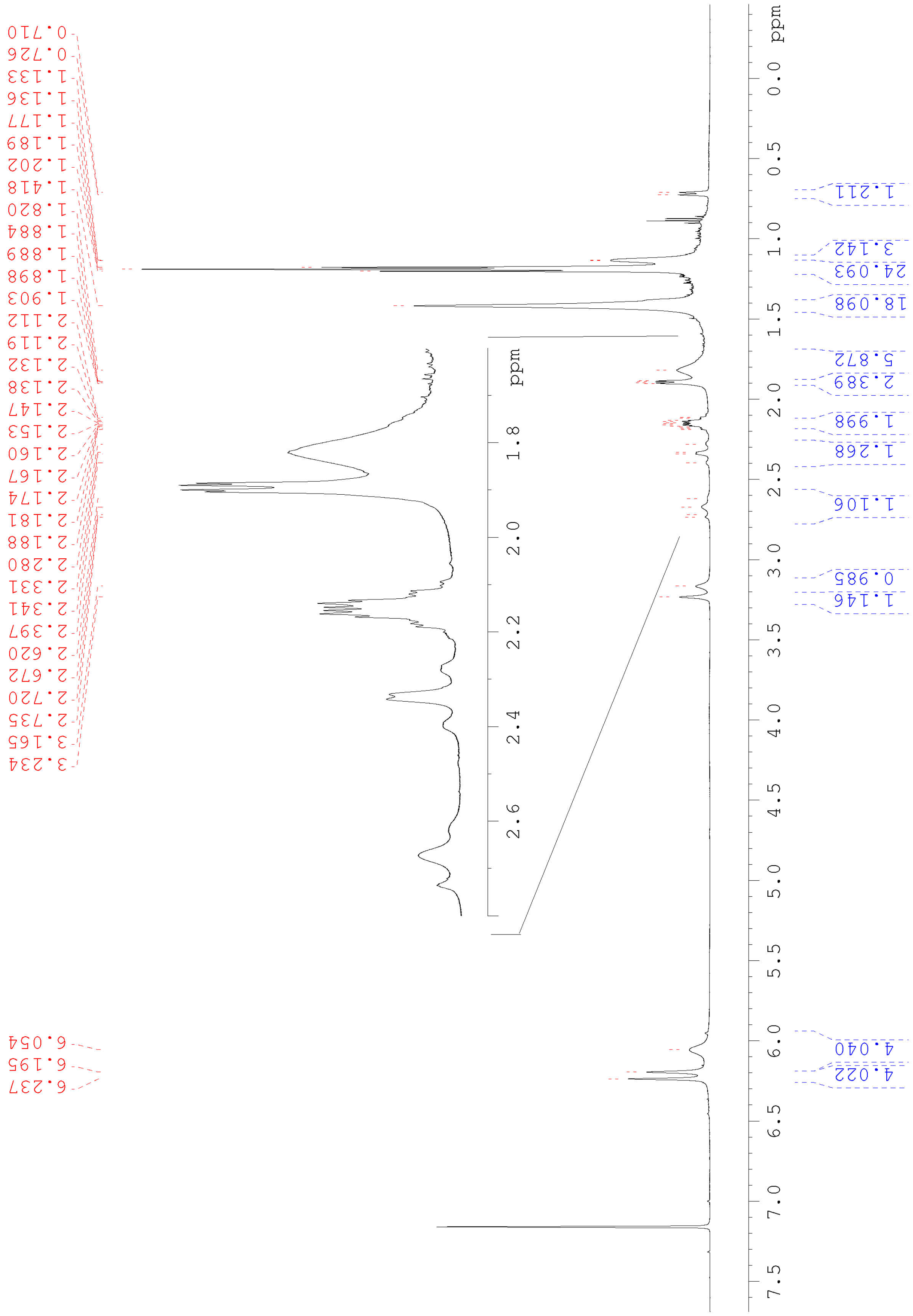

Figure S29. ^{1}H NMR spectrum of **4-Pt** in C_6D_6 at rt.

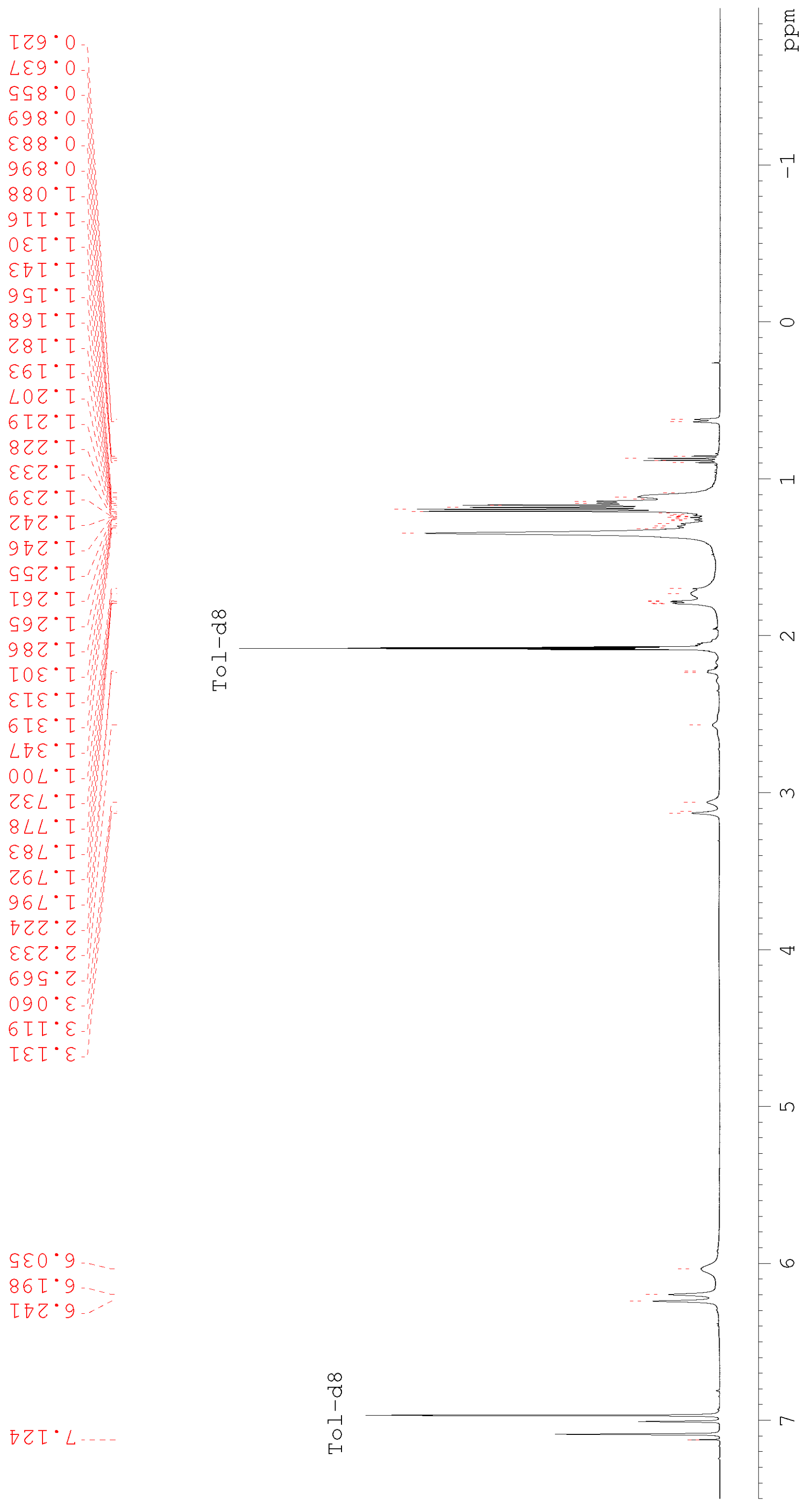

Figure S30. ^{1}H NMR spectrum of **4-Pt** in d_8-tol at rt.

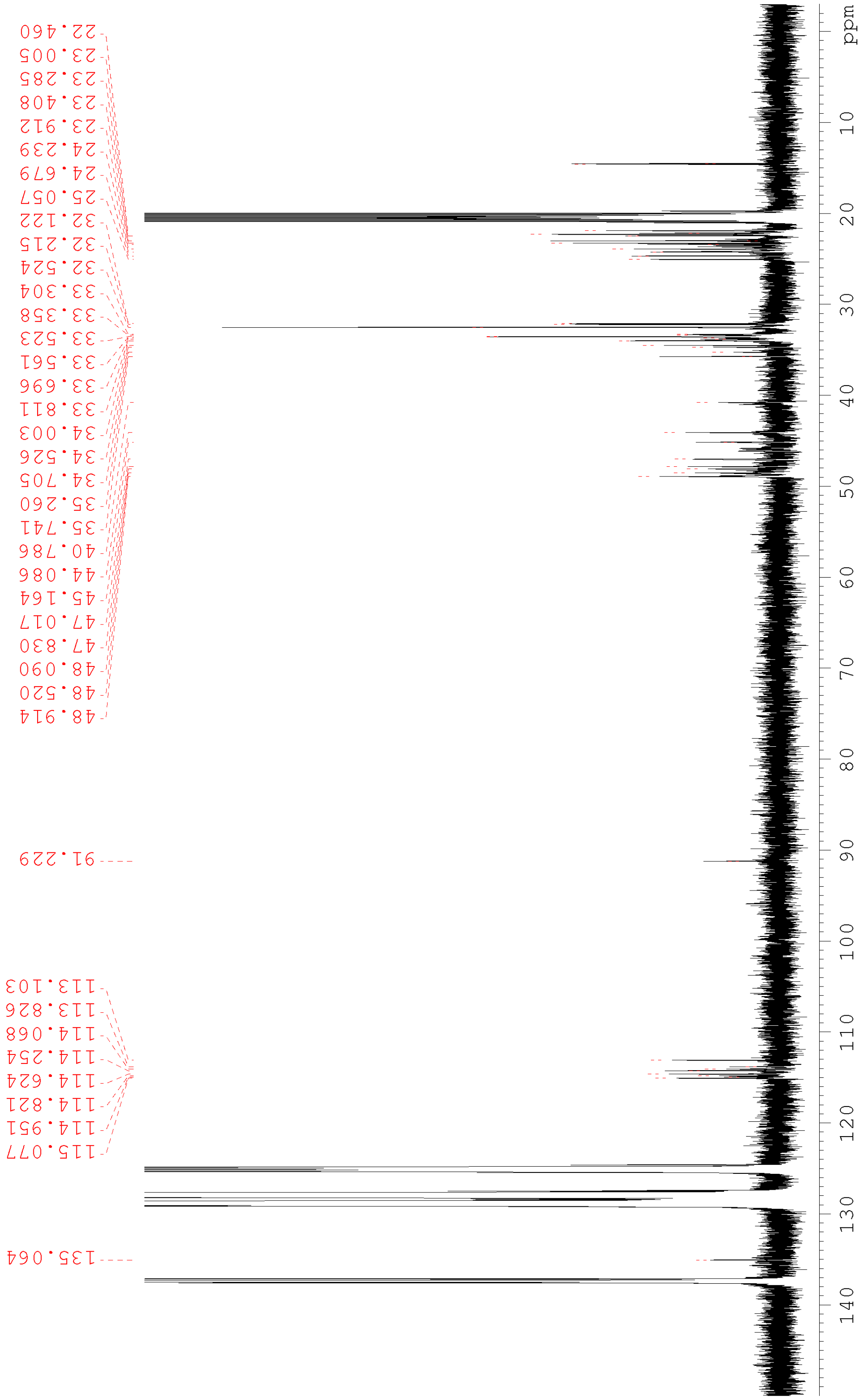

Figure S31. $^{13}C\{^{1}H\}$ NMR spectrum of **4-Pt** in d_8-tol at –40 °C.

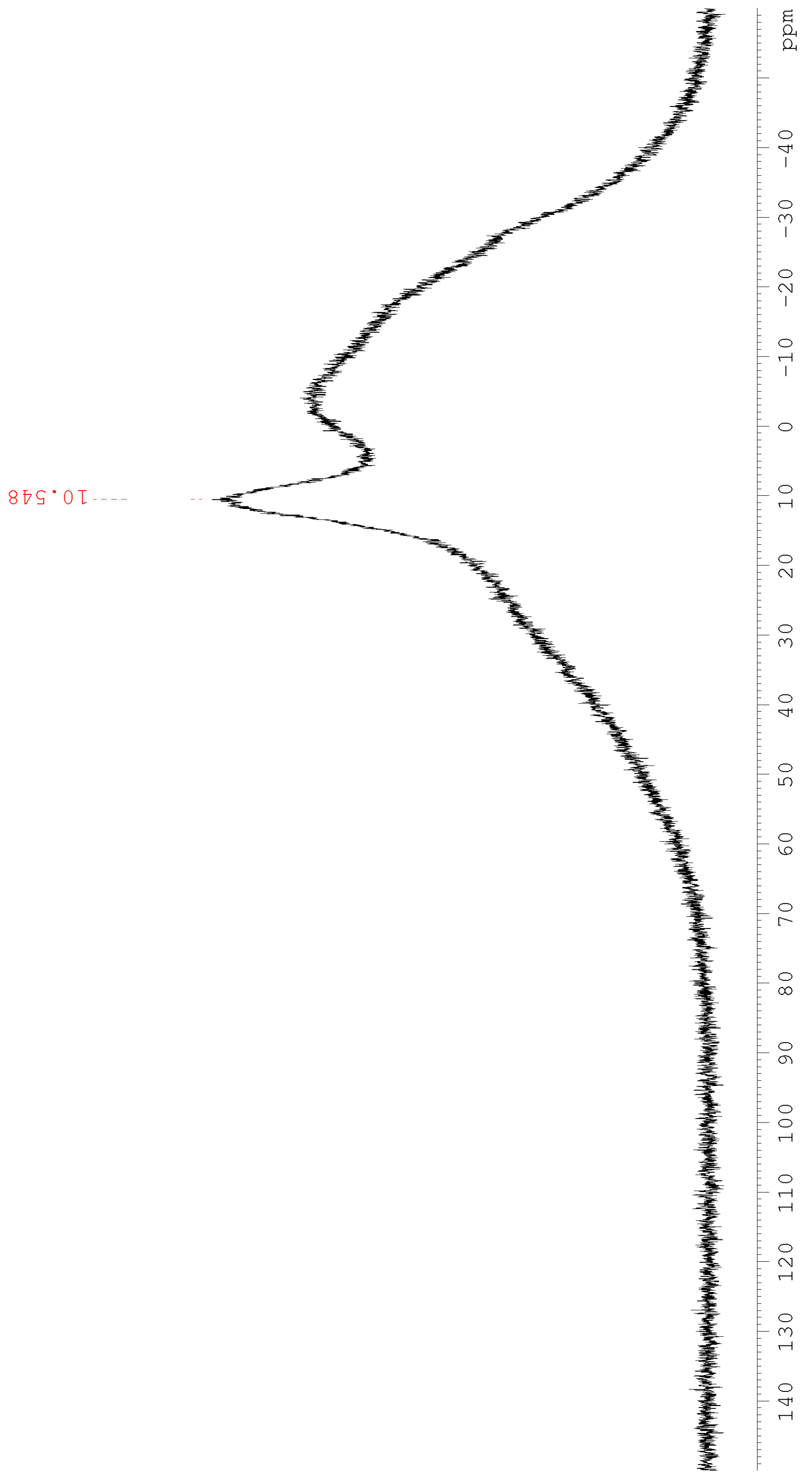

Figure S32. $^{11}B\{^{1}H\}$ NMR spectrum of **4-Pt** in C_6D_6.

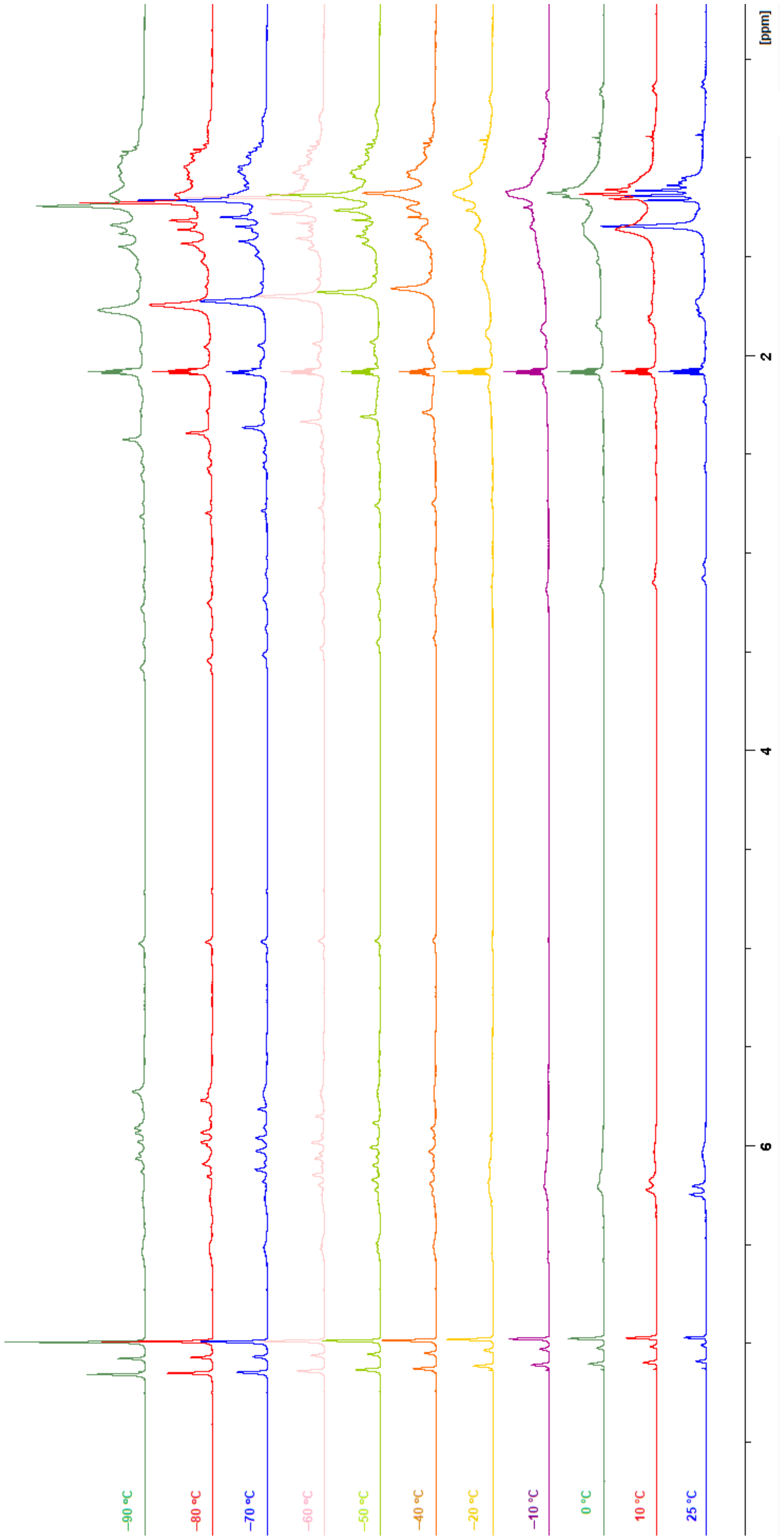

Figure S33. Stack-plot of ^{1}H NMR spectra of **4-Pt** in d_8-tol from 25 °C to –90 °C.

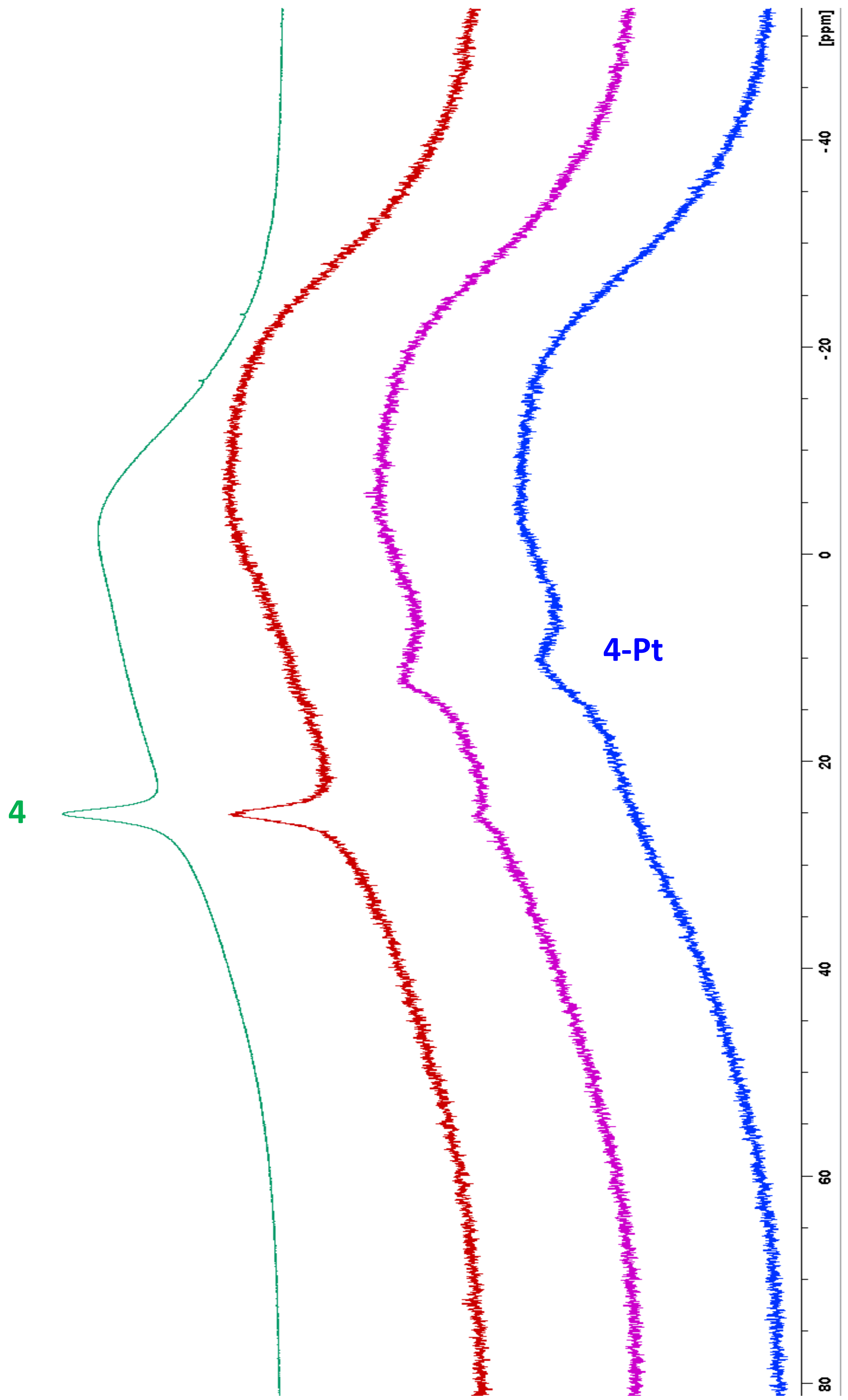

Figure S34. Stackplot of the ^{11}B NMR spectra of isolated **4-Pt** (blue), the rt reaction of **4-Pt** with 10 equiv. I*i*Pr directly after mixing (purple), after 18 h at rt (red) and of isolated **4** (green).

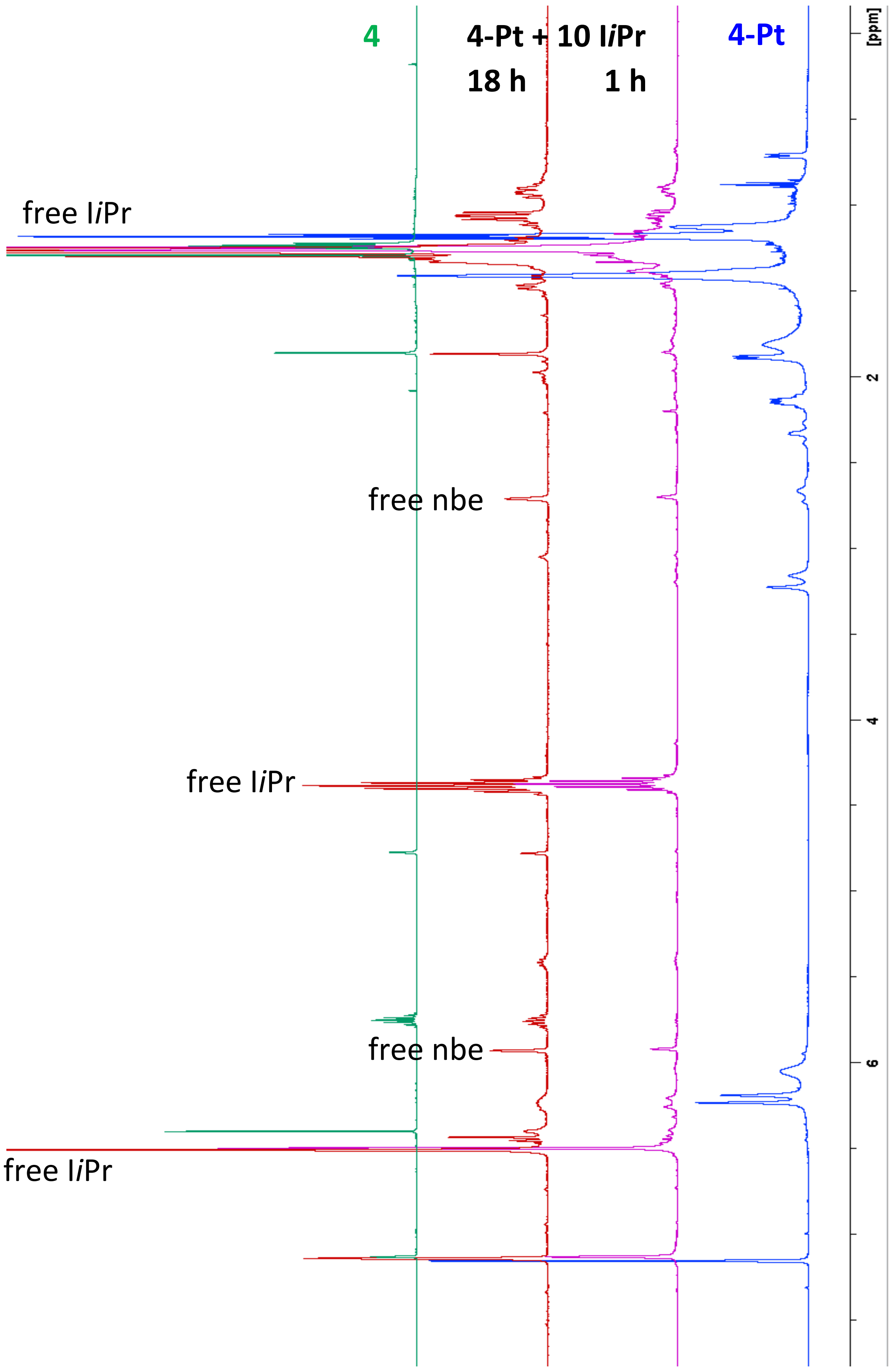

Figure S35. Stackplot of the ^{1}H NMR spectra of isolated **4-Pt** (blue), the rt reaction of **4-Pt** with 10 equiv. I*i*Pr directly after mixing (purple), after 18 h at rt (red) and of isolated **4** (green).

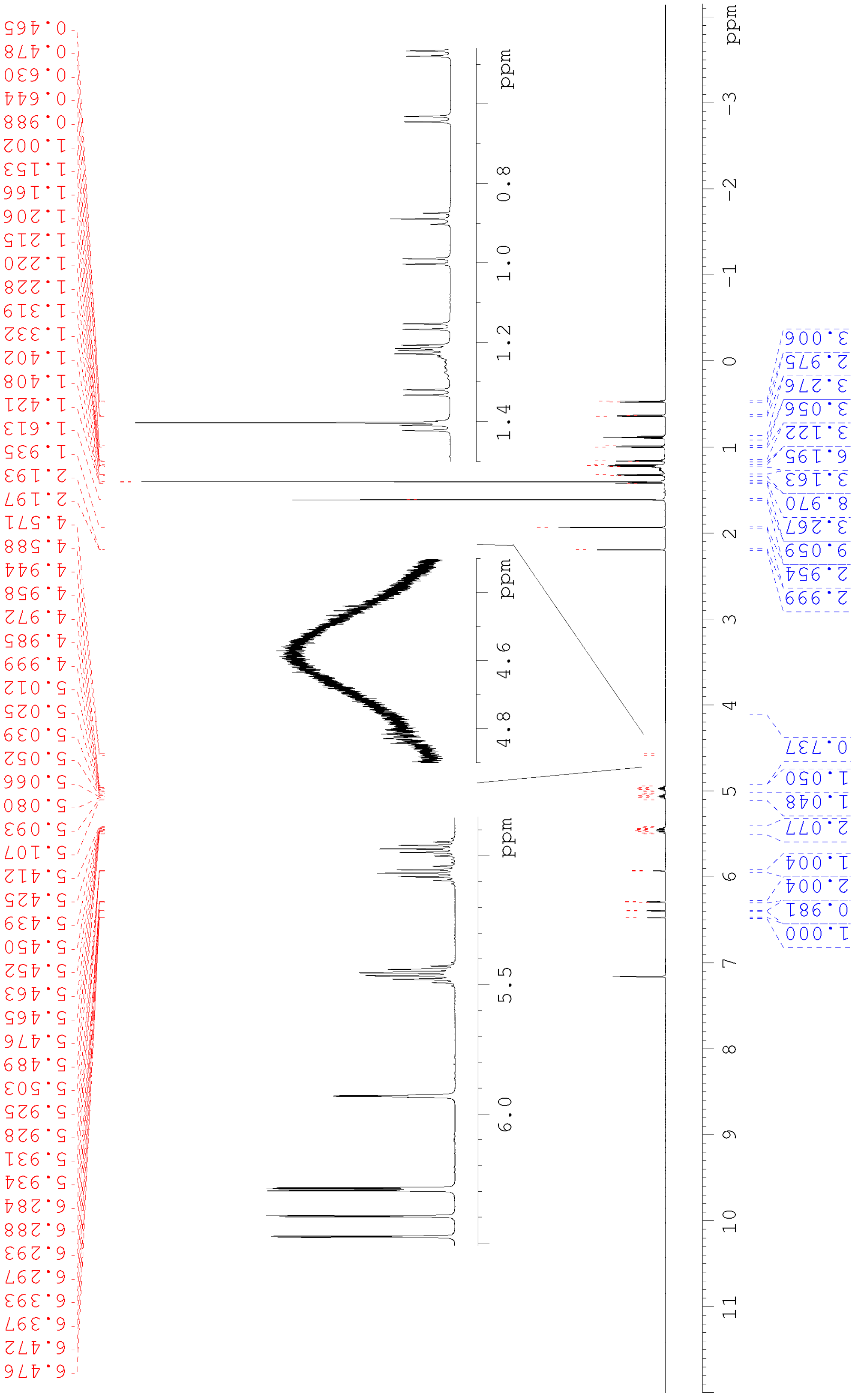

Figure S36. ^{1}H NMR spectrum of **6** in C_6D_6.

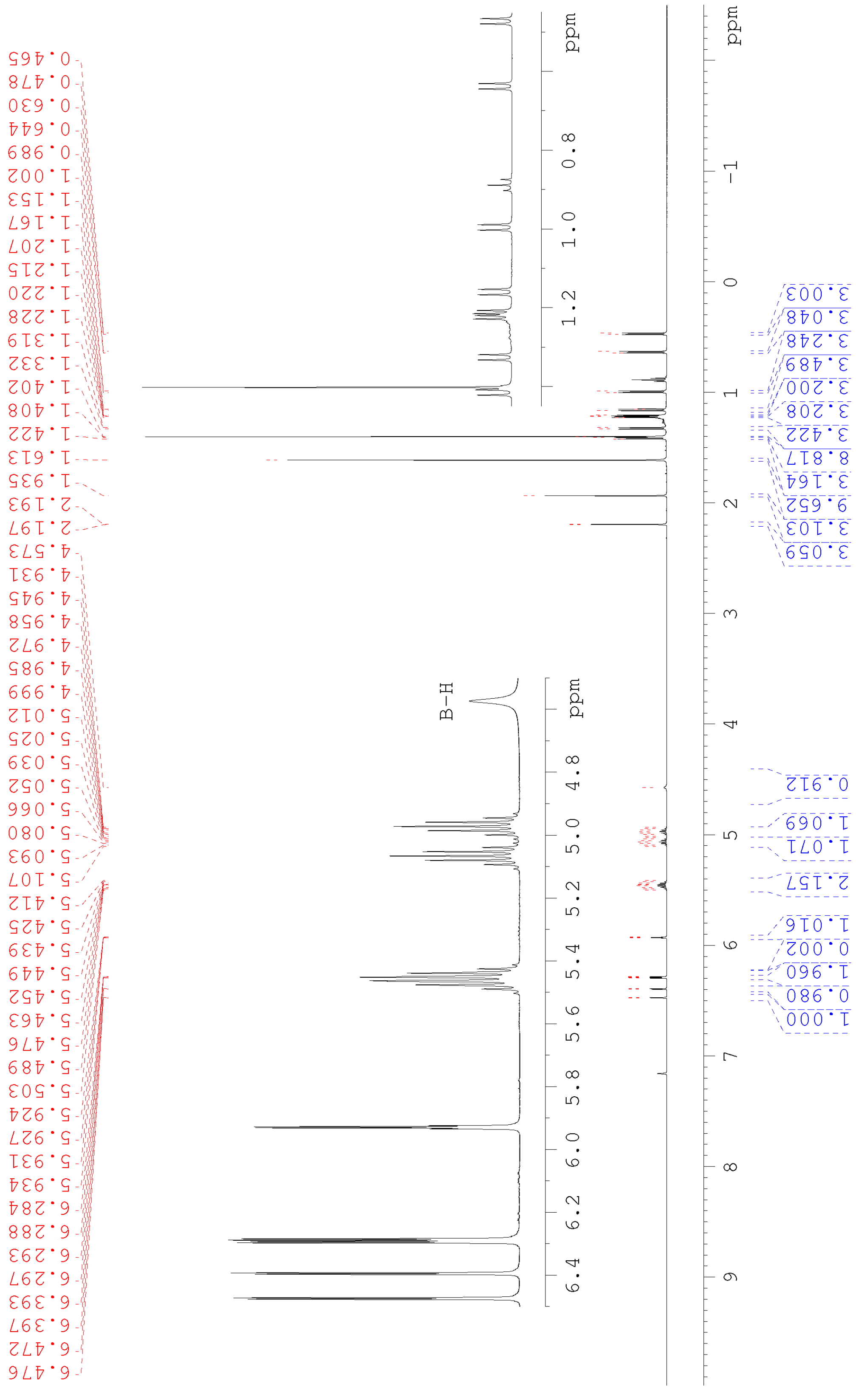

Figure S37. $^{1}H\{^{11}B\}$ NMR spectrum of **6** in C_6D_6.

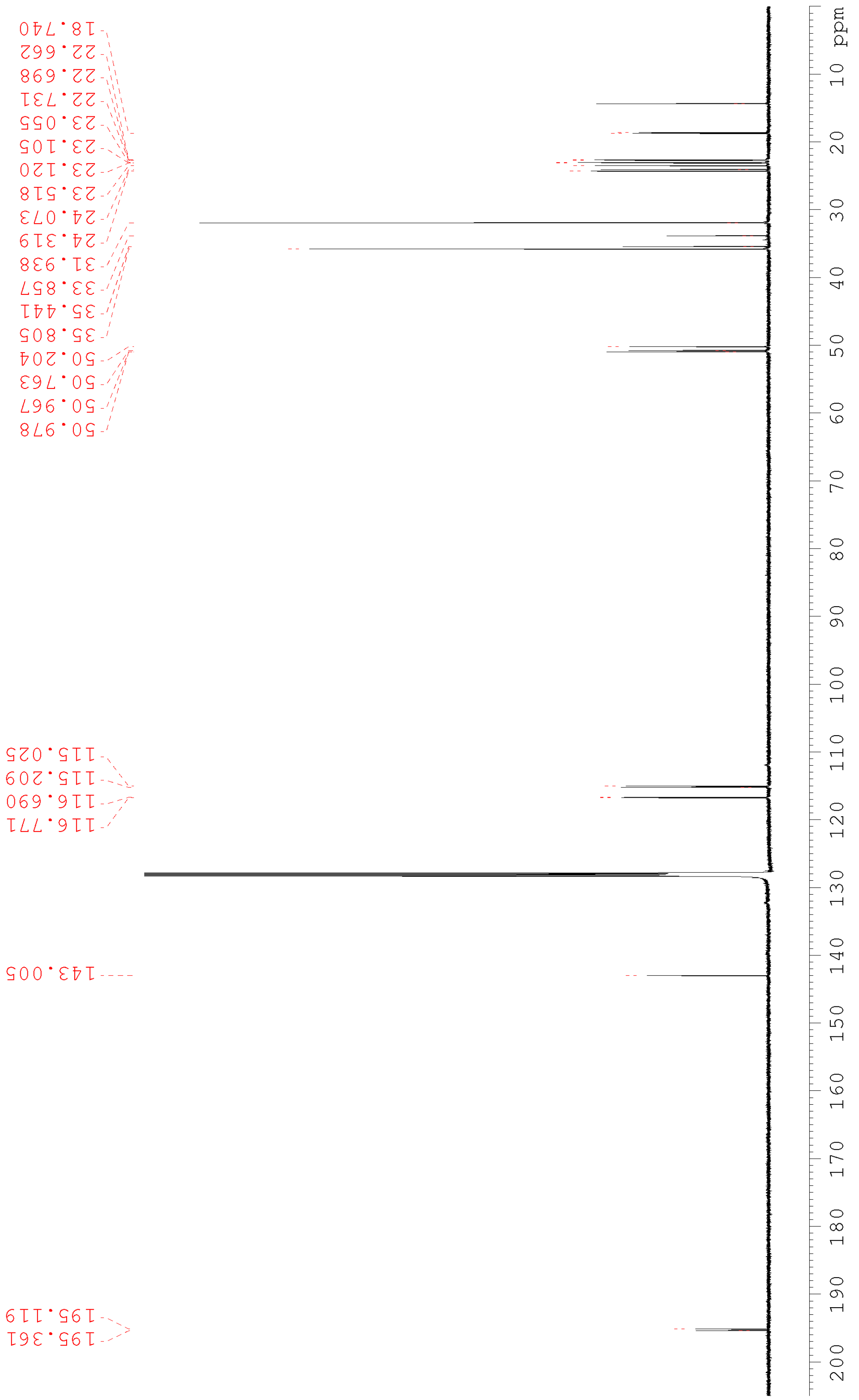

Figure S38. $^{13}C\{^{1}H\}$ NMR spectrum of **6** in C_6D_6.

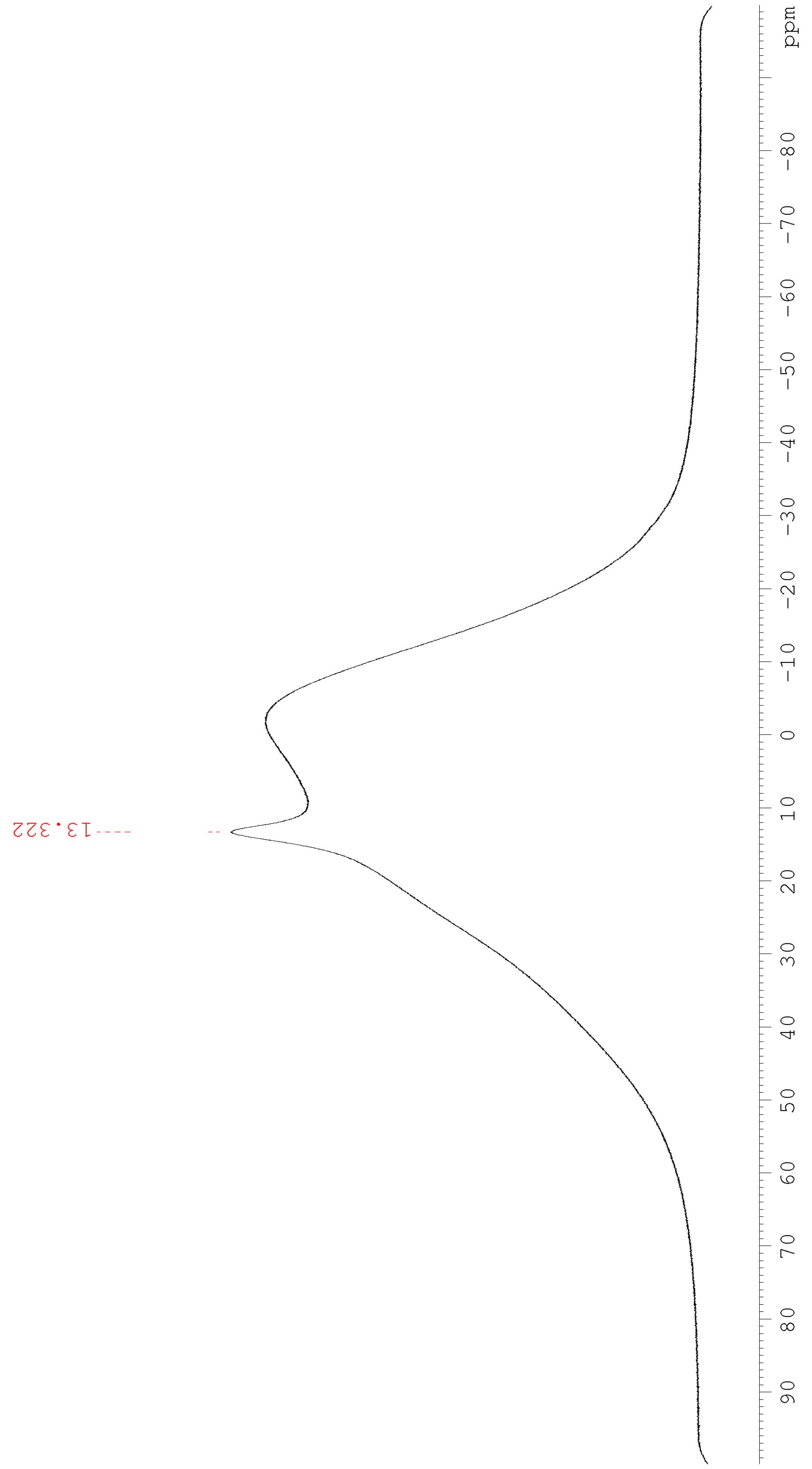

Figure S39. $^{11}B\{^1H\}$ NMR spectrum of **6** in C_6D_6.

NMR spectra of reaction mixtures

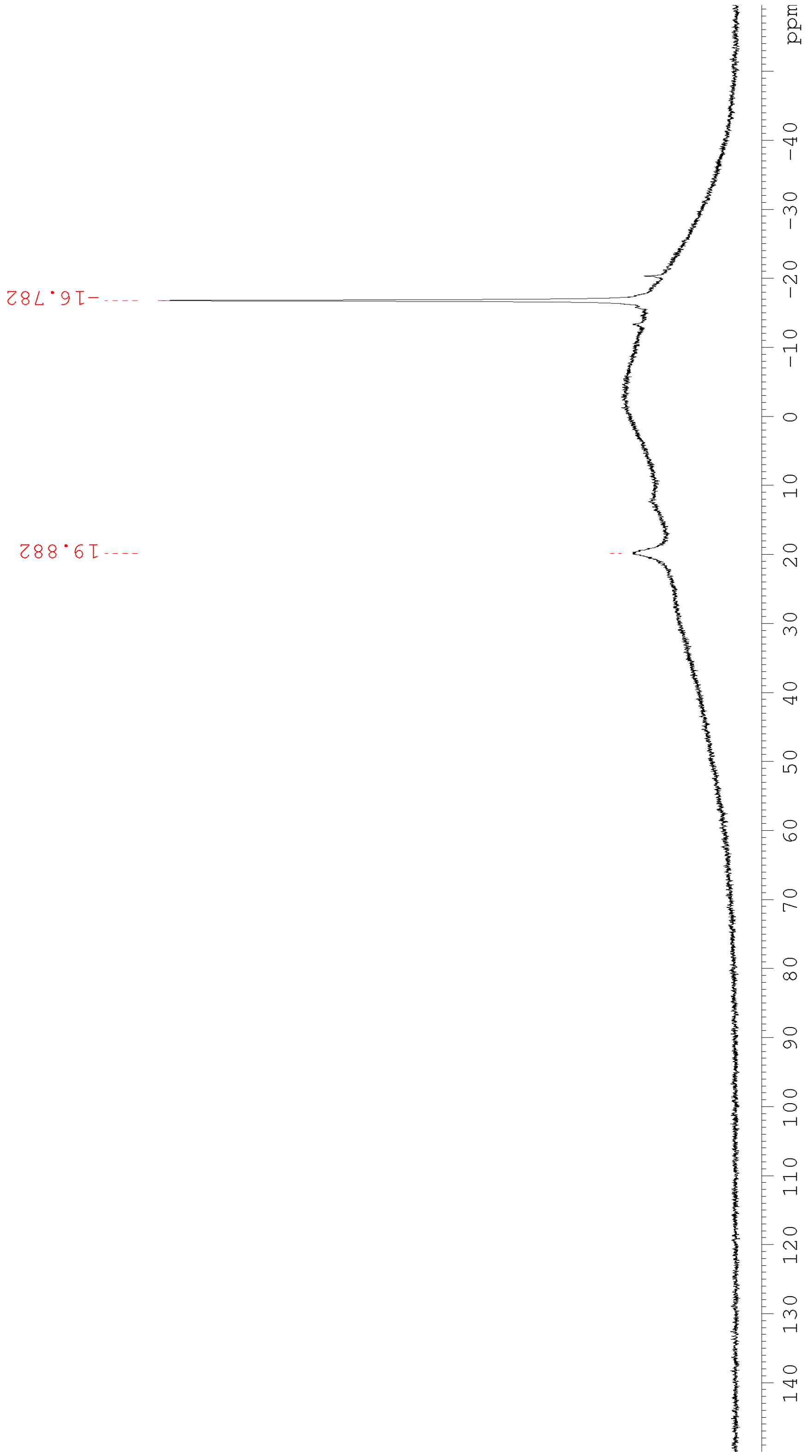

Figure S40. $^{11}B\{^{1}H\}$ NMR spectrum in C_6D_6 of the crude product from the reduction of **2-Me**. The broad resonance at 19.9 ppm is attributed to **3-Me**, the sharp singlet at –16.8 ppm (d in ^{11}B NMR spectrum) to an unidentified decomposition product, presumably from C-H activation.

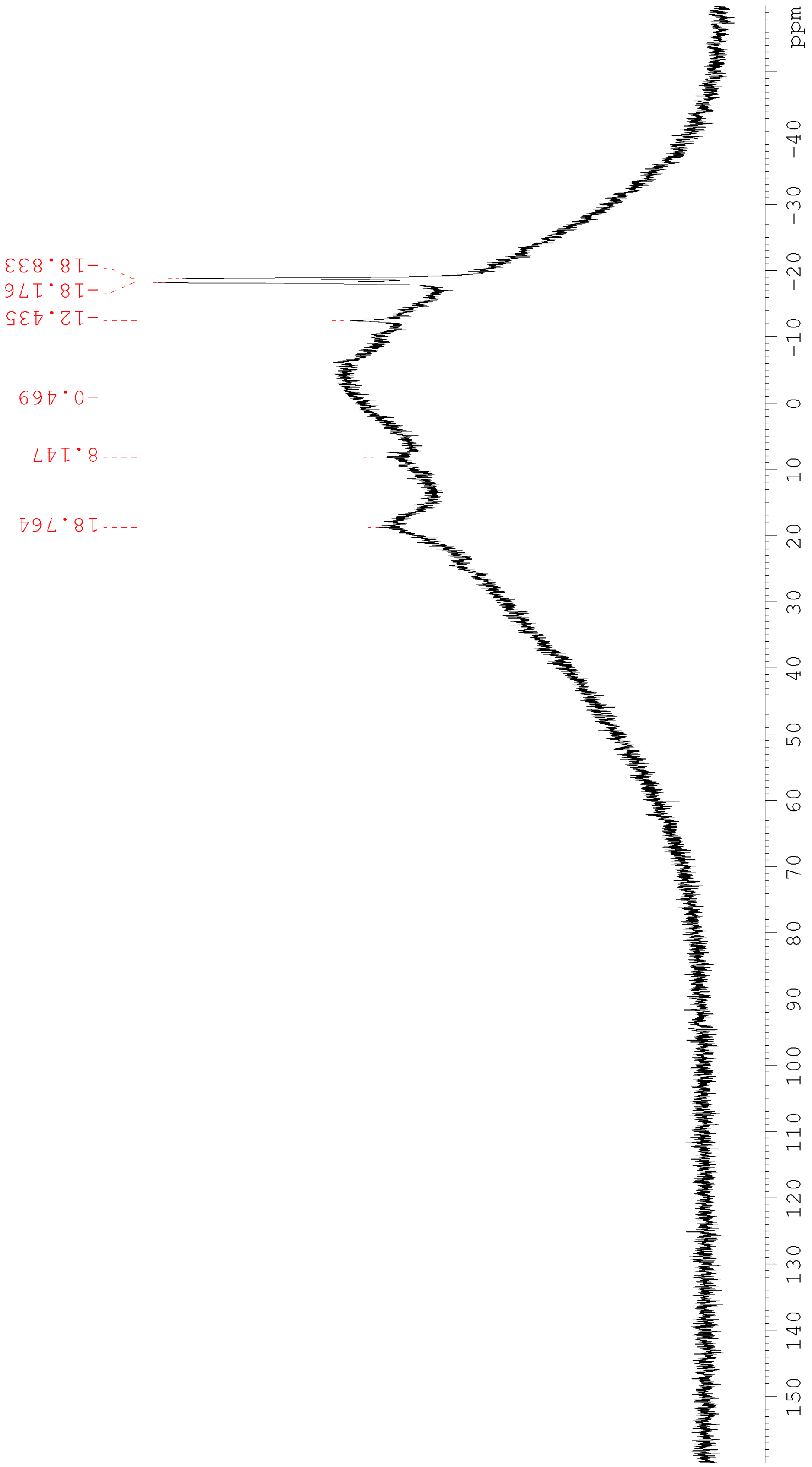

Figure S41. ^{11}B NMR spectrum in C_6D_6 of the crude product from the reduction of **1-Ph**. The broad resonance at 18.8 ppm is attributed to **2-Ph**, the doublet at –18.5 ppm to an unidentified decomposition product, presumably from C-H activation.

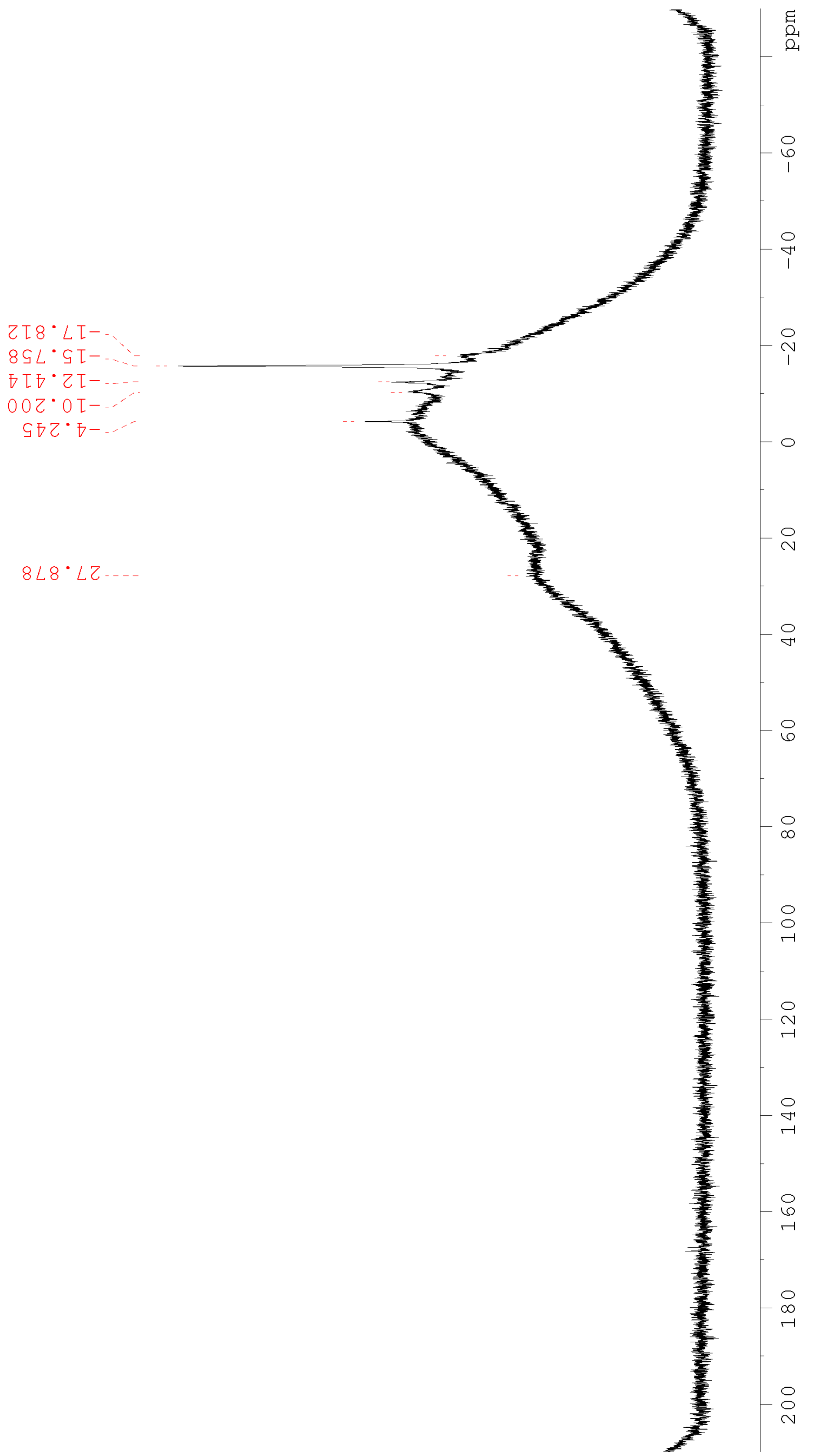

Figure S42. $^{11}B\{^{1}H\}$ NMR spectrum in C_6D_6 of the crude product from the reduction of crude **2-Ph**. The broad resonance at 27.8 ppm is attributed to **3-Ph**, the many singlets below 0 ppm to unidentified decomposition products, presumably from C-H activation.

EPR spectra

EPR measurements at X-band (9.85 GHz) were carried out using a Bruker ELEXSYS E580 (continuous-wave) CW EPR spectrometer. The CW EPR spectrum was measured using 0.2 mW microwave power and 1 G field modulation at 100 kHz, with a conversion time of 40 ms. The spectral simulations were performed using MATLAB 8.6 and the EasySpin 5.2.25 toolbox.[5]

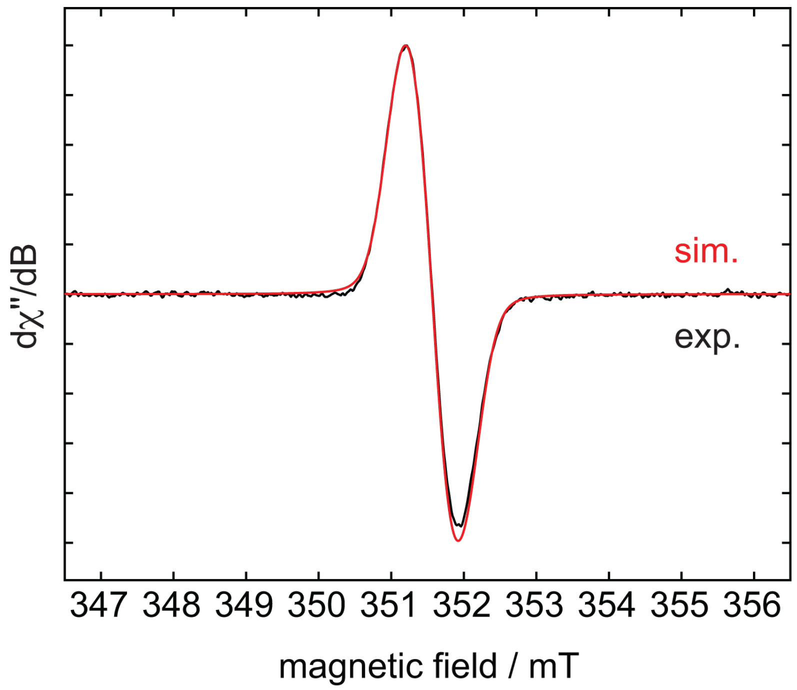

Figure S43. Experimental (black) and simulated (red) CW EPR spectra of **[4]$^{\bullet+}$[BAr$^{F}{}_{4}$]$^{-}$** in 1,2-difluorobenzene at room temperature. Simulated parameters are as follows: g_{iso} = 2.0023, a(B) = ca. 1.7 G (peak-to-peak width = 7 G).

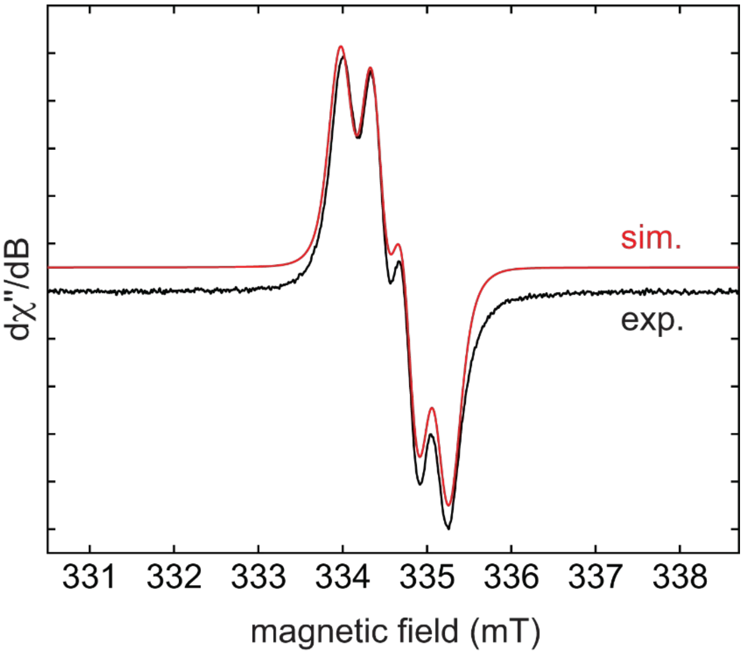

Figure S44. Experimental (black) and simulated (red) continuous-wave (CW) X-band EPR spectra of the radical cation-radical anion salt **[4]**$^{\bullet+}$**[5]**$^{\bullet-}$in 1,2-difluorobenzene. Experimental parameters: temperature = 298 K; microwave frequency = 9.38 GHz; microwave power = 0.5 mW; modulation amplitude = 1 G; conversion time = 40 ms; modulation frequency = 100 kHz. Simulation parameters: g_{iso} = 2.0025, a(B) = 3.5 G (*anion*); g_{iso} = 2.0023, a(B) = < 1 G (*cation*).

UV-vis spectra

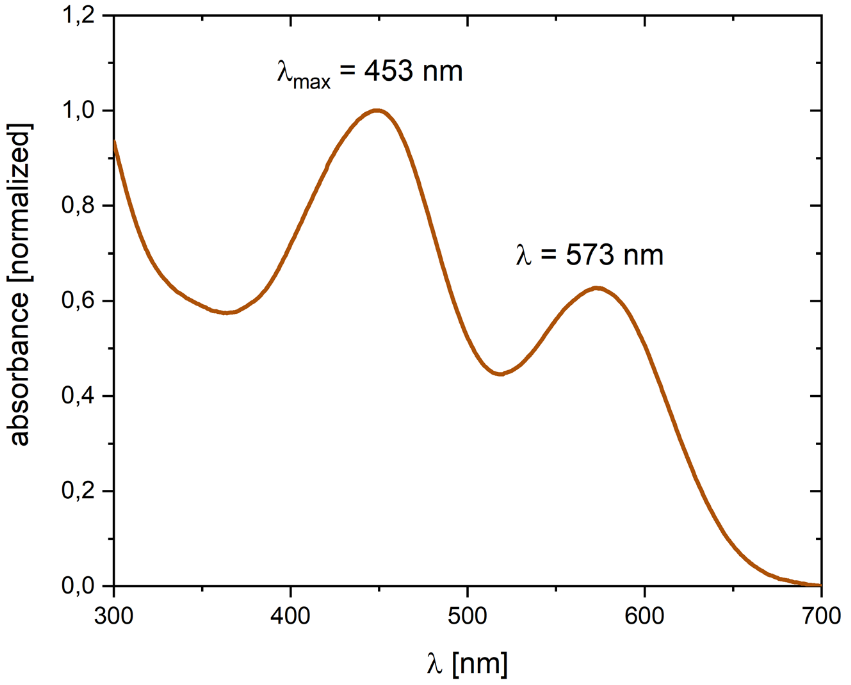

Figure S45. UV-vis absorption spectrum of **4** in benzene (5.76×10^{-4} M).

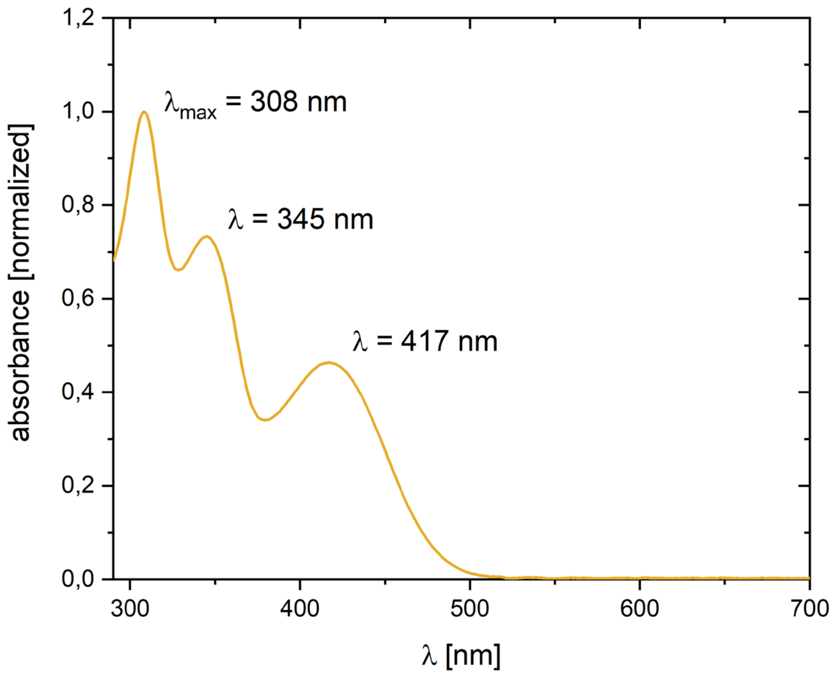

Figure S46. UV-vis absorption spectrum of **4-Cu** in toluene (3.23×10^{-4} M).

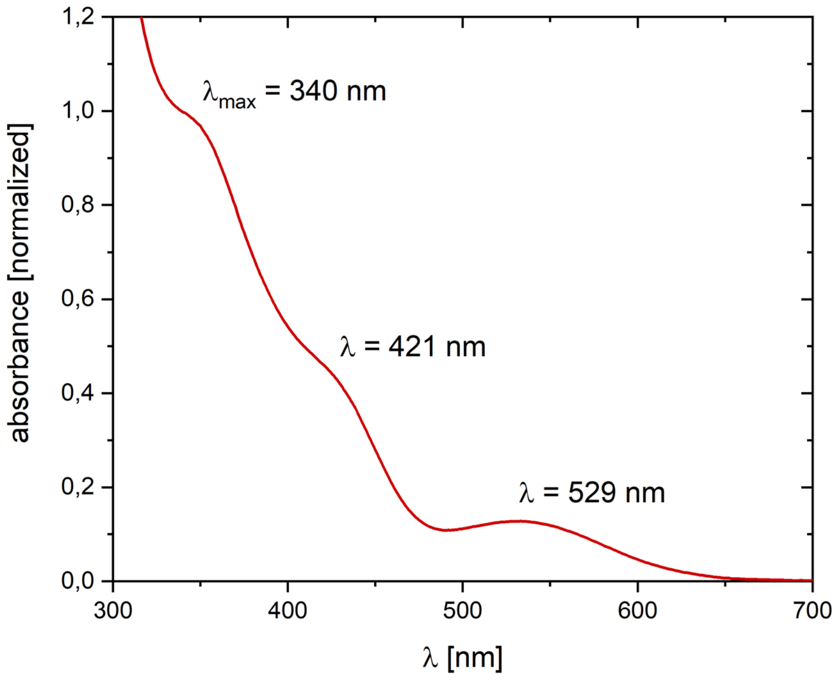

Figure S47. UV-vis absorption spectrum of **4-Pt** in benzene (3.71 x 10^{-4} M).

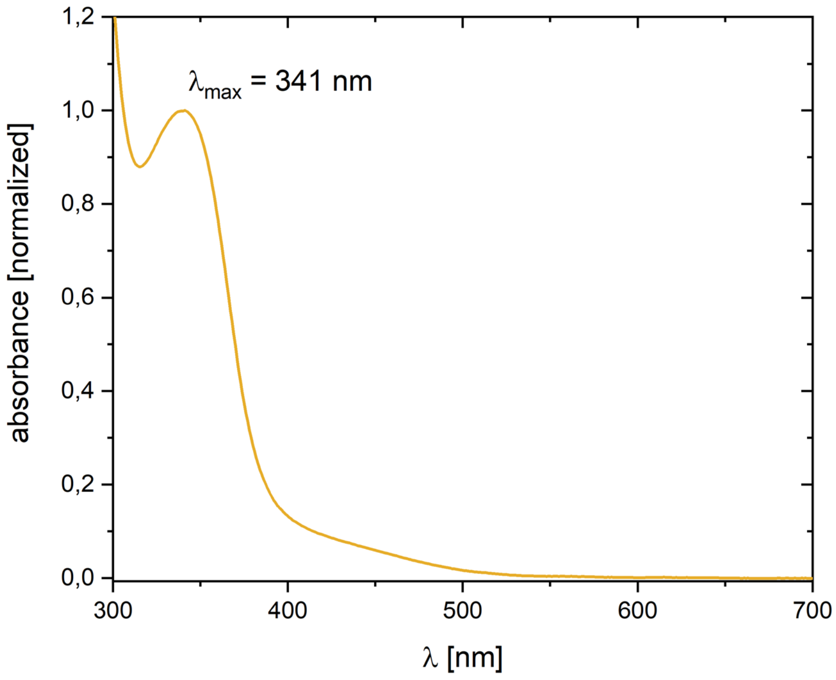

Figure S48. UV-vis absorption spectrum of **6** in benzene (1.74 x 10^{-4} M).

IR spectra

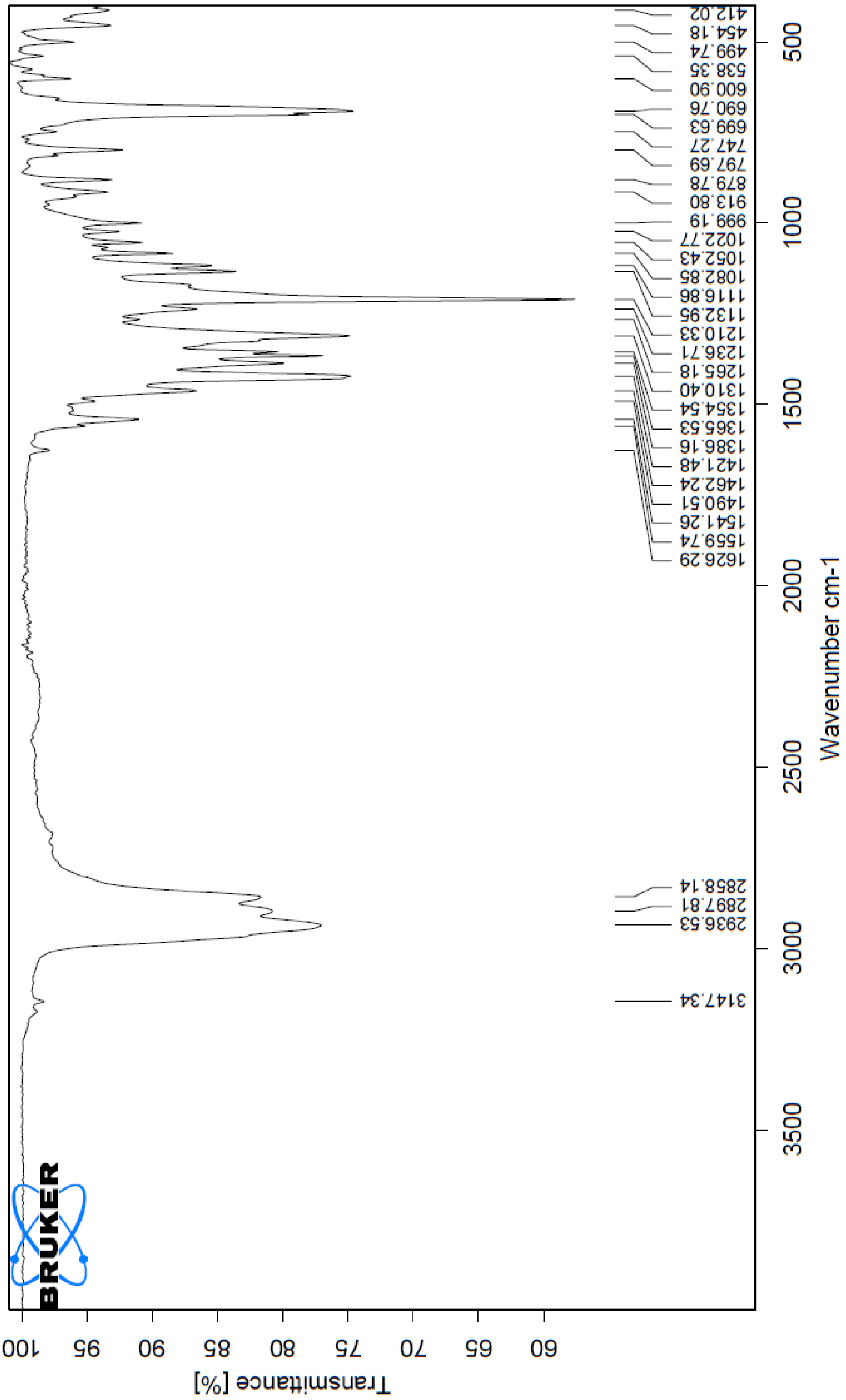

Figure S49. ATR-IR spectrum of **4.**

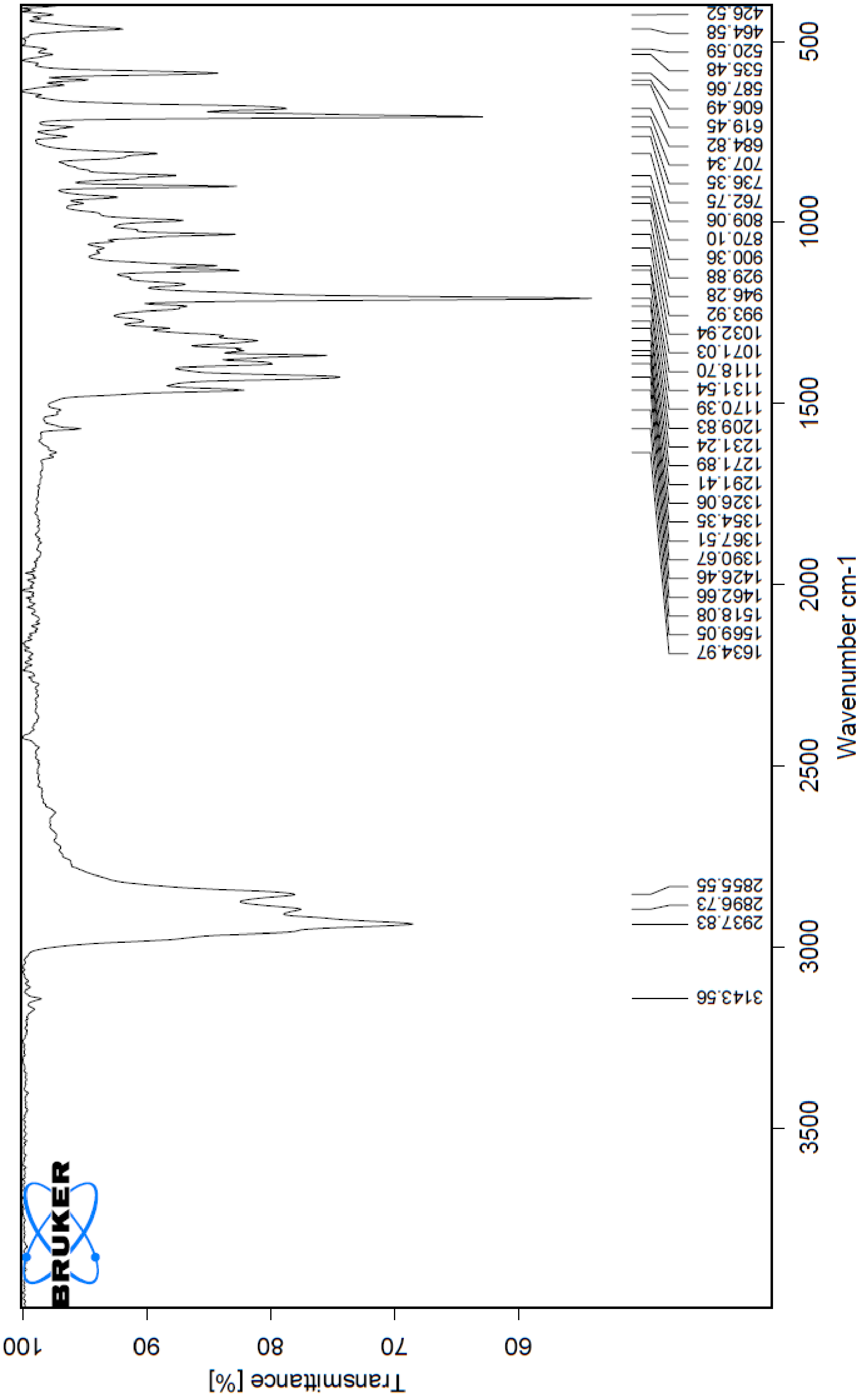

Figure S50. ATR-IR spectrum of **4-Pt**.

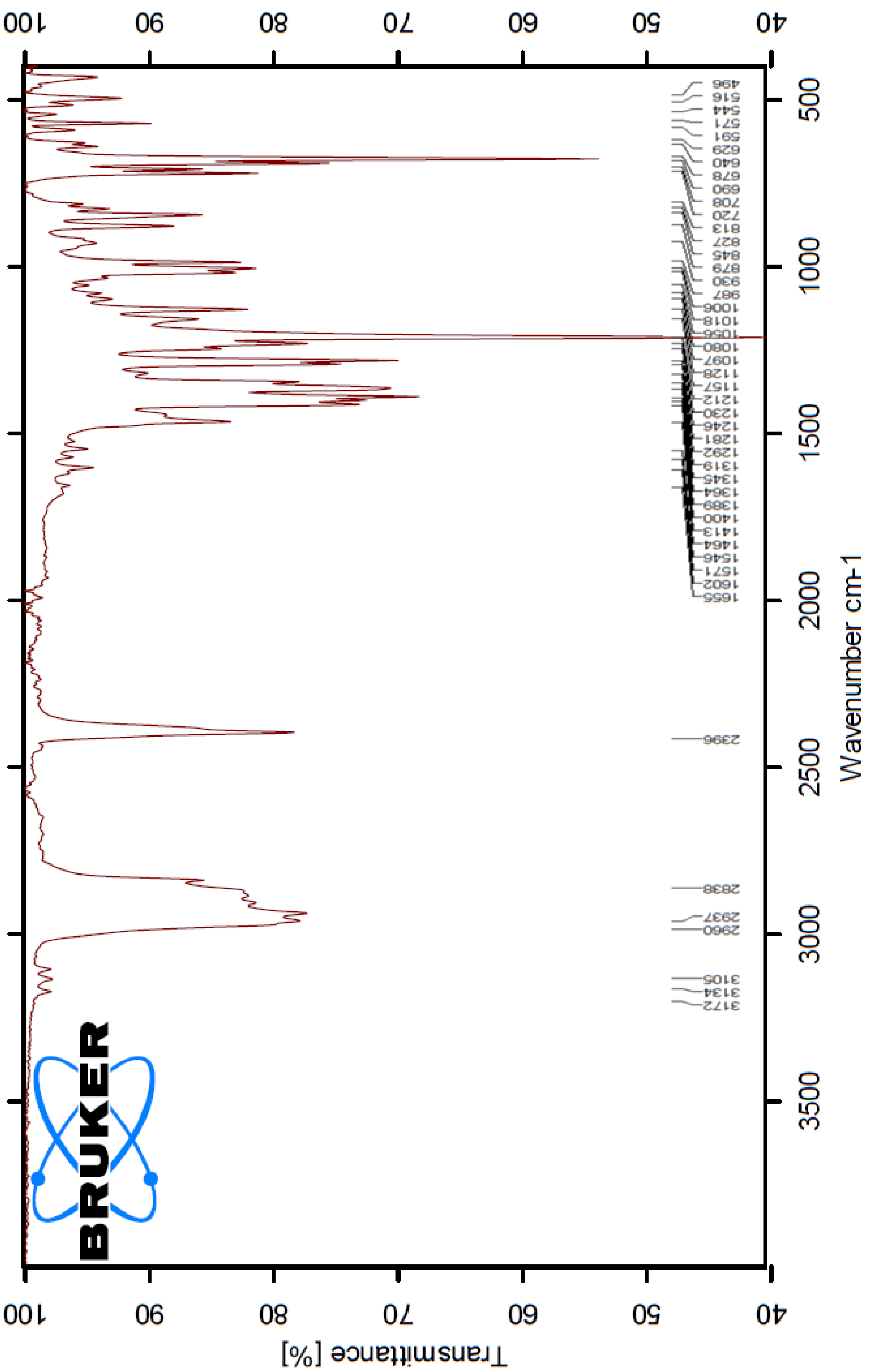

Figure S51. ATR-IR spectrum of **6**.

Crystallographic details

The crystal data of (Me)HC=C(Me)BCat(I*i*Pr), **1-Me**, **2-Me**, **1-Ph,** (*t*Bu)HC=C(Me)BCat(I*i*Pr), **1-*t*Bu** were collected on a Bruker D8 Quest diffractometer with a CCD-area detector or a CMOS area detector and multi-layer mirror monochromated MoK_{α} radiation. The crystal data of **3-Ph** and **4-Cu** were collected on collected on a Bruker X8-APEX II diffractometer with a CCD area detector and multi-layer mirror monochromated MoK_{α} radiation. The crystal data of **2-Ph**, **4**, **4-Pt** and **6** were collected on a *XtaLAB Synergy* diffractometer with a Hybrid Pixel Array detector and multi-layer mirror monochromated CuK_{α} radiation.

The structures were solved using the intrinsic phasing method,[6] refined with the ShelXL program[7] and expanded using Fourier techniques. All non-hydrogen atoms were refined anisotropically. Hydrogen atoms were included in structure factor calculations. All hydrogen atoms were assigned to idealized geometric positions, except that attached to B1 in **6**.

Crystallographic data have been deposited with the Cambridge Crystallographic Data Centre as supplementary publication nos. CCDC 1975949-1975960. These data can be obtained free of charge from The Cambridge Crystallographic Data Centre (www.ccdc.cam.ac.uk/data_request/cif).

Refinement details for (Me)HC=C(Me)BCat(I*i*Pr): The vinyl unit was modelled as twofold disordered in a 51:49 ratio. ADPs within the two parts were restrained with SIMU and RIGU.

Crystal data of (Me)HC=C(Me)BCat(I*i*Pr): $C_{19}H_{27}BN_2O_2$, $M_r = 326.23$, colorless block, $0.362\times0.247\times0.094$ mm^3, monoclinic space group $P2_1/c$, $a = 10.6699(5)$ Å, $b = 13.1268(5)$ Å, $c = 14.0194(5)$ Å, $\beta = 109.6050(10)°$, $V = 1849.75(13)$ Å^3, $Z = 4$, $\rho_{calcd} = 1.171$ g·cm^{-3}, $\mu = 0.075$ mm^{-1}, $F(000) = 704$, $T = 100(2)$ K, $R_1 = 0.0621$, $wR^2 = 0.1117$, 3635 independent reflections [$2\theta \leq 52.04°$] and 262 parameters.

Refinement details for 1-Me: The asymmetric unit contains two half molecules of benzene, each on an inversion center. The first (RESI BZ) is not disordered. The 2nd (RESI Benz) is disordered over two positions in a 43:57 ratio, one directly on the inversion center and the other slightly translated (occupancy 0.285 by symmetry), which was modelled with AFIX 66. ADPs of the benzene molecules were restrained with SIMU and ISOR. The borane displays a 65:35

twofold disorder in the vinyl group (RESI VIN). The second part is mirrored via the B1,C1,N1,N2 plane. ADPs within RESI VIN were restrained with SIMU 0.005. This causes a similar 65:35 disorder in the bromide ligands and one iPr group (RESI iPr C8 > C10). ADPs within RESI iPr were restrained with SIMU 0.005 and distances with SAME.

Crystal data for 1-Me: $C_{13}H_{21}BBr_2N_2{\cdot}(C_6H_6)$, M_r = 417.02, colorless block, 0.314×0.304×0.241 mm^3, triclinic space group $P\bar{1}$, a = 8.104(2) Å, b = 9.183(2) Å, c = 14.522(3) Å, α = 86.504(11)°, β = 83.966(8)°, γ = 89.923(13)°, V = 1072.7(4) Å^3, Z = 2, ρ_{calcd} = 1.291 g·cm^{-3}, μ = 3.775 mm^{-1}, F(000) = 422, T = 100(2) K, R_1 = 0.0392, wR^2 = 0.0699, 4215 independent reflections [$2\theta \leq 52.042°$] and 264 parameters.

Crystal data for 2-Me: $C_{26}H_{46}B_2Br_2N_4{\cdot}(C_6H_6)_2$, M_r = 752.32, colorless block, 0.458×0.261×0.228 mm^3, triclinic space group $P\bar{1}$, a = 11.157(2) Å, b = 13.168(2) Å, c = 15.357(3) Å, α = 65.271(5)°, β = 83.466(9)°, γ = 78.438(9)°, V = 2006.6(7) Å^3, Z = 2, ρ_{calcd} = 1.245 g·cm^{-3}, μ = 2.050 mm^{-1}, F(000) = 788, T = 100(2) K, R_1 = 0.0274, wR^2 = 0.0561, 7891 independent reflections [$2\theta \leq 52.044°$] and 427 parameters.

Crystal data for 1-Ph: $C_{23}H_{27}BBr_2N_2{\cdot}(C_6H_6)_2$, M_r = 658.31, colorless block, 0.423×0.077×0.073 mm^3, triclinic space group $P\bar{1}$, a = 8.078(2) Å, b = 13.537(3) Å, c = 15.050(5) Å, α = 95.204(6)°, β = 93.874(12)°, γ = 100.035(14)°, V = 1608.0(8) Å^3, Z = 2, ρ_{calcd} = 1.360 g·cm^{-3}, μ = 2.546 mm^{-1}, F(000) = 676, T = 100(2) K, R_1 = 0.0596, wR^2 = 0.0765, 6324 independent reflections [$2\theta \leq 52.044°$] and 365 parameters.

Crystal data for 2-Ph: $C_{46}H_{54}B_2Br_2N_4$, M_r = 922.48, colorless block, 0.095×0.079×0.056 mm^3, monoclinic space group $P\ 2_1/n$, a = 17.16238(19) Å, b = 16.61203(16) Å, c = 17.47450(16) Å, α = 90.0°, β = 108.3532(12)°, γ = 90.0°, V = 4728.14(8) Å^3, Z = 4, ρ_{calcd} = 1.296 g·cm^{-3}, μ = 2.460 mm^{-1}, F(000) = 1920, T = 100(2) K, R_1 = 0.0371, wR^2 = 0.0932, 9868 independent reflections [$2\theta \leq 155.03°$] and 549 parameters.

Crystal data for 3-Ph: $C_{46}H_{54}B_2N_4$, M_r = 684.55, orange block, 0.242×0.123×0.067 mm^3, monoclinic space group $C2/c$, a = 20.30(2) Å, b = 9.824(9) Å, c = 19.395(16) Å, β = 102.117(17)°, V = 3782(6) Å^3, Z = 4, ρ_{calcd} = 1.202 g·cm^{-3}, μ = 0.069 mm^{-1}, F(000) = 1472, T = 100(2) K, R_1 = 0.1150, wR^2 = 0.1321, 3708 independent reflections [$2\theta \leq 52.03°$] and 239 parameters.

Crystal data for (*t*Bu)HC=C(Me)BCat(I*i*Pr): $C_{22}H_{33}BN_2O_2$, $M_r = 368.31$, colorless block, $0.257\times0.222\times0.182$ mm^3, monoclinic space group $P2_1/c$, $a = 7.830(2)$ Å, $b = 25.198(7)$ Å, $c = 11.500(3)$ Å, $\beta = 109.119(12)°$, $V = 2143.8(11)$ Å^3, $Z = 4$, $\rho_{calcd} = 1.141$ g·cm^{-3}, $\mu = 0.072$ mm^{-1}, $F(000) = 800$, $T = 100(2)$ K, $R_1 = 0.0679$, $wR^2 = 0.1128$, 4226 independent reflections [$2\theta \leq 52.042°$] and 252 parameters.

Refinement details for 1-*t*Bu: The asymmetric unit contains half of a highly disordered solvent molecule positioned on an inversion center, which could not be modelled satisfactorily. To deal with this electron density the Platon program Squeeze[8] was applied. Overall a total of 37 electrons per unit cell were squeezed, which corresponds to an 88% occupied benzene molecule.

Crystal data for 1-*t*Bu: $C_{16}H_{29}BBr_2N_2$ (+ squeezed solvent), $M_r = 420.04$, colorless block, $2.177\times0.581\times0.424$ mm^3, triclinic space group $P\bar{1}$, $a = 8.2033(15)$ Å, $b = 11.738(3)$ Å, $c = 12.759(4)$ Å, $\alpha = 112.101(7)°$, $\beta = 90.936(8)°$, $\gamma = 103.546(9)°$, $V = 1099.1(5)$ Å^3, $Z = 2$, $\rho_{calcd} = 1.269$ g·cm^{-3}, $\mu = 3.684$ mm^{-1}, $F(000) = 428$, $T = 100(2)$ K, $R_1 = 0.0303$, $wR^2 = 0.0627$, 4326 independent reflections [$2\theta \leq 52.04°$] and 198 parameters.

Crystal data for 4: $C_{32}H_{58}B_2N_4$, $M_r = 520.44$, brown block, $0.234\times0.151\times0.060$ mm^3, triclinic space group $P\bar{1}$, $a = 9.8218(4)$ Å, $b = 10.4000(4)$ Å, $c = 17.9798(7)$ Å, $\alpha = 73.905(3)°$, $\beta = 74.759(3)°$, $\gamma = 86.158(3)°$, $V = 1702.50(12)$ Å^3, $Z = 4$, $\rho_{calcd} = 1.437$ g·cm^{-3}, $\mu = 0.433$ mm^{-1}, $F(000) = 576$, $T = 100(2)$ K, $R_1 = 0.0677$, $wR^2 = 0.1963$, 6599 independent reflections [$2\theta \leq 154.02°$] and 359 parameters.

Refinement details for 4-Cu: The asymmetric unit contains three sites for benzene molecules and two molecules of **4-Cu**. The first benzene molecule (RESI Bz) was modelled as twofold disordered in a 44:56 ratio and its ADPs were restrained with SIMU 0.01. The second and third site were arranged on and around an inversion center with heavy disorders and partial occupancy factors. The Platon program Squeeze[8] was employed to deal with this electron density. A total of 206 electrons per unit cell were squeezed, i.e. 4.9 molecules of benzene, or 1.2 per asymmetric unit. The first of the two **4-Cu** molecules is not disordered (RESI MOL1). The partial occupancy of the second and third benzene molecule sites in the proximity of the second **4-Cu** molecule (RESI MOL2) induced a twofold disorder of the latter over two positions

slightly rotated from each other about the Cu-Cl axis in a 64:36 ratio, which remains shared between the two positions. 1,2- and 1,3-distances within MOL2 were restrained to similarity with SAME, and ADPs with SIMU 0.002.

Crystal data for 4-Cu: $C_{32}H_{58}B_2ClCuN_4{\cdot}(C_6H_6)_{0.5}$ (+ squeezed solvent), $M_r = 658.49$, yellow block, 0.402×0.297×0.16 mm^3, monoclinic space group $P2_1/n$, $a = 17.880(8)$ Å, $b = 16.411(7)$ Å, $c = 29.564(14)$ Å, $\beta = 99.590(14)°$, $V = 8554(7)$ Å^3, $Z = 8$, $\rho_{calcd} = 1.023$ g·cm^{-3}, $\mu = 0.598$ mm^{-1}, $F(000) = 2840$, $T = 100(2)$ K, $R_1 = 0.0868$, $wR^2 = 0.1226$, 16839 independent reflections [$2\theta \leq 52.044°$] and 1207 parameters.

Refinement details for 4-Pt: The crystal was twinned (less than 10%), however, refinement with HKLF 5 did not improve results. For that reason the data was integrated without twinning and the worst reflections were rejected.

Crystal data for 4-Pt: $C_{39}H_{68}B_2N_4Pt$, $M_r = 809.68$, red plate, 0.184×0.095×0.014 mm^3, monoclinic space group $P\ 2_1/c$, $a = 19.9012(4)$ Å, $b = 10.45664(13)$ Å, $c = 19.1527(3)$ Å, $\alpha = 90.0°$, $\beta = 98.0639(17)°$, $\gamma = 90.0°$, $V = 3946.26(11)$ Å^3, $Z = 4$, $\rho_{calcd} = 1.363$ g·cm^{-3}, $\mu = 6.865$ mm^{-1}, $F(000) = 1672$, $T = 100(2)$ K, $R_1 = 0.0380$, $wR^2 = 0.0981$, 8244 independent reflections [$2\theta \leq 156.18°$] and 431 parameters.

Crystal data for 6: $C_{32}H_{58}B_2N_4Ni{\cdot}(C_6H_6)_{0.5}$, $M_r = 622.24$, orange plate, 0.281×0.183×0.046 mm^3, triclinic space group $P\ \bar{1}$, $a = 9.50266(17)$ Å, $b = 11.01177(18)$ Å, $c = 19.1442(4)$ Å, $\alpha = 75.5221(16)°$, $\beta = 84.1295(16)°$, $\gamma = 78.4796(14)°$, $V = 1897.68(6)$ Å^3, $Z = 2$, $\rho_{calcd} = 1.089$ g·cm^{-3}, $\mu = 0.921$ mm^{-1}, $F(000) = 682$, $T = 100(2)$ K, $R_1 = 0.0405$, $wR^2 = 0.0969$, 7867 independent reflections [$2\theta \leq 155.03°$] and 400 parameters.

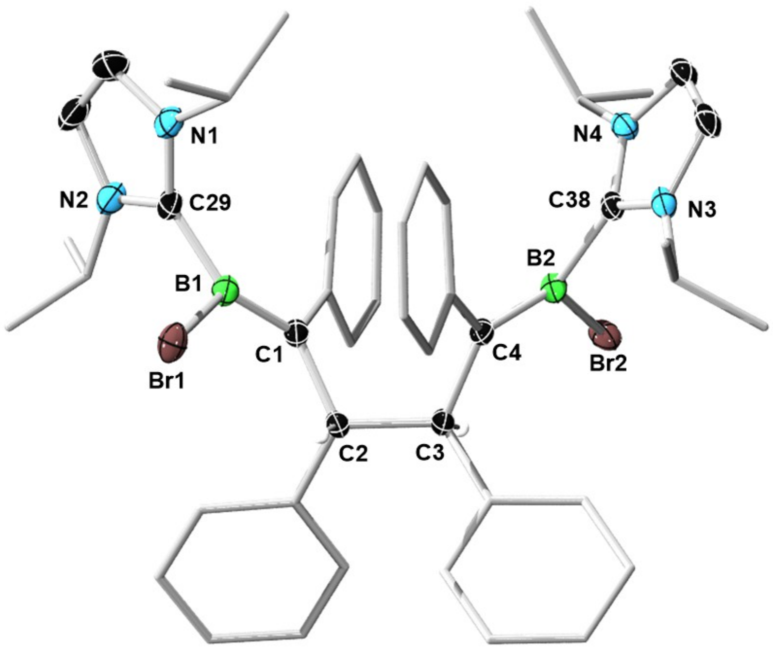

Figure S52. Crystallographically-derived molecular structures of **2-Ph**. Thermal ellipsoids set at 50% probability. Thermal ellipsoids of ligand periphery and hydrogen atoms omitted for clarity.

Computational details

Initially, we performed geometry optimizations and Hessian calculations at the density functional theory level for vinyldiborene **4** starting from the crystal structure. We employed the B3LYP,[9-12] CAM-B3LYP[13] and ωB97XD[14] as functionals and the 6-311G**[15] basis set for these calculations. Scaling factors of 0.967[16] for B3LYP and a Gaussian broadening fitting (bandwidth at half height: 50 cm^{-1}) were used for simulating the infrared spectrum of **4**. We tested the stability of the single-determinant wavefunction[17,18] at the B3LYP/6-311G** level. No restricted/unrestricted Hartree-Fock instability was observed for **4** at the optimized geometry, indicating that the system does not have a biradicaloid character. Time-dependent density functional theory (TDDFT)[19-22] calculations were then performed at the B3LYP/6-311G**, CAM-B3LYP/6-311G** and ωB97XD/6-311G** levels with 15 states in order to simulate the UV-Vis absorption spectrum of **4**. Solvent effects were taken into account with the Solvation Model based on Density (SMD)[23] using benzene as a solvent. The bands of the theoretical spectra are similar, but the wavelengths of the most prominent transitions obtained with CAM-B3LYP (385 and 503 nm) and ωB97XD (383 and 521 nm) are blueshifted in comparison to the experimental values (453 and 573 nm). In contrast, the values obtained at the B3LYP/6-311G** level (439 and 582 nm) are closer to the experimental ones. Therefore, we used this level for characterizing the electronic transitions.

The vinyl units in **4** are not coplanar, and no π conjugation is observed between the C=C and B=B bonds. In order to study the origin of the distortion of the vinyldiborene backbone from coplanarity, we performed geometry optimizations and Hessian calculations (B3LYP/6-311G**) of a model system of **4**, in which H atoms replaced the substituents of the vinyl α-carbon ($\mathbf{4^H}$). For comparison, similar calculations were performed for the all-carbon analogue *trans*-1,3,5-hexatriene. Our results reveal that the $C_2B_2C_2$ backbone is almost coplanar in $\mathbf{4^H}$, and the C–B bonds are shortened from 1.604 Å to 1.566 Å at the B3LYP/6-311G** level of theory. These results, in combination with the analysis of the frontier molecular orbitals, suggest that $\mathbf{4^H}$ displays π conjugation, which is disrupted when replacing the vinyl α-carbon substituent for alkyl groups due to the sterically-induced distortion from planarity.

For the **4-Pt** complex, geometry optimization and Hessian calculations starting from the crystal structure were also calculated at the DFT level. We used ωB97XD as functional and Dunning's cc-pVDZ[24] basis set for all atoms except Pt, which was treated using the pseudopotential-based aug-cc-pVDZ-PP[25] basis set. A scaling factor of 0.953[26] was used to correct the vibrational frequencies of **4-Pt**. In order to investigate the bonding situation in the

system, we used the Energy Decomposition Analysis methodology combined with the Natural Orbitals for Chemical Valence (EDA-NOCV). Therein the molecule is separated into two fragments, whose interaction energy ΔE_{int} is decomposed in three distinct contributions:[27-29]

$$\Delta E_{int} = \Delta E_{elstat} + \Delta E_{Pauli} + \Delta E_{orb} \qquad (S1)$$

where ΔE_{elstat} is the quasi-classical electrostatic attraction, obtained by superimposing the frozen charge densities of the fragments at the optimized geometry of the molecule; ΔE_{Pauli} is the exchange repulsion obtained by antisymmetrizing and renormalizing the product wavefunction; and ΔE_{orb} is the orbital interaction energy, obtained by allowing relaxation of the molecular orbitals to their final form. In the EDA-NOCV approach, the ΔE_{orb} term is calculated by considering the Extended Transition State (ETS) method[26,30] expressed in terms of NOCVs. The ψ_i eigenvectors are the ones that diagonalize the deformation density matrix ΔP in the basis of symmetrized fragment orbitals (SFOs):

$$\Delta \mathrm{P}\psi_i = \nu_i \psi_i \qquad (S2)$$

The deformation density $\Delta\rho$ is obtained by the complementary eigenfunctions ψ_{-k} and ψ_k corresponding to eigenvalues ν_{-k} and ν_k in the following manner:

$$\Delta\mathrm{\rho(r)} = \sum \Delta\mathrm{\rho}_k(r) = \sum \nu_k[-\psi_{-k}^2(r) + \psi_k^2(r)] \qquad (S3)$$

where k represents the NOCV donor-acceptor pairs. The orbital interaction ΔE_{orb} term is then given by:

$$\Delta E_{orb} = \sum \Delta \mathrm{E}_k^{orb} = \sum \nu_k(-F_{-k}^{TS} + F_k^{TS}) \qquad (S4)$$

where $-F_{-k}^{TS}$ and F_k^{TS} are diagonal transition-state Kohn-Sham matrix elements corresponding to NOCVs with eigenvalues ν_{-k} and ν_k.

The EDA-NOCV calculations of **4-Pt** were performed from single-point calculations at the B3LYP/TZV2P level for the optimized geometry at the ωB97XD/cc-pVDZ,aug-cc-pVDZ-PP{Pt} level. Scalar relativistic effects are taken into account by applying the zeroth order regular approximation (ZORA).[31] In addition to EDA-NOCV, we also calculated the Mayer

Bond Order (MBO)[32,33] for **4-Pt** and the metal-free optimized *cis*-η^4-like structure of **4**, namely ***cis*-4**. The bond order defined by Mayer can be obtained from the density matrix P and the atomic orbital overlap matrix S as follows:

$$\mathrm{B}_{AB}^{Mayer} = \sum_{\lambda \in A} \sum_{\omega \in B} (PS)_{\omega\lambda}(PS)_{\lambda\omega} \qquad (S5)$$

where λ and ω represent basis orbitals centered on atoms A and B, respectively. The MBO can be considered as an extension of the Wiberg index,[34] which uses the square of the off-diagonal elements of the matrix P as a criterion for bond order.[35]

We then turned our attention to investigating a plausible reaction mechanism for the transformation of **4** into **6**. Single-point calculations were performed using the dispersion-corrected B3LYP-D3(BJ) and M06-D3 functionals combined with the triple-ζ quality def2-TZVPP basis set and SMD for solvent effects (toluene, ε=2.3741) on optimized geometries at the B3LYP-D3(BJ)/def2-SVP level of theory.[36] Free energy values were collected at 353.15 K, which is the reaction temperature. A scaling factor of 0.96[37] was used to correct the vibrational frequencies. A concentration correction of $\Delta G^{0\rightarrow *}$ = RTln(24.46) = 2.24 kcal mol^{-1} (T = 353.15 K) was included in the free energies of all species in order to account for the change in standard states in going from gas phase (1 atm) to the condensed phase (1 M) and to properly describe associative/dissociative steps.[38] Intrinsic Reaction Coordinates (IRC) were obtained for attesting the connectivity between intermediates and transition states.[39]

In order to describe the bonding situation in **6**, we performed EDA-NOCV and MBO calculations (B3LYP/TZV2P) on the system. We described two distinct scenarios for the EDA-NOCV calculations: one in which a $(NHC)_2Ni(0)$ center interacts with the neutral C_2B_2 1,3-azaborete fragment, and another in which a NHC-stabilized Ni(II) fragment interacts with a dianionic $[C_2B_2]^{2-}$ fragment. Such calculations were performed with solvent effects, included by using the Conductor like Screening Model (COSMO) of solvation (solvent = toluene).[40]

All geometry optimizations, TDDFT and Hessian calculations were performed with the Gaussian 16, Revision B.01 program.[41] The EDA-NOCV and MBO analysis were carried out using the ADF 2018 software.[42] The frontier orbitals, molecular structures and deformation densities were visualized and plotted with ADFview and CYLView.

Geometrical Parameters

Table S1. Comparison of the geometrical parameters of the crystallographically-derived and the computed equilibrium structures of **4**.

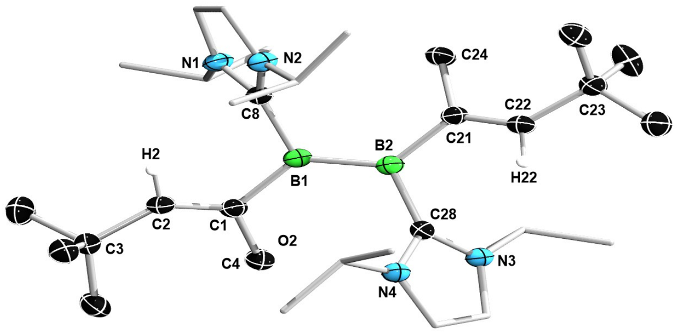

	**4** (X-ray)	**4** B3LYP/6-311G**	**4** CAM-B3LYP/6-311G**	**4** ωB97XD/6-311G**
B1–B2	1.601(2)	1.611	1.588	1.582
B1–C1	1.600(2)	1.604	1.603	1.568
B1–C8	1.581(2)	1.590	1.588	1.576
B2–C21	1.589(2)	1.604	1.603	1.568
C2–C28	1.584(2)	1.590	1.588	1.576
C1–C2	1.354(2)	1.360	1.350	1.345
C21–C22	1.354(2)	1.360	1.350	1.345
torsion (C1,B1,B2,C21)	177.06(16)	174.43	175.69	172.68
torsion (C8,B1,B2,C28)	164.82(14)	174.86	174.76	168.33

Table S2. Comparison of the geometrical parameters of the crystallographically-derived and the computed equilibrium structure (ωB97XD/cc-pVDZ,aug-cc-pVDZ-PP{Pt}) of **4-Pt**.

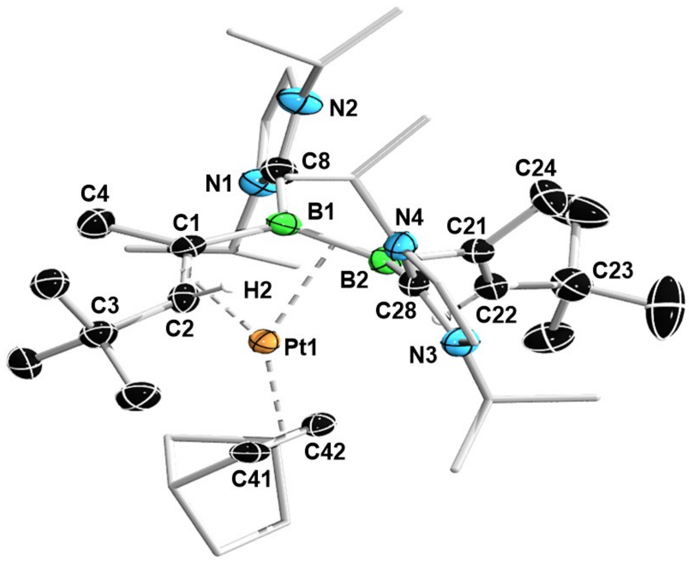

	**4-Pt** **(X-ray)**	**4-Pt** **(calcd.)**
B1–B2	1.637(5)	1.633
B1–Pt1	2.248(4)	2.255
B2–Pt1	2.343(4)	2.367
C1–Pt1	2.227(3)	2.240
C2–Pt1	2.236(3)	2.243
Pt1–C41	2.116(3)	2.105
Pt1–C42	2.076(3)	2.065
B1–C1	1.572(5)	1.568
B1–C8	1.602(5)	1.598
B2–C21	1.621(5)	1.612
B2–C28	1.630(5)	1.614
C1–C2	1.418(5)	1.414
C21–C22	1.348(5)	1.348
angle (B2-B1-C1)	123.3(3)	126.2
angle (B1-C1-C2)	122.0(3)	121.2

Table S3. Comparison of the geometrical parameters of the crystallographically-derived and the computed equilibrium structure (B3LYP-D3(BJ)/def2-SVP) of **6**.

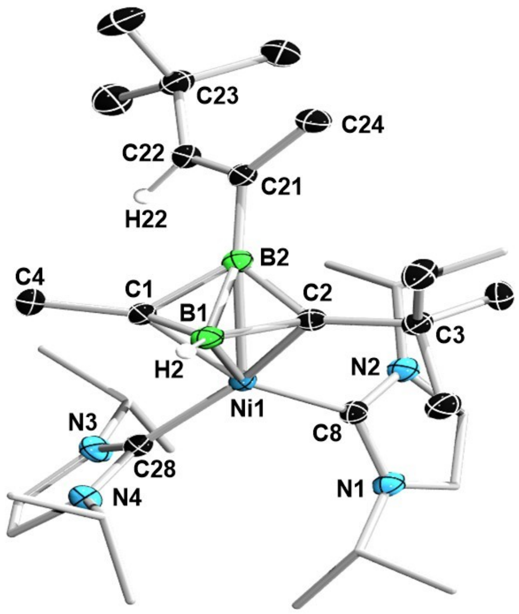

	**6** **(X-ray)**	**6** **(calcd.)**
B1–B2	1.890(2)	1.880
B1–C1	1.5584(19)	1.532
B1–C2	1.5430(19)	1.541
B2–C1	1.5584(19)	1.554
B2–C2	1.5506(19)	1.543
B1–H2	1.106(17)	1.215
B1–Ni1	2.2491(15)	2.287
B2–Ni1	2.2466(14)	2.252
C1–Ni1	1.9710(12)	1.972
C2–Ni1	2.0000(13)	2.015
Ni1–C8	1.9194(13)	1.925
Ni1–C28	1.9111(14)	1.912
C21–C22	1.3396(19)	1.351

Computed IR Spectra

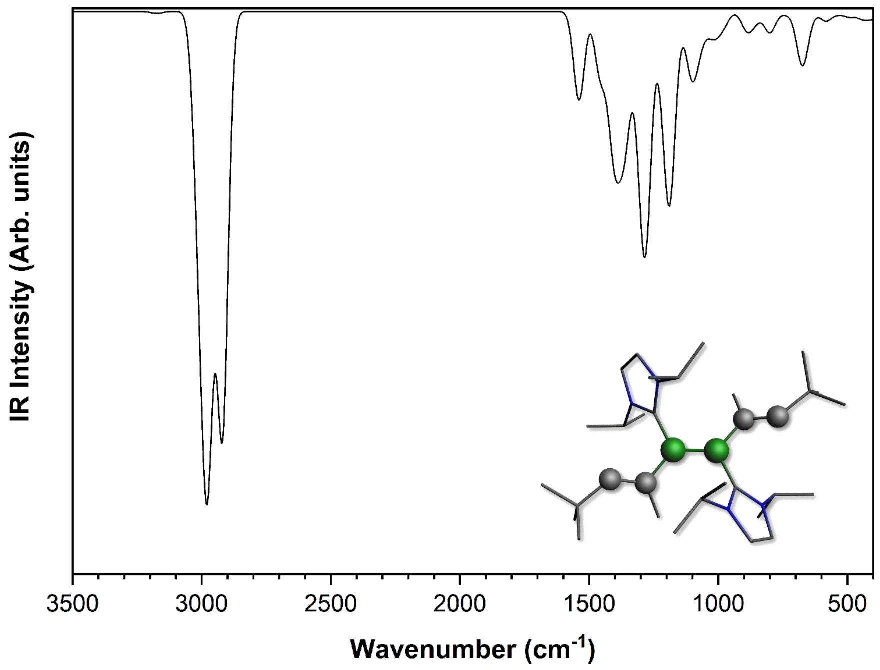

Figure S53. Computed infrared spectrum of **4** (B3LYP/6-311G**, scaling factor: 0.967).

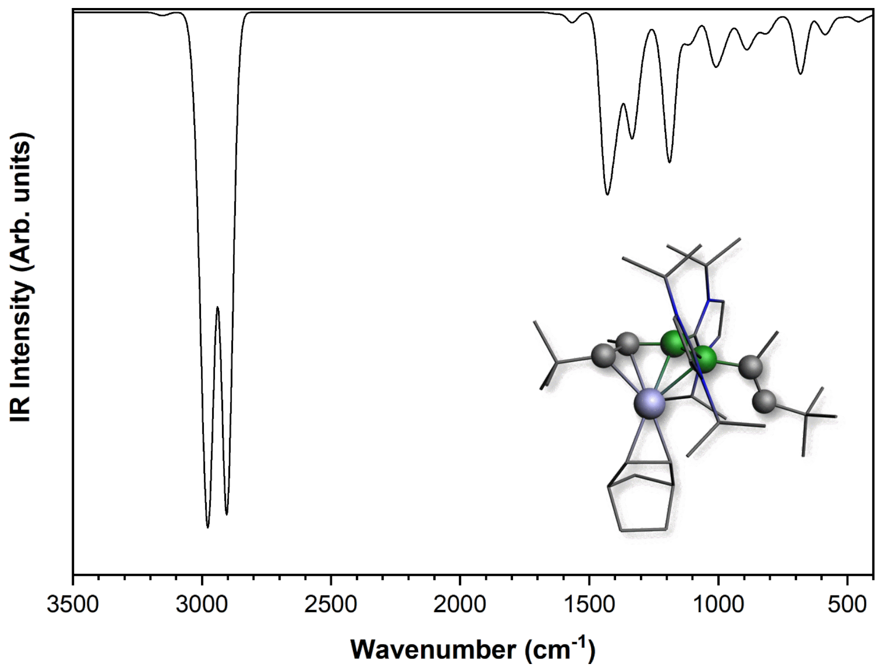

Figure S54. Computed infrared spectrum of **4-Pt** (ωB97XD/cc-pVDZ,aug-cc-pVDZ-PP{Pt}, scaling factor: 0.953).

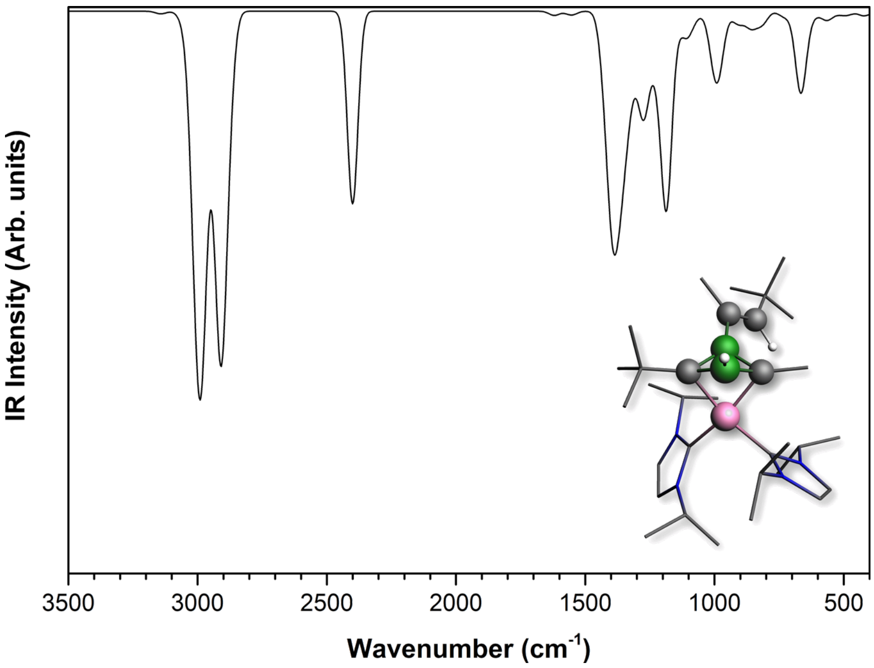

Figure S55. Computed infrared spectrum of **6** (B3LYP-D3(BJ)/def2-SVP, scaling factor: 0.96).

Time-Dependent DFT Calculations

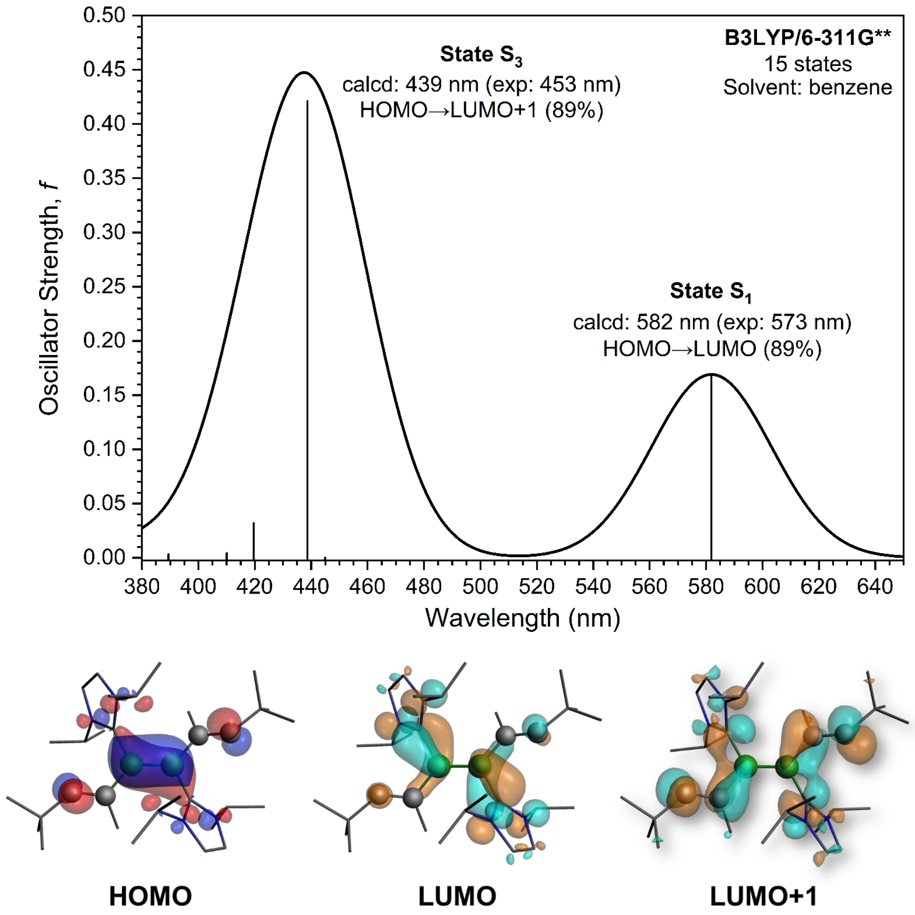

Figure S56. TDDFT calculations (B3LYP/6-311G**) of **4**.

Table S4. TD-DFT results (B3LYP{SMD,Solvent=Benzene}/6-311G**) for the 5 lowest energy vertical transitions of **4**.

State	E (nm)	*f*	Major contributions (%)
S_1	582	0.1691	HOMO → LUMO (89.0)<br>HOMO → LUMO+1 (10.0)
S_2	445	0.0000	HOMO → LUMO+2 (55.8)<br>HOMO → LUMO+5 (30.2)
S_3	439	0.4213	HOMO → LUMO (9.6)<br>HOMO → LUMO+1 (89.0)
S_4	420	0.0032	HOMO → LUMO+2 (42.2)<br>HOMO → LUMO+5 (28.8)<br>HOMO → LUMO+4 (26.7)
S_5	420	0.0322	HOMO → LUMO+3 (98.6)

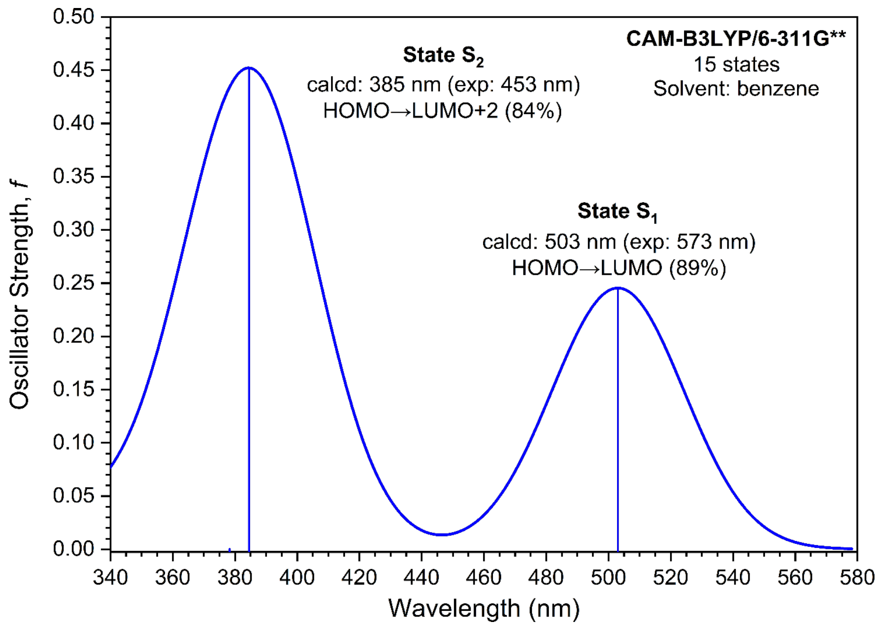

Figure S57. TDDFT calculations (CAM-B3LYP/6-311G**) of **4**.

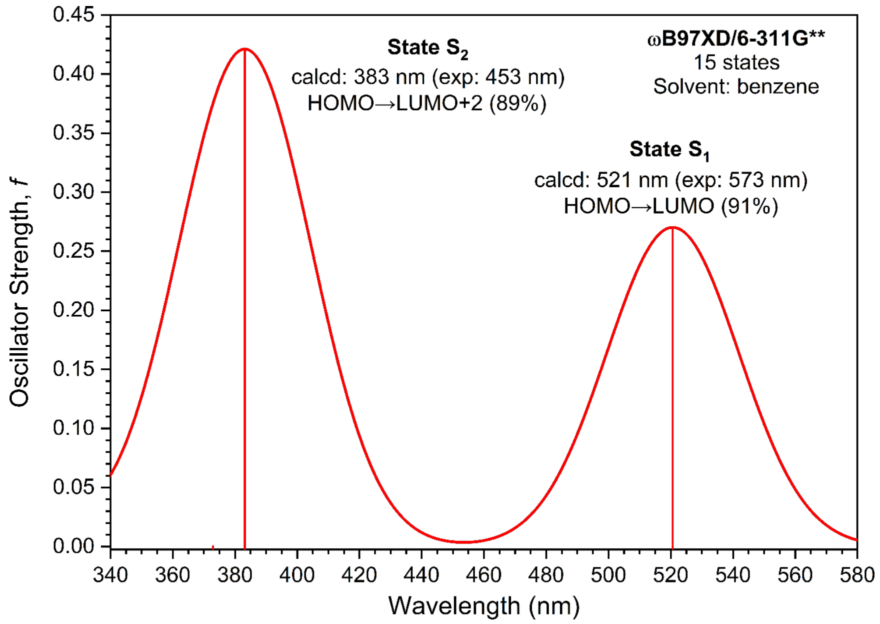

Figure S58. TDDFT calculations (ωB97XD/6-311G**) of **4**.

EDA-NOCV

Table S5. EDA-NOCV results for **4-Pt** and **6**.

Energy Term	Orbital Interaction	Energy (kcal mol^{-1})
**4–Pt (B3LYP/TZV2P)** **Fragments: Pt(nbe) + *cis*–4**		
ΔE_{int}		–70.9
ΔE_{Pauli}		306.6
ΔE_{elstat}[*]		–245.3 (65.0%)
ΔE_{orb}[*]		–132.3 (35.0%)
$\Delta E_{orb(1)}$[†]	$C_2B_2 \rightarrow Pt$	–38.9 (29.4%)
$\Delta E_{orb(2)}$[†]	$Pt \rightarrow C_2B_2$	–32.9 (24.9%)
$\Delta E_{orb(3)}$[†]	$C_2B_2 \rightarrow Pt$	–23.5 (17.8%)
$\Delta E_{orb(rest)}$[†]		–37.0 (28.0%)
**6 (B3LYP/TZV2P{COSMO,Solvent=Toluene})** **Fragments: $(NHC)_2Ni(0)$ + C_2B_2**		
ΔE_{int}		–120.4
ΔE_{Pauli}		273.8
$\Delta E_{solvation}$		0.7
ΔE_{disp}		–27.2 (6.9%)
ΔE_{elstat}[*]		–191.8 (48.6%)
ΔE_{orb}[*]		–175.9 (44.5%)
$\Delta E_{orb(1)}$[†]	$Ni(0) \rightarrow C_2B_2$	–145.7 (82.8%)
$\Delta E_{orb(rest)}$[†]		–30.2 (17.2%)
**6 (B3LYP/TZV2P{COSMO,Solvent=Toluene)** **Fragments: $(NHC)_2Ni(II)$ + $[C_2B_2]^{2-}$**		
ΔE_{int}		–318.7
ΔE_{Pauli}		238.9
$\Delta E_{solvation}$		183.5
ΔE_{disp}		–27.2 (3.6%)
ΔE_{elstat}[*]		–492.6 (66.5%)
ΔE_{orb}[*]		–221.3 (29.9%)
$\Delta E_{orb(1)}$[†]	$[C_2B_2]^{2-} \rightarrow Ni(II)$	–122.8 (55.5%)
$\Delta E_{orb(rest)}$[†]		–98.5 (44.5%)

[*]The values in parentheses show the weight of each contribution with respect to the total attractive interaction.
[†]The values in parentheses show the weight of each contribution with respect to the total orbital interaction, ΔE_{orb}.

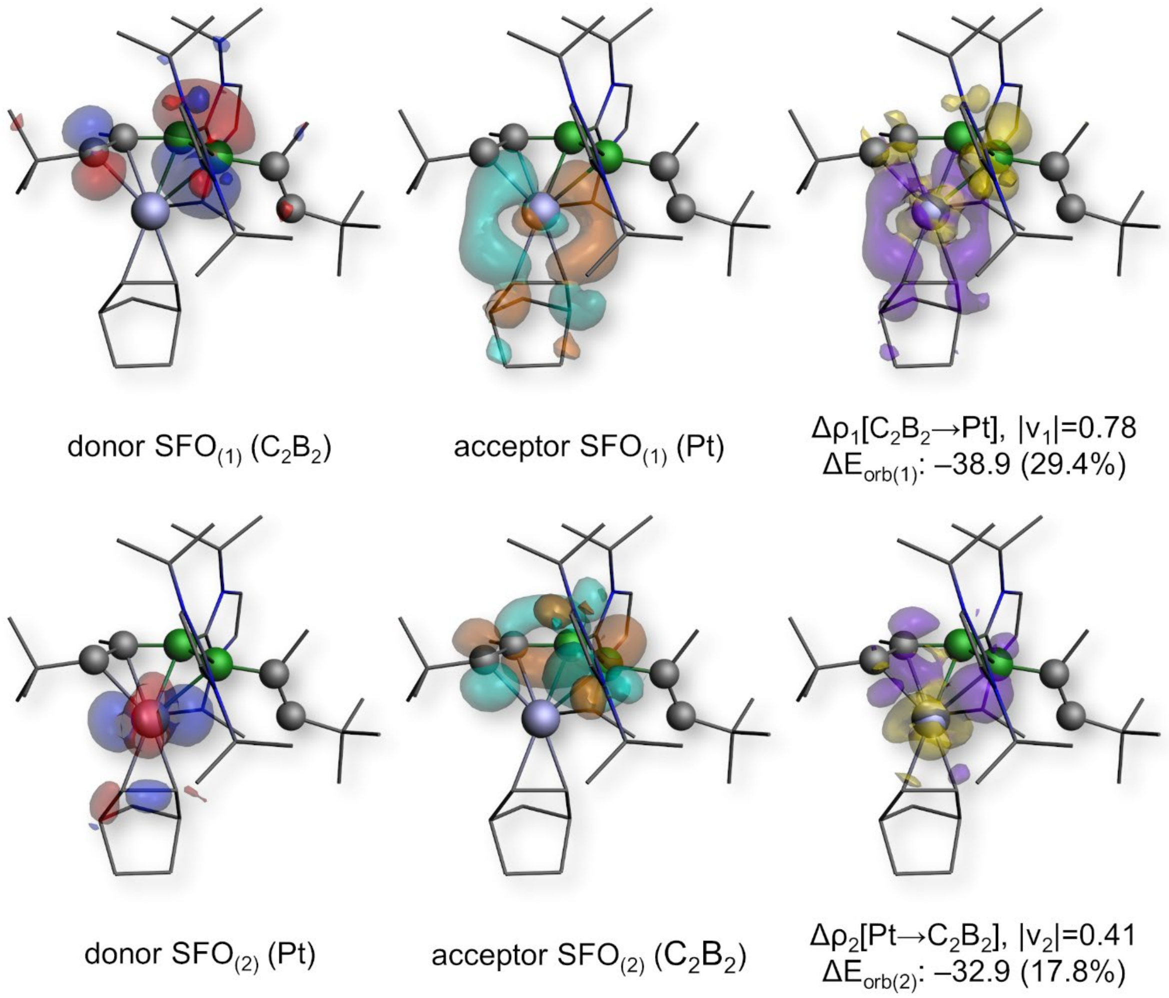

Figure S59. Deformation density contribution and the respective Symmetrized Fragment Orbitals (SFOs) of **4-Pt** (B3LYP/TZV2P//ωB97XD/cc-pVDZ,aug-cc-pVDZ-PP{Pt}). The $|\nu_k|$ values correspond to the eigenvalues of the complementary eigenfunctions (ψ_{-k}, ψ_k) in the NOCV representation, while $\Delta E_{orb(k)}$ is the kth orbital interaction energy (kcal mol^{-1}), with the percentage contribution to the total orbital interaction energy (ΔE_{orb}) shown in parenthesis. The electron density flows from yellow to purple.

Mayer Bond Orders

Table S6. MBOs of ***cis*-4** (B3LYP/TZV2P) and **4-Pt** (B3LYP/TZV2P).

Bond	***cis*-4**		**4-Pt**	
	Bond length (Å)	MBO	Bond length (A)	MBO
C1–C2	1.353	1.75	1.404	1.28
C1–B1	1.597	0.87	1.568	0.89
B1–B2	1.587	1.50	1.633	1.13
Pt1–C2	–	–	2.243	0.51
Pt1–C1	–	–	2.240	0.45
Pt1–B1	–	–	2.255	0.57
Pt1–B2	–	–	2.367	0.56

Table S7. MBOs of **6** (B3LYP/TZV2P).

Bond	Bond length (Å)	MBO
C1–B2	1.554	0.90
C1–B1	1.532	0.95
C2–B2	1.543	0.99
C2–B1	1.541	0.98
C1–C2	2.287	0.00
B1–B2	1.880	0.20
Ni1–C1	1.972	0.77
Ni1–C2	2.015	0.69
Ni1–B2	2.252	0.21
Ni1–B1	2.287	0.18

Cartesian Coordinates

4 (B3LYP/6-311G**)			
**Lowest frequency = 16.39 cm^{-1}**			
N	2.081021000	2.274049000	1.058071000
B	-0.795958000	-0.124672000	0.175111000
N	-2.080242000	-2.274360000	1.058645000
B	0.796043000	0.124708000	0.174677000
N	1.178977000	2.534279000	-0.891295000
N	-1.179564000	-2.533878000	-0.891437000
C	1.993735000	-0.940648000	0.234654000
C	-1.339993000	-1.617099000	0.112655000
C	1.340094000	1.617125000	0.112359000
C	3.196369000	-0.688057000	-0.347979000
H	3.274009000	0.256962000	-0.885992000
C	-1.993682000	0.940606000	0.235543000
C	-3.196498000	0.687975000	-0.346704000
H	-3.274266000	-0.257014000	-0.884753000
C	-2.359671000	-3.567535000	0.654081000
H	-2.917803000	-4.259749000	1.259339000
C	-4.505475000	1.491383000	-0.418481000
C	4.505319000	-1.491480000	-0.420114000
C	2.360161000	3.567371000	0.653782000
H	2.918706000	4.259373000	1.258898000
C	-0.543971000	-2.252089000	-2.196306000
H	-0.033991000	-1.300882000	-2.031004000
C	0.542656000	2.252922000	-2.195906000
H	0.032382000	1.301893000	-2.030521000
C	-1.801863000	-3.729324000	-0.568083000
H	-1.794902000	-4.584300000	-1.220396000
C	-2.435976000	-1.710338000	2.375288000
H	-2.170951000	-0.656590000	2.292207000
C	-1.769925000	2.228414000	1.017690000
H	-2.410261000	3.058405000	0.711271000
H	-0.736461000	2.562574000	0.942084000
H	-1.953689000	2.058769000	2.086392000
C	1.770174000	-2.228433000	1.016886000

H	2.411115000	-3.058151000	0.711028000
H	0.736930000	-2.563161000	0.940689000
H	1.953139000	-2.058482000	2.085677000
C	2.437566000	1.709583000	2.374309000
H	2.173583000	0.655630000	2.290667000
C	1.801515000	3.729599000	-0.567941000
H	1.794115000	4.584809000	-1.219941000
C	0.488489000	-3.322773000	-2.547865000
H	1.010889000	-3.036029000	-3.463546000
H	0.025731000	-4.298973000	-2.726562000
H	1.229358000	-3.424250000	-1.754955000
C	-0.489524000	3.324074000	-2.546851000
H	-1.012568000	3.037582000	-3.462240000
H	-0.026441000	4.300075000	-2.725794000
H	-1.229901000	3.425871000	-1.753522000
C	5.575882000	-0.584223000	-1.066339000
H	6.534416000	-1.105816000	-1.151795000
H	5.736164000	0.320456000	-0.471402000
H	5.271057000	-0.272696000	-2.070342000
C	-3.938599000	-1.834632000	2.631623000
H	-4.197766000	-1.312108000	3.555605000
H	-4.249189000	-2.878005000	2.745854000
H	-4.506530000	-1.388384000	1.814267000
C	4.360124000	-2.744022000	-1.317122000
H	5.322058000	-3.259579000	-1.422715000
H	4.015539000	-2.463152000	-2.316230000
H	3.644356000	-3.459262000	-0.907569000
C	5.035887000	-1.913080000	0.969623000
H	6.006107000	-2.413805000	0.871871000
H	4.360451000	-2.598138000	1.482162000
H	5.174097000	-1.039189000	1.612359000
C	3.940123000	1.835138000	2.630423000
H	4.199950000	1.312187000	3.553975000
H	4.249765000	2.878711000	2.745376000
H	4.508351000	1.390024000	1.812649000
C	-5.576206000	0.584111000	-1.064407000
H	-6.534753000	1.105714000	-1.149659000

H	-5.736362000	-0.320539000	-0.469391000
H	-5.271628000	0.272532000	-2.068468000
C	-5.035715000	1.913069000	0.971352000
H	-6.006027000	2.413652000	0.873789000
H	-4.360243000	2.598290000	1.483621000
H	-5.173642000	1.039247000	1.614240000
C	-4.360465000	2.743895000	-1.315561000
H	-5.322403000	3.259491000	-1.420926000
H	-4.016129000	2.463002000	-2.314748000
H	-3.644565000	3.459105000	-0.906181000
C	-1.590026000	-2.349229000	3.480775000
H	-1.825850000	-1.885886000	4.442392000
H	-0.527456000	-2.203934000	3.280044000
H	-1.786239000	-3.422239000	3.567632000
C	1.591232000	2.347025000	3.480338000
H	1.827756000	1.883431000	4.441663000
H	0.528770000	2.200724000	3.279766000
H	1.786370000	3.420193000	3.567670000
C	1.609068000	2.082350000	-3.283195000
H	1.132744000	1.817983000	-4.231072000
H	2.307763000	1.286993000	-3.016991000
H	2.176626000	3.005071000	-3.439300000
C	-1.610946000	-2.081791000	-3.283082000
H	-1.135156000	-1.817038000	-4.231119000
H	-2.309878000	-1.286794000	-3.016429000
H	-2.178165000	-3.004732000	-3.439121000

**4 (CAM-B3LYP/6-311G**)**			
N	-2.065736000	-2.266994000	1.050668000
B	0.783942000	0.126595000	0.182389000
N	2.065644000	2.267029000	1.050933000
B	-0.784006000	-0.126450000	0.182406000
N	-1.135662000	-2.532499000	-0.870584000
N	1.135909000	2.532664000	-0.870462000
C	-1.991310000	0.927449000	0.228748000
C	1.319253000	1.619858000	0.118862000
C	-1.319260000	-1.619727000	0.118728000

C	-3.175329000	0.664959000	-0.364297000
H	-3.238365000	-0.279733000	-0.904351000
C	1.991211000	-0.927329000	0.228909000
C	3.175223000	-0.665015000	-0.364223000
H	3.238340000	0.279613000	-0.904378000
C	2.330619000	3.561187000	0.653566000
H	2.896499000	4.250459000	1.254418000
C	4.486547000	-1.452702000	-0.438131000
C	-4.486791000	1.452432000	-0.438134000
C	-2.330590000	-3.561143000	0.653189000
H	-2.896518000	-4.250481000	1.253918000
C	0.466003000	2.259658000	-2.152039000
H	-0.046229000	1.312821000	-1.976616000
C	-0.465510000	-2.259395000	-2.152018000
H	0.047022000	-1.312776000	-1.976317000
C	1.753927000	3.726585000	-0.552005000
H	1.729901000	4.584243000	-1.199870000
C	2.446556000	1.692421000	2.347673000
H	2.204157000	0.635468000	2.253464000
C	1.785448000	-2.206340000	1.019112000
H	2.453017000	-3.021634000	0.738934000
H	0.763216000	-2.567681000	0.925612000
H	1.939004000	-2.013999000	2.087073000
C	-1.785640000	2.206533000	1.018862000
H	-2.453334000	3.021729000	0.738702000
H	-0.763466000	2.567999000	0.925235000
H	-1.939060000	2.014233000	2.086850000
C	-2.446765000	-1.692516000	2.347433000
H	-2.204848000	-0.635462000	2.253183000
C	-1.753681000	-3.726460000	-0.552290000
H	-1.729491000	-4.584093000	-1.200179000
C	-0.561032000	3.336806000	-2.468963000
H	-1.115865000	3.056624000	-3.365859000
H	-0.093503000	4.306732000	-2.661994000
H	-1.273935000	3.445158000	-1.652693000
C	0.561180000	-3.336819000	-2.469117000
H	1.116326000	-3.056489000	-3.365771000

H	0.093330000	-4.306488000	-2.662651000
H	1.273862000	-3.445781000	-1.652728000
C	-5.533806000	0.547680000	-1.107035000
H	-6.497444000	1.057613000	-1.190676000
H	-5.685508000	-0.368852000	-0.530271000
H	-5.215588000	0.259639000	-2.112598000
C	3.942448000	1.846417000	2.585509000
H	4.226653000	1.315254000	3.495594000
H	4.229101000	2.893825000	2.713187000
H	4.508284000	1.428118000	1.752998000
C	-4.347752000	2.714665000	-1.307011000
H	-5.314908000	3.216584000	-1.415975000
H	-3.986878000	2.454969000	-2.304920000
H	-3.648436000	3.432643000	-0.877732000
C	-5.031084000	1.837900000	0.947816000
H	-6.004083000	2.330441000	0.850682000
H	-4.367516000	2.517131000	1.480844000
H	-5.166383000	0.949733000	1.569458000
C	-3.942565000	-1.847139000	2.585438000
H	-4.226886000	-1.316026000	3.495515000
H	-4.228772000	-2.894653000	2.713236000
H	-4.508681000	-1.429141000	1.752964000
C	5.533742000	-0.548057000	-1.106898000
H	6.497290000	-1.058154000	-1.190573000
H	5.685593000	0.368380000	-0.530024000
H	5.215603000	-0.259843000	-2.112437000
C	5.030732000	-1.838459000	0.947779000
H	6.003631000	-2.331187000	0.850597000
H	4.367012000	-2.517624000	1.480704000
H	5.166202000	-0.950405000	1.569545000
C	4.347270000	-2.714801000	-1.307165000
H	5.314322000	-3.216917000	-1.416156000
H	3.986483000	-2.454909000	-2.305054000
H	3.647781000	-3.432676000	-0.877996000
C	1.602740000	2.297665000	3.463319000
H	1.856893000	1.831542000	4.417455000
H	0.543019000	2.130635000	3.268866000

H	1.776760000	3.372964000	3.556904000
C	-1.602563000	-2.297385000	3.462990000
H	-1.856825000	-1.831388000	4.417160000
H	-0.542942000	-2.129861000	3.268414000
H	-1.776086000	-3.372766000	3.556570000
C	-1.499333000	-2.087253000	-3.259673000
H	-1.000609000	-1.835000000	-4.197819000
H	-2.195548000	-1.284198000	-3.014056000
H	-2.071410000	-3.005300000	-3.420169000
C	1.500004000	2.088175000	-3.259629000
H	1.001460000	1.835902000	-4.197864000
H	2.196535000	1.285361000	-3.014125000
H	2.071712000	3.006489000	-3.419908000

4 (ωB97XD/6-311G)**

N	-2.103022000	-2.310773000	0.965846000
B	0.781055000	0.134734000	0.279636000
N	2.102902000	2.310528000	0.966630000
B	-0.781103000	-0.134808000	0.279513000
N	-0.974940000	-2.500022000	-0.855528000
N	0.975052000	2.500200000	-0.854844000
C	-1.990002000	0.909538000	0.311225000
C	1.278657000	1.624895000	0.137362000
C	-1.278683000	-1.624941000	0.136835000
C	-3.154974000	0.640258000	-0.317986000
H	-3.191804000	-0.300005000	-0.872032000
C	1.989954000	-0.909615000	0.311205000
C	3.154987000	-0.640155000	-0.317818000
H	3.191862000	0.300254000	-0.871612000
C	2.301626000	3.594916000	0.507297000
H	2.911292000	4.311804000	1.029167000
C	4.477236000	-1.404251000	-0.389218000
C	-4.477211000	1.404385000	-0.389295000
C	-2.301672000	-3.595059000	0.506202000
H	-2.911392000	-4.312073000	1.027834000
C	0.198632000	2.151932000	-2.053940000
H	-0.355032000	1.259985000	-1.757106000

C	-0.198356000	-2.151484000	-2.054443000
H	0.355406000	-1.259709000	-1.757289000
C	1.599980000	3.711636000	-0.640160000
H	1.492965000	4.547113000	-1.309259000
C	2.607630000	1.773346000	2.232929000
H	2.367444000	0.711096000	2.190016000
C	1.804865000	-2.177454000	1.124763000
H	2.429629000	-3.015421000	0.806413000
H	0.766220000	-2.508437000	1.105681000
H	2.037649000	-1.977759000	2.178115000
C	-1.804987000	2.177155000	1.125147000
H	-2.429770000	3.015192000	0.807019000
H	-0.766352000	2.508181000	1.106191000
H	-2.037792000	1.977149000	2.178435000
C	-2.607889000	-1.773894000	2.232219000
H	-2.367786000	-0.711616000	2.189547000
C	-1.599887000	-3.711510000	-0.641198000
H	-1.492782000	-4.546837000	-1.310470000
C	-0.787311000	3.256238000	-2.401700000
H	-1.443062000	2.913791000	-3.204772000
H	-0.288679000	4.167957000	-2.747805000
H	-1.410451000	3.495924000	-1.538750000
C	0.787460000	-3.255822000	-2.402465000
H	1.443367000	-2.913189000	-3.205330000
H	0.288735000	-4.167340000	-2.748961000
H	1.410457000	-3.495915000	-1.539522000
C	-5.505190000	0.488710000	-1.074754000
H	-6.478808000	0.982270000	-1.157763000
H	-5.642783000	-0.438016000	-0.507908000
H	-5.174724000	0.217397000	-2.082536000
C	4.119195000	1.935634000	2.315839000
H	4.495854000	1.430476000	3.208118000
H	4.414697000	2.987742000	2.379511000
H	4.592947000	1.488485000	1.439632000
C	-4.351319000	2.682177000	-1.237971000
H	-5.326199000	3.170795000	-1.350447000
H	-3.972024000	2.442999000	-2.235630000

H	-3.667630000	3.404175000	-0.786924000
C	-5.025087000	1.758080000	1.004641000
H	-6.015922000	2.218975000	0.920041000
H	-4.378408000	2.455615000	1.537858000
H	-5.122082000	0.857950000	1.618591000
C	-4.119448000	-1.936321000	2.314977000
H	-4.496220000	-1.431364000	3.207321000
H	-4.414873000	-2.988463000	2.378424000
H	-4.593165000	-1.489042000	1.438817000
C	5.505289000	-0.488349000	-1.074261000
H	6.478918000	-0.981880000	-1.157323000
H	5.642817000	0.438192000	-0.507098000
H	5.174936000	-0.216708000	-2.081992000
C	5.024955000	-1.758397000	1.004664000
H	6.015818000	-2.219227000	0.920028000
H	4.378241000	-2.456138000	1.537568000
H	5.121842000	-0.858471000	1.618932000
C	4.351445000	-2.681765000	-1.238328000
H	5.326333000	-3.170362000	-1.350827000
H	3.972294000	-2.442257000	-2.235962000
H	3.667680000	-3.403899000	-0.787614000
C	1.857560000	2.404087000	3.400655000
H	2.194131000	1.965035000	4.342929000
H	0.785907000	2.221735000	3.294777000
H	2.028823000	3.484121000	3.447799000
C	-1.857859000	-2.404821000	3.399870000
H	-2.194544000	-1.966000000	4.342211000
H	-0.786214000	-2.222352000	3.294116000
H	-2.029033000	-3.484879000	3.446766000
C	-1.145077000	-1.793447000	-3.195077000
H	-0.571243000	-1.486219000	-4.073250000
H	-1.792898000	-0.965315000	-2.896381000
H	-1.773119000	-2.644558000	-3.477614000
C	1.145508000	1.794394000	-3.194602000
H	0.571797000	1.487333000	-4.072913000
H	1.793443000	0.966279000	-2.896105000
H	1.773434000	2.645691000	-3.476837000

4^H (B3LYP/6-311G**)			
**Lowest frequency = 15.50 cm^{-1}**			
N	1.564872000	2.496475000	1.031128000
B	-0.768500000	-0.227967000	-0.083022000
N	-1.564852000	-2.496478000	1.031198000
B	0.768614000	0.227938000	-0.083070000
N	0.860564000	2.716674000	-1.001333000
N	-0.860830000	-2.716659000	-1.001361000
C	2.015477000	-0.716948000	-0.020822000
C	-1.071711000	-1.785320000	-0.025265000
C	1.071698000	1.785309000	-0.025311000
C	3.333796000	-0.410351000	-0.073784000
H	3.622168000	0.638935000	-0.184461000
C	-2.015365000	0.716932000	-0.020717000
C	-3.333691000	0.410331000	-0.073584000
H	-3.622074000	-0.638954000	-0.184194000
C	-1.645308000	-3.843818000	0.720283000
H	-1.992798000	-4.583655000	1.419368000
C	-4.537677000	1.340728000	-0.024453000
C	4.537791000	-1.340729000	-0.024630000
C	1.645105000	3.843842000	0.720281000
H	1.992589000	4.583684000	1.419361000
C	-0.454617000	-2.392748000	-2.384975000
H	-0.115105000	-1.356914000	-2.312512000
C	0.454319000	2.392778000	-2.384945000
H	0.114766000	1.356958000	-2.312478000
C	-1.212332000	-3.981388000	-0.554472000
H	-1.124398000	-4.860778000	-1.167328000
C	-1.829548000	-1.913439000	2.362401000
H	-1.821669000	-0.837610000	2.186568000
C	1.829768000	1.913413000	2.362273000
H	1.821910000	0.837584000	2.186412000
C	1.211940000	3.981426000	-0.554411000
H	1.123818000	4.860839000	-1.167208000
C	0.710717000	-3.271490000	-2.838041000
H	1.047816000	-2.946996000	-3.825549000

H	0.427108000	-4.325779000	-2.918711000
H	1.550780000	-3.185637000	-2.147564000
C	-0.710974000	3.271566000	-2.838016000
H	-1.048143000	2.947015000	-3.825481000
H	-0.427302000	4.325829000	-2.918792000
H	-1.551016000	3.185829000	-2.147501000
C	5.328559000	-1.206231000	-1.348012000
H	6.240128000	-1.814888000	-1.330464000
H	5.623278000	-0.166550000	-1.522017000
H	4.718612000	-1.526208000	-2.197337000
C	-3.207570000	-2.329353000	2.878776000
H	-3.419524000	-1.805101000	3.813606000
H	-3.262665000	-3.402862000	3.085269000
H	-3.986889000	-2.070219000	2.160489000
C	4.151201000	-2.814169000	0.177085000
H	5.048445000	-3.439829000	0.222424000
H	3.530947000	-3.181242000	-0.645006000
H	3.593488000	-2.956171000	1.106812000
C	5.461074000	-0.907568000	1.138528000
H	6.372469000	-1.515638000	1.169574000
H	4.949044000	-1.010357000	2.099533000
H	5.762468000	0.139088000	1.028609000
C	3.207846000	2.329366000	2.878468000
H	3.419941000	1.805123000	3.813272000
H	3.262938000	3.402878000	3.084944000
H	3.987077000	2.070256000	2.160074000
C	-5.328346000	1.206352000	-1.347908000
H	-6.239893000	1.815041000	-1.330385000
H	-5.623090000	0.166697000	-1.522009000
H	-4.718321000	1.526364000	-2.197160000
C	-5.461063000	0.907499000	1.138595000
H	-6.372451000	1.515578000	1.169611000
H	-4.949115000	1.010215000	2.099652000
H	-5.762461000	-0.139143000	1.028575000
C	-4.151071000	2.814147000	0.177415000
H	-5.048301000	3.439821000	0.222782000
H	-3.530790000	3.181301000	-0.644615000

H	-3.593377000	2.956039000	1.107168000
C	-0.696500000	-2.269723000	3.329362000
H	-0.861588000	-1.780768000	4.293114000
H	0.260761000	-1.931120000	2.928894000
H	-0.643077000	-3.348824000	3.503601000
C	0.696842000	2.269614000	3.329408000
H	0.862072000	1.780605000	4.293108000
H	-0.260465000	1.931010000	2.929051000
H	0.643413000	3.348703000	3.503720000
C	1.661490000	2.470151000	-3.325151000
H	1.368062000	2.163357000	-4.332575000
H	2.457240000	1.805771000	-2.982736000
H	2.059757000	3.487782000	-3.386876000
C	-1.661783000	-2.470172000	-3.325180000
H	-1.368376000	-2.163310000	-4.332589000
H	-2.457584000	-1.805869000	-2.982741000
H	-2.059979000	-3.487828000	-3.386961000
H	1.789441000	-1.782746000	0.071733000
H	-1.789340000	1.782731000	0.071775000

4-Pt (ωB97XD/cc-pVDZ,aug-cc-pVDZ-PP{Pt})

E = -1915.000224 E_h

ΔΔG(298.15) = 0.939796 E_h

Lowest frequency = 24.15 cm^{-1}

Pt	1.166487000	-0.393891000	-0.210477000
B	-1.012694000	-0.431953000	-0.789933000
B	-0.938942000	0.094723000	0.753897000
C	-0.484194000	-1.732306000	4.075398000
H	-0.028593000	-1.778425000	5.057647000
C	3.221044000	-0.280844000	0.231625000
H	3.581746000	-0.887232000	1.072495000
C	2.530796000	1.000324000	0.466229000
H	2.417429000	1.411651000	1.476705000
C	3.183076000	1.971605000	-0.524846000
H	2.599585000	2.874876000	-0.752109000
C	4.597045000	2.264891000	0.037031000
H	5.083183000	3.079909000	-0.523227000

H	4.556170000	2.571184000	1.093450000
C	5.344856000	0.912969000	-0.172290000
H	6.221175000	1.040327000	-0.828439000
H	5.700909000	0.473941000	0.771824000
C	4.268215000	0.022241000	-0.843692000
H	4.677560000	-0.850258000	-1.371910000
C	3.546669000	1.062124000	-1.714438000
H	2.680326000	0.638117000	-2.240304000
H	4.214195000	1.556942000	-2.439173000
C	-0.103720000	-1.767299000	-2.898408000
H	-0.202014000	-2.806205000	-3.247669000
H	0.875456000	-1.402707000	-3.245338000
H	-0.875352000	-1.178098000	-3.416740000
C	-0.253384000	-1.669012000	-1.383704000
C	0.526559000	-2.514777000	-0.561274000
H	0.162654000	-2.655026000	0.461633000
C	1.460610000	-3.670493000	-0.940187000
C	2.478982000	-3.340790000	-2.042608000
H	3.249838000	-4.127815000	-2.087772000
H	2.969495000	-2.380382000	-1.826559000
H	2.020444000	-3.281390000	-3.038580000
C	0.632421000	-4.900245000	-1.358974000
H	1.289319000	-5.751790000	-1.604508000
H	0.011049000	-4.689453000	-2.242921000
H	-0.039789000	-5.222047000	-0.548293000
C	2.254541000	-4.025699000	0.328719000
H	2.905708000	-4.898280000	0.157592000
H	1.577850000	-4.266308000	1.166340000
H	2.877620000	-3.171739000	0.633400000
C	-1.731972000	0.530699000	-1.844027000
N	-1.269164000	1.529080000	-2.631608000
C	-2.295639000	2.072995000	-3.377631000
H	-2.142273000	2.894140000	-4.067970000
C	-3.424601000	1.399187000	-3.051792000
H	-4.441765000	1.520842000	-3.403355000
N	-3.063489000	0.462282000	-2.105252000
C	0.130271000	1.973459000	-2.723080000

H	0.642833000	1.407310000	-1.927205000
C	0.229923000	3.459511000	-2.416896000
H	1.288193000	3.755193000	-2.385249000
H	-0.219980000	3.670856000	-1.439870000
H	-0.267871000	4.077267000	-3.182500000
C	0.716288000	1.621244000	-4.084861000
H	1.778241000	1.905077000	-4.106659000
H	0.204046000	2.161332000	-4.898161000
H	0.645906000	0.543758000	-4.281990000
C	-3.954693000	-0.514256000	-1.468187000
H	-3.500899000	-0.663289000	-0.479405000
C	-3.925933000	-1.838443000	-2.223835000
H	-4.574408000	-2.572529000	-1.721466000
H	-2.902803000	-2.240421000	-2.250824000
H	-4.285943000	-1.713437000	-3.257554000
C	-5.360350000	0.044114000	-1.296026000
H	-5.943452000	-0.642976000	-0.666179000
H	-5.892720000	0.139196000	-2.255827000
H	-5.342454000	1.027192000	-0.802929000
C	-2.908583000	1.526641000	1.728731000
H	-3.307269000	2.496596000	2.051789000
H	-2.983595000	0.836713000	2.589244000
H	-3.595960000	1.129729000	0.961552000
C	-1.491979000	1.545625000	1.187553000
C	-0.752983000	2.667266000	1.070675000
H	0.255988000	2.509591000	0.669131000
C	-1.071590000	4.125687000	1.419458000
C	0.185505000	4.967409000	1.135555000
H	0.021935000	6.023870000	1.403452000
H	0.470620000	4.930064000	0.073691000
H	1.042939000	4.595627000	1.719443000
C	-1.396721000	4.305242000	2.914338000
H	-1.594090000	5.366691000	3.141469000
H	-0.545975000	3.982306000	3.533278000
H	-2.276294000	3.727782000	3.230359000
C	-2.229302000	4.671781000	0.563159000
H	-2.403995000	5.740281000	0.776320000

H	-3.168865000	4.133017000	0.752064000
H	-2.005409000	4.572720000	-0.510447000
C	-0.905221000	-0.898502000	2.025533000
N	-0.230286000	-0.710957000	3.186074000
C	-1.343764000	-2.580015000	3.462714000
H	-1.788211000	-3.504613000	3.811004000
N	-1.591682000	-2.060445000	2.207976000
C	0.692757000	0.399879000	3.454431000
H	0.722618000	0.962420000	2.515667000
C	2.084292000	-0.140708000	3.757930000
H	2.796359000	0.694009000	3.829775000
H	2.417626000	-0.803353000	2.947281000
H	2.113961000	-0.693265000	4.710916000
C	0.130224000	1.291190000	4.553615000
H	0.792526000	2.156782000	4.701282000
H	0.053505000	0.756785000	5.514768000
H	-0.864260000	1.665326000	4.273093000
C	-2.537598000	-2.653600000	1.252356000
H	-2.259928000	-2.201491000	0.291191000
C	-3.963636000	-2.255198000	1.620562000
H	-4.668295000	-2.644143000	0.869617000
H	-4.064013000	-1.161160000	1.660837000
H	-4.253054000	-2.664551000	2.601730000
C	-2.356872000	-4.163247000	1.165395000
H	-2.929032000	-4.545855000	0.307806000
H	-2.722762000	-4.683468000	2.065481000
H	-1.301141000	-4.421650000	1.009342000

***cis*-4 (ωB97XD/cc-pVDZ)**

B	0.155367000	-0.778172000	0.402340000
B	-0.155355000	0.778344000	0.402517000
C	2.717253000	3.100208000	-0.426455000
H	3.231444000	3.733652000	-1.139828000
C	1.806794000	-2.693378000	1.182809000
H	2.689645000	-3.305515000	0.950175000
H	0.928224000	-3.363000000	1.172272000
H	1.904634000	-2.350192000	2.228241000

C	1.568902000	-1.507989000	0.264538000
C	2.487595000	-1.069080000	-0.626941000
H	2.157557000	-0.228601000	-1.249611000
C	3.902199000	-1.546860000	-0.964427000
C	3.874564000	-2.950274000	-1.598339000
H	4.884773000	-3.257315000	-1.919970000
H	3.217375000	-2.960608000	-2.482245000
H	3.500187000	-3.711370000	-0.898571000
C	4.841400000	-1.537523000	0.253868000
H	5.849337000	-1.886526000	-0.028975000
H	4.479300000	-2.183877000	1.065753000
H	4.939077000	-0.516791000	0.655199000
C	4.481966000	-0.571108000	-2.002907000
H	5.512862000	-0.846099000	-2.279628000
H	4.494969000	0.457573000	-1.605851000
H	3.874088000	-0.570736000	-2.922869000
C	-1.106911000	-1.727982000	0.353898000
N	-1.517011000	-2.474659000	-0.706527000
C	-2.717229000	-3.099724000	-0.427607000
H	-3.231590000	-3.732636000	-1.141332000
C	-3.058055000	-2.745406000	0.835934000
H	-3.921039000	-3.018080000	1.432918000
N	-2.058713000	-1.914416000	1.304246000
C	-0.802306000	-2.500517000	-1.987563000
H	-0.137636000	-1.624568000	-1.932061000
C	-1.769293000	-2.322753000	-3.153017000
H	-1.197932000	-2.193810000	-4.083683000
H	-2.401306000	-1.434838000	-3.008075000
H	-2.422558000	-3.199894000	-3.288210000
C	0.045521000	-3.762012000	-2.104037000
H	0.608533000	-3.751261000	-3.049276000
H	-0.583743000	-4.667231000	-2.089872000
H	0.768707000	-3.816532000	-1.279014000
C	-1.979406000	-1.369363000	2.662632000
H	-1.075536000	-0.741288000	2.625270000
C	-1.787521000	-2.497692000	3.671153000
H	-1.651068000	-2.079046000	4.679396000

H	-0.897288000	-3.093567000	3.420687000
H	-2.659671000	-3.171200000	3.702616000
C	-3.177427000	-0.481039000	2.964021000
H	-3.052511000	-0.011050000	3.951014000
H	-4.121579000	-1.050891000	2.982714000
H	-3.256437000	0.307861000	2.205344000
C	-1.806830000	2.693442000	1.183105000
H	-2.689491000	3.305777000	0.950285000
H	-0.928123000	3.362891000	1.172956000
H	-1.905041000	2.350129000	2.228464000
C	-1.568903000	1.508129000	0.264722000
C	-2.487696000	1.069249000	-0.626646000
H	-2.157696000	0.228898000	-1.249492000
C	-3.902486000	1.546785000	-0.963707000
C	-4.482374000	0.570902000	-2.002000000
H	-5.513444000	0.845618000	-2.278344000
H	-4.494935000	-0.457790000	-1.604954000
H	-3.874824000	0.570714000	-2.922180000
C	-3.875345000	2.950201000	-1.597630000
H	-4.885713000	3.257006000	-1.918988000
H	-3.218393000	2.960692000	-2.481712000
H	-3.500955000	3.711381000	-0.897956000
C	-4.841315000	1.537279000	0.254877000
H	-5.849415000	1.886059000	-0.027659000
H	-4.479098000	2.183732000	1.066628000
H	-4.938640000	0.516537000	0.656263000
C	1.106979000	1.728016000	0.354417000
N	1.516898000	2.475451000	-0.705559000
C	3.058321000	2.744960000	0.836753000
H	3.921412000	3.017198000	1.433780000
N	2.059038000	1.913683000	1.304690000
C	0.802208000	2.501970000	-1.986567000
H	0.137095000	1.626356000	-1.931295000
C	1.769084000	2.323954000	-3.152078000
H	1.197647000	2.195564000	-4.082774000
H	2.400568000	1.435628000	-3.007334000
H	2.422866000	3.200743000	-3.287044000

C	-0.045005000	3.763894000	-2.102763000
H	-0.608456000	3.753364000	-3.047740000
H	0.584709000	4.668811000	-2.088923000
H	-0.767767000	3.818875000	-1.277399000
C	1.979796000	1.367947000	2.662806000
H	1.076154000	0.739574000	2.625064000
C	1.787303000	2.495751000	3.671812000
H	1.651090000	2.076638000	4.679895000
H	0.896741000	3.091231000	3.421571000
H	2.659079000	3.169733000	3.703519000
C	3.178125000	0.479967000	2.963973000
H	3.053137000	0.009330000	3.950648000
H	4.122032000	1.050200000	2.983271000
H	3.257652000	-0.308468000	2.204867000

4

E(B3LYP-D3(BJ)) = -1523.826116 E_h

E(M06-D3) = -1522.459729 E_h

ΔΔG(353.15) = 0.752960 E_h

Lowest frequency = 24.15 cm^{-1}

N	-2.130721000	2.334641000	-0.894452000
B	0.792995000	-0.148380000	-0.235532000
N	2.130589000	-2.334747000	-0.894190000
B	-0.793032000	0.148357000	-0.235631000
N	-0.918997000	2.527278000	0.890650000
N	0.918821000	-2.527271000	0.890888000
C	-1.994994000	-0.894568000	-0.323806000
C	1.270537000	-1.637853000	-0.089782000
C	-1.270670000	1.637795000	-0.089986000
C	-3.183733000	-0.639898000	0.291242000
H	-3.228811000	0.286742000	0.879009000
C	1.994971000	0.894477000	-0.323823000
C	3.183742000	0.639822000	0.291172000
H	3.228853000	-0.286810000	0.878951000
C	2.299129000	-3.628770000	-0.433101000
H	2.926963000	-4.355177000	-0.938686000
C	4.512196000	1.403548000	0.298508000

C	-4.512215000	-1.403582000	0.298635000
C	-2.299430000	3.628629000	-0.433329000
H	-2.927289000	4.354992000	-0.938944000
C	0.125056000	-2.169617000	2.079366000
H	-0.430925000	-1.277900000	1.761165000
C	-0.125192000	2.169747000	2.079130000
H	0.430360000	1.277679000	1.761145000
C	1.543984000	-3.747354000	0.693378000
H	1.403113000	-4.592193000	1.359208000
C	2.664283000	-1.807929000	-2.157282000
H	2.527578000	-0.723093000	-2.075556000
C	1.790485000	2.138754000	-1.169326000
H	2.463158000	2.974451000	-0.928816000
H	0.759398000	2.505054000	-1.091415000
H	1.937772000	1.898017000	-2.237832000
C	-1.790509000	-2.138909000	-1.169204000
H	-2.463198000	-2.974578000	-0.928652000
H	-0.759436000	-2.505238000	-1.091221000
H	-1.937737000	-1.898244000	-2.237735000
C	-2.664211000	1.807828000	-2.157631000
H	-2.527832000	0.722965000	-2.075761000
C	-1.544308000	3.747288000	0.693156000
H	-1.403493000	4.592149000	1.358973000
C	-0.862375000	-3.273603000	2.434606000
H	-1.529246000	-2.924269000	3.236489000
H	-0.360814000	-4.186613000	2.797590000
H	-1.486037000	-3.531561000	1.568265000
C	0.862761000	3.273455000	2.433792000
H	1.529560000	2.924199000	3.235770000
H	0.361619000	4.186844000	2.796403000
H	1.486445000	3.530756000	1.567275000
C	-5.554171000	-0.523049000	1.015364000
H	-6.537577000	-1.019089000	1.055683000
H	-5.681037000	0.440428000	0.494932000
H	-5.240067000	-0.303938000	2.048845000
C	4.155700000	-2.102735000	-2.282455000
H	4.562029000	-1.591534000	-3.167925000

H	4.358594000	-3.179751000	-2.401315000
H	4.696393000	-1.738494000	-1.397078000
C	-4.404996000	-2.732165000	1.077220000
H	-5.385891000	-3.235271000	1.134472000
H	-4.053548000	-2.551615000	2.104937000
H	-3.700247000	-3.428949000	0.601837000
C	-5.031817000	-1.678183000	-1.128530000
H	-6.023805000	-2.160755000	-1.095777000
H	-4.359908000	-2.335884000	-1.695197000
H	-5.132134000	-0.737553000	-1.692175000
C	-4.155517000	2.103001000	-2.283258000
H	-4.561724000	1.591747000	-3.168753000
H	-4.358116000	3.180044000	-2.402369000
H	-4.696553000	1.739046000	-1.397971000
C	5.554267000	0.522968000	1.015012000
H	6.537656000	1.019047000	1.055286000
H	5.681124000	-0.440441000	0.494451000
H	5.240282000	0.303721000	2.048500000
C	5.031584000	1.678337000	-1.128698000
H	6.023565000	2.160934000	-1.096042000
H	4.359565000	2.336092000	-1.695174000
H	5.131836000	0.737782000	-1.692480000
C	4.405048000	2.732040000	1.077258000
H	5.385950000	3.235141000	1.134448000
H	4.053727000	2.551369000	2.104998000
H	3.700244000	3.428885000	0.602048000
C	1.835907000	-2.324994000	-3.332809000
H	2.195424000	-1.884294000	-4.275469000
H	0.780066000	-2.046999000	-3.199309000
H	1.903939000	-3.421919000	-3.419882000
C	-1.835369000	2.324561000	-3.332977000
H	-2.194734000	1.883876000	-4.275703000
H	-0.779641000	2.046291000	-3.199146000
H	-1.903083000	3.421498000	-3.420153000
C	-1.054896000	1.786989000	3.230676000
H	-0.464777000	1.454194000	4.098780000
H	-1.716501000	0.962173000	2.925213000

H	-1.680936000	2.637557000	3.547539000
C	1.054799000	-1.786056000	3.230613000
H	0.464698000	-1.453161000	4.098690000
H	1.716031000	-0.961102000	2.924718000
H	1.681217000	-2.636271000	3.547677000

COD

E(B3LYP-D3(BJ)) = -312.195795 E_h

E(M06-D3) = -311.900274 E_h

ΔΔG(353.15) = 0.140763 E_h

Lowest frequency = 84.91 cm^{-1}

H	2.733755000	0.301996000	0.667120000
H	2.436688000	-0.453406000	-0.885620000
H	1.768315000	-1.901877000	0.975202000
H	0.649162000	-0.710317000	1.601158000
H	1.810903000	1.836214000	-1.191378000
H	0.308033000	-2.624475000	-0.729144000
H	-0.308014000	2.624476000	-0.729165000
H	-1.810883000	-1.836222000	-1.191390000
H	-1.768334000	1.901892000	0.975145000
H	-0.649188000	0.710351000	1.601148000
H	-2.733750000	-0.302000000	0.667114000
H	-2.436695000	0.453385000	-0.885637000
C	1.919387000	-0.005629000	-0.017464000
C	1.079443000	-1.098450000	0.668656000
C	-1.079454000	1.098461000	0.668629000
C	-1.919388000	0.005622000	-0.017477000
C	1.209265000	1.230849000	-0.501886000
C	0.016179000	-1.696866000	-0.222155000
C	-0.016174000	1.696865000	-0.222172000
C	-1.209257000	-1.230855000	-0.501889000

Ni(COD)$_2$

E(B3LYP-D3(BJ)) = -2132.837449 E$_h$

E(M06-D3) = -2132.166979 E$_h$

ΔΔG(353.15) = 0.310590 E$_h$

Lowest frequency = 67.44 cm^{-1}

Ni	0.000018000	-0.000028000	-0.000006000
H	3.122941000	-2.396639000	0.268488000
H	1.646697000	-2.536559000	1.218195000
H	2.859422000	-0.921092000	2.424895000
H	3.516976000	-0.145577000	0.998097000
H	0.747321000	-2.360370000	-0.995376000
H	0.966903000	0.495289000	2.428543000
H	0.966938000	-0.495312000	-2.428525000
H	0.747258000	2.360347000	0.995400000
H	2.859403000	0.921128000	-2.424860000
H	3.516977000	0.145615000	-0.998071000
H	3.122914000	2.396662000	-0.268441000
H	1.646671000	2.536575000	-1.218149000
C	-2.212988000	1.843776000	0.574769000
C	-2.601949000	0.602065000	1.402530000
C	-2.601978000	-0.602036000	-1.402546000
C	-2.213017000	-1.843759000	-0.574807000
C	-1.350242000	1.525521000	-0.626240000
C	-1.495362000	-0.438943000	1.474167000
C	-1.495373000	0.438950000	-1.474202000
C	-1.350260000	-1.525520000	0.626199000
C	2.213009000	-1.843760000	0.574813000
C	2.601962000	-0.602028000	1.402547000
C	2.601953000	0.602054000	-1.402513000
C	2.212988000	1.843776000	-0.574771000
C	1.350245000	-1.525525000	-0.626188000
C	1.495349000	0.438952000	1.474191000
C	1.495355000	-0.438945000	-1.474158000

C	1.350226000	1.525528000	0.626228000
H	-3.122917000	2.396663000	0.268446000
H	-1.646662000	2.536578000	1.218135000
H	-2.859378000	0.921153000	2.424878000
H	-3.516985000	0.145631000	0.998111000
H	-0.747268000	2.360336000	-0.995411000
H	-0.966927000	-0.495306000	2.428524000
H	-0.966921000	0.495276000	-2.428552000
H	-0.747314000	-2.360354000	0.995373000
H	-2.859441000	-0.921108000	-2.424891000
H	-3.516992000	-0.145587000	-0.998096000
H	-3.122946000	-2.396646000	-0.268485000
H	-1.646697000	-2.536554000	-1.218186000

4-Ni
E(B3LYP-D3(BJ)) = -3344.479385 E_h
E(M06-D3) = -3342.745601 E_h
ΔΔG(353.15) = 0.929074 E_h
Lowest frequency = -21.33 cm^{-1}

B	-0.537159000	-0.721129000	-0.915339000
B	-0.832254000	-0.444201000	0.673215000
C	-0.022423000	-2.209382000	3.944810000
H	0.265217000	-2.156564000	4.989367000
C	0.760918000	-1.549441000	-3.099152000
H	0.915732000	-2.565129000	-3.495198000
H	1.620812000	-0.950846000	-3.433867000
H	-0.131954000	-1.147917000	-3.599896000
C	0.600106000	-1.562381000	-1.584323000
C	1.591361000	-2.165236000	-0.798705000
H	1.322942000	-2.380447000	0.240671000
C	2.830433000	-2.972930000	-1.209156000
C	3.744033000	-2.272579000	-2.231140000
H	4.690503000	-2.829576000	-2.332312000

H	3.977623000	-1.251314000	-1.907805000
H	3.293066000	-2.219744000	-3.230357000
C	2.409408000	-4.346202000	-1.780703000
H	3.296919000	-4.947082000	-2.043291000
H	1.798481000	-4.238624000	-2.688998000
H	1.821426000	-4.919299000	-1.050003000
C	3.655948000	-3.221465000	0.068411000
H	4.538780000	-3.845917000	-0.144187000
H	3.053233000	-3.736855000	0.834531000
H	3.999926000	-2.270643000	0.500312000
C	-1.394612000	0.190002000	-1.907831000
N	-1.066276000	1.317182000	-2.599350000
C	-2.153554000	1.780423000	-3.322519000
H	-2.112200000	2.677904000	-3.930622000
C	-3.180426000	0.918033000	-3.090075000
H	-4.200636000	0.921670000	-3.457397000
N	-2.697005000	-0.047174000	-2.225027000
C	0.260397000	1.960984000	-2.616705000
H	0.846064000	1.389113000	-1.873032000
C	0.168537000	3.417681000	-2.173341000
H	1.175233000	3.858228000	-2.160113000
H	-0.259886000	3.503633000	-1.170341000
H	-0.444020000	4.015549000	-2.867607000
C	0.902512000	1.848890000	-3.998862000
H	1.937331000	2.219942000	-3.952193000
H	0.360925000	2.458831000	-4.740683000
H	0.925570000	0.811962000	-4.352506000
C	-3.434374000	-1.227059000	-1.746816000
H	-2.954352000	-1.450833000	-0.783785000
C	-3.200864000	-2.409683000	-2.685217000
H	-3.721277000	-3.302713000	-2.305980000
H	-2.126559000	-2.636505000	-2.750218000
H	-3.578766000	-2.196350000	-3.698472000

C	-4.909663000	-0.912782000	-1.530992000
H	-5.387346000	-1.755821000	-1.010174000
H	-5.449059000	-0.768785000	-2.481579000
H	-5.036895000	-0.011768000	-0.914767000
C	-3.172991000	-0.020753000	1.809108000
H	-3.801448000	0.687777000	2.365572000
H	-2.858369000	-0.802235000	2.518529000
H	-3.832424000	-0.526828000	1.083768000
C	-1.972939000	0.599474000	1.105331000
C	-1.937738000	1.912895000	0.779158000
H	-1.013967000	2.244255000	0.297129000
C	-2.981547000	3.020054000	0.973869000
C	-2.487975000	4.286589000	0.250349000
H	-3.182740000	5.127416000	0.408362000
H	-2.408699000	4.114512000	-0.833803000
H	-1.495755000	4.592659000	0.619102000
C	-3.149430000	3.374860000	2.466981000
H	-3.854181000	4.214628000	2.594536000
H	-2.182737000	3.671992000	2.902770000
H	-3.530863000	2.528283000	3.054605000
C	-4.346837000	2.646249000	0.359444000
H	-5.052809000	3.491027000	0.435364000
H	-4.808326000	1.783953000	0.858392000
H	-4.228051000	2.396691000	-0.706805000
C	-0.478410000	-1.430190000	1.861977000
N	-0.126380000	-1.086663000	3.142406000
C	-0.311759000	-3.283232000	3.163234000
H	-0.334762000	-4.340669000	3.402695000
N	-0.585997000	-2.797533000	1.895749000
C	0.159701000	0.281513000	3.605341000
H	2.304179000	0.726255000	1.960211000
C	1.488987000	0.324269000	4.360916000
H	1.793750000	1.370820000	4.506547000

H	2.285094000	-0.186678000	3.802299000
H	1.407722000	-0.139311000	5.357582000
C	-0.984666000	0.832550000	4.453943000
H	-0.750886000	1.861598000	4.766170000
H	-1.132858000	0.227201000	5.363656000
H	-1.922270000	0.857558000	3.889129000
C	-1.117821000	-3.622241000	0.797563000
H	-0.909919000	-3.041845000	-0.110013000
C	-2.631172000	-3.781345000	0.949089000
H	-3.041064000	-4.330647000	0.087610000
H	-3.119040000	-2.798954000	0.999586000
H	-2.884668000	-4.340609000	1.864522000
C	-0.400624000	-4.965916000	0.727105000
H	-0.669217000	-5.476407000	-0.209272000
H	-0.683313000	-5.633196000	1.557926000
H	0.689111000	-4.832585000	0.740030000
H	3.075699000	3.326782000	-1.165914000
C	4.032716000	2.795950000	-1.084486000
H	4.615880000	3.096673000	-1.971748000
H	3.186392000	1.092399000	-2.086002000
C	3.786454000	1.268547000	-1.175902000
H	4.756100000	0.783607000	-1.379335000
C	2.798252000	3.511319000	1.801154000
H	2.792555000	3.191336000	2.859544000
H	2.414745000	4.551604000	1.822484000
C	1.806802000	2.616109000	1.030117000
H	1.618665000	3.026285000	0.033408000
H	0.834307000	2.677849000	1.547310000
C	4.764610000	3.239099000	0.153625000
H	5.856414000	3.299098000	0.064528000
Ni	1.353273000	-0.147715000	-0.178402000
C	3.174829000	0.588350000	0.047548000
H	3.835984000	-0.166428000	0.486415000

C	4.239183000	3.530136000	1.352286000
H	4.946724000	3.834529000	2.134195000
H	0.239020000	0.865930000	2.681080000
C	2.243678000	1.169104000	0.960860000

TS1

E(B3LYP-D3(BJ)) = -3344.424647 E_h

E(M06-D3) = -3342.683610 E_h

ΔΔG(353.15) = 0.925944 E_h

Lowest frequency = -63.76 cm^{-1}

B	-0.099073000	-0.641155000	1.141954000
B	0.554347000	-0.524435000	-0.337296000
C	-0.185607000	-1.659584000	-4.267046000
H	0.114599000	-1.574449000	-5.306500000
C	-1.721364000	-0.860447000	3.219930000
H	-2.431177000	-1.621435000	3.577065000
H	-2.100098000	0.111307000	3.578609000
H	-0.768185000	-1.040579000	3.738581000
C	-1.547192000	-0.880804000	1.705371000
C	-2.653102000	-1.052502000	0.907830000
H	-2.482253000	-1.141743000	-0.175236000
C	-4.137411000	-1.229121000	1.251739000
C	-4.674335000	-0.281787000	2.345300000
H	-5.773019000	-0.361164000	2.401834000
H	-4.422009000	0.763020000	2.120887000
H	-4.281754000	-0.518735000	3.341697000
C	-4.402807000	-2.687863000	1.691897000
H	-5.471065000	-2.843972000	1.923436000
H	-3.821502000	-2.944777000	2.590453000
H	-4.120494000	-3.394778000	0.898884000
C	-4.953924000	-0.950623000	-0.026171000
H	-6.027177000	-1.135196000	0.143241000
H	-4.627823000	-1.593205000	-0.858195000

H	-4.830194000	0.093726000	-0.349337000
C	1.088412000	-0.492106000	2.178836000
N	1.496357000	0.604741000	2.884203000
C	2.651502000	0.331526000	3.597816000
H	3.149635000	1.072641000	4.214438000
C	2.973807000	-0.966726000	3.343108000
H	3.804659000	-1.566825000	3.697913000
N	2.010049000	-1.455406000	2.478597000
C	0.807500000	1.901702000	2.859441000
H	0.046232000	1.767004000	2.076737000
C	1.758445000	3.022907000	2.446603000
H	1.199946000	3.965686000	2.355059000
H	2.229335000	2.805875000	1.480156000
H	2.554058000	3.181012000	3.192505000
C	0.125402000	2.187684000	4.195665000
H	-0.472426000	3.108076000	4.118048000
H	0.865470000	2.330877000	5.000380000
H	-0.543259000	1.365568000	4.480952000
C	1.911417000	-2.837616000	1.983828000
H	1.384638000	-2.724322000	1.025633000
C	1.034400000	-3.673462000	2.914119000
H	0.936581000	-4.697657000	2.521747000
H	0.028746000	-3.234739000	2.983828000
H	1.467012000	-3.732599000	3.926374000
C	3.287556000	-3.447008000	1.747845000
H	3.172960000	-4.408593000	1.225659000
H	3.822707000	-3.650480000	2.690187000
H	3.910098000	-2.790015000	1.125555000
C	2.540176000	-1.719980000	-1.660099000
H	3.482478000	-1.530727000	-2.191601000
H	1.805548000	-2.052198000	-2.406222000
H	2.712824000	-2.584891000	-0.997086000
C	2.027606000	-0.531481000	-0.870261000

C	2.823366000	0.502256000	-0.494373000
H	2.310575000	1.308649000	0.038456000
C	4.332362000	0.726486000	-0.644320000
C	4.703826000	1.983819000	0.164453000
H	5.780474000	2.204433000	0.082956000
H	4.464094000	1.848053000	1.230862000
H	4.148760000	2.864394000	-0.197543000
C	4.731441000	0.975542000	-2.113814000
H	5.807033000	1.209558000	-2.191547000
H	4.167064000	1.823490000	-2.531194000
H	4.535912000	0.099869000	-2.748839000
C	5.147102000	-0.450981000	-0.071437000
H	6.228766000	-0.241776000	-0.131926000
H	4.959078000	-1.388118000	-0.612401000
H	4.890493000	-0.611991000	0.987066000
C	-0.639098000	-0.934897000	-2.143118000
N	-0.193010000	-0.581919000	-3.400645000
C	-0.601812000	-2.737775000	-3.555460000
H	-0.722855000	-3.773750000	-3.854275000
N	-0.852404000	-2.296449000	-2.265140000
C	0.240292000	0.762125000	-3.790844000
H	-1.632779000	2.236680000	-2.370625000
C	-0.765234000	1.392790000	-4.754140000
H	-0.508512000	2.447369000	-4.937156000
H	-1.782543000	1.349982000	-4.339497000
H	-0.768458000	0.872243000	-5.726095000
C	1.660307000	0.744369000	-4.352268000
H	1.992931000	1.775531000	-4.545957000
H	1.723552000	0.191533000	-5.303627000
H	2.353654000	0.294351000	-3.632032000
C	-1.214555000	-3.229470000	-1.185683000
H	-1.263687000	-2.623240000	-0.276059000
C	-0.124979000	-4.283302000	-0.984126000

H	-0.355224000	-4.879365000	-0.088027000
H	0.851516000	-3.805384000	-0.839435000
H	-0.050484000	-4.975713000	-1.838342000
C	-2.575687000	-3.863247000	-1.465439000
H	-2.870457000	-4.507730000	-0.624606000
H	-2.554080000	-4.486212000	-2.374487000
H	-3.347664000	-3.092831000	-1.594299000
H	-0.849146000	3.790056000	1.445221000
C	-1.939419000	3.851841000	1.348531000
H	-2.309749000	4.134816000	2.348458000
H	-2.074548000	1.729000000	1.723304000
C	-2.516093000	2.451820000	1.019049000
H	-3.591377000	2.468203000	1.256794000
C	-0.401883000	4.605316000	-1.338515000
H	-0.463160000	4.709282000	-2.436730000
H	0.491133000	5.194436000	-1.049545000
C	-0.150722000	3.117190000	-1.013474000
H	0.165114000	2.981901000	0.025829000
H	0.717325000	2.787375000	-1.608221000
C	-2.295809000	4.937408000	0.368872000
H	-3.200321000	5.514674000	0.595693000
Ni	-1.141635000	0.317792000	-0.761396000
C	-2.383280000	1.962654000	-0.412525000
H	-3.332992000	1.675680000	-0.872740000
C	-1.638651000	5.248842000	-0.757121000
H	-2.040548000	6.079257000	-1.350542000
H	0.248622000	1.317986000	-2.850438000
C	-1.350314000	2.245938000	-1.312903000

(4-Ni)b

E(B3LYP-D3(BJ)) = -3344.448869 E_h

E(M06-D3) = -3342.709631 E_h

$\Delta\Delta$G(353.15) = 0.919635 E_h

Lowest frequency = 14.10 cm^{-1}

B	-0.282800000	-1.210661000	0.010688000
B	-0.887915000	0.227677000	0.463289000
C	0.391726000	4.539240000	0.724794000
H	-0.008275000	5.512337000	0.457554000
C	1.117865000	-3.472698000	-0.025129000
H	1.172279000	-4.306753000	0.692392000
H	2.068981000	-3.469263000	-0.575744000
H	0.339607000	-3.720057000	-0.760582000
C	0.836865000	-2.135016000	0.651288000
C	1.504027000	-1.765039000	1.777328000
H	1.160533000	-0.837629000	2.247371000
C	2.612436000	-2.471149000	2.560257000
C	3.739286000	-3.038256000	1.673987000
H	4.579731000	-3.379633000	2.301086000
H	4.121415000	-2.268074000	0.986741000
H	3.412885000	-3.895387000	1.072722000
C	2.002291000	-3.604557000	3.415021000
H	2.776563000	-4.104919000	4.022336000
H	1.522657000	-4.368348000	2.784722000
H	1.236428000	-3.205916000	4.099145000
C	3.252094000	-1.444535000	3.514309000
H	4.018416000	-1.917857000	4.149051000
H	2.494653000	-0.994579000	4.174461000
H	3.737322000	-0.632748000	2.949474000
C	-1.451487000	-1.904275000	-0.767186000
N	-2.039982000	-1.568609000	-1.962050000
C	-3.180950000	-2.327402000	-2.176992000
H	-3.798186000	-2.213627000	-3.062118000

C	-3.306512000	-3.167470000	-1.115627000
H	-4.059998000	-3.917339000	-0.898047000
N	-2.243250000	-2.910077000	-0.265887000
C	-1.518017000	-0.577338000	-2.911208000
H	-0.593809000	-0.227171000	-2.433689000
C	-2.466942000	0.611626000	-3.045136000
H	-2.014626000	1.374055000	-3.697767000
H	-2.662152000	1.064357000	-2.063920000
H	-3.432523000	0.319434000	-3.490866000
C	-1.186743000	-1.230521000	-4.252226000
H	-0.675409000	-0.503921000	-4.901878000
H	-2.090978000	-1.574202000	-4.781582000
H	-0.517746000	-2.092356000	-4.113509000
C	-2.109681000	-3.462741000	1.091472000
H	-1.111600000	-3.157022000	1.425112000
C	-2.182141000	-4.987062000	1.071225000
H	-1.974818000	-5.380296000	2.077729000
H	-1.439023000	-5.407552000	0.377780000
H	-3.180202000	-5.350109000	0.775715000
C	-3.132963000	-2.814657000	2.023890000
H	-2.985151000	-3.172158000	3.054627000
H	-4.163941000	-3.061881000	1.720997000
H	-3.016316000	-1.722206000	2.007445000
C	-1.979106000	1.936093000	2.094472000
H	-2.854752000	2.043861000	2.747539000
H	-1.743722000	2.929467000	1.680933000
H	-1.124250000	1.666654000	2.729426000
C	-2.171916000	0.907273000	0.995325000
C	-3.395505000	0.557830000	0.511779000
H	-3.407733000	-0.239656000	-0.235972000
C	-4.800278000	1.061524000	0.850934000
C	-5.771254000	0.446798000	-0.176100000
H	-6.809898000	0.758323000	0.020628000

H	-5.731468000	-0.653958000	-0.144254000
H	-5.508095000	0.759200000	-1.199757000
C	-4.926840000	2.595021000	0.760382000
H	-5.958296000	2.911080000	0.990818000
H	-4.688145000	2.944098000	-0.255772000
H	-4.256326000	3.111851000	1.459598000
C	-5.229735000	0.585023000	2.256722000
H	-6.262938000	0.903089000	2.479043000
H	-4.576660000	0.989402000	3.043328000
H	-5.189840000	-0.512765000	2.323469000
C	0.806808000	2.305870000	0.501527000
N	0.204867000	3.406112000	-0.054758000
C	1.125168000	4.152834000	1.801309000
H	1.484600000	4.725132000	2.650766000
N	1.373333000	2.792436000	1.647932000
C	-0.491973000	3.363661000	-1.344153000
H	1.223476000	0.585948000	-2.895004000
C	0.346116000	4.020716000	-2.439754000
H	-0.155122000	3.917989000	-3.414783000
H	1.336080000	3.547919000	-2.509282000
H	0.488775000	5.096546000	-2.244972000
C	-1.896882000	3.948927000	-1.239462000
H	-2.423196000	3.826024000	-2.197874000
H	-1.879234000	5.026698000	-1.008224000
H	-2.475663000	3.429351000	-0.465894000
C	2.191746000	1.991675000	2.561957000
H	2.226060000	1.000296000	2.092368000
C	1.534033000	1.867657000	3.935765000
H	2.175398000	1.282210000	4.609818000
H	0.561444000	1.360888000	3.863524000
H	1.374608000	2.856520000	4.395195000
C	3.612530000	2.548435000	2.648938000
H	4.250812000	1.861029000	3.224338000

H	3.634191000	3.527817000	3.153982000
H	4.046400000	2.665277000	1.646284000
H	4.527433000	-1.401500000	-1.130890000
C	4.844633000	-0.357464000	-1.257167000
H	5.809769000	-0.276698000	-0.729991000
H	3.545335000	0.069242000	0.403054000
C	3.825132000	0.572464000	-0.535535000
H	4.345764000	1.496393000	-0.237506000
C	3.067487000	-1.403343000	-3.676581000
H	2.444167000	-1.157428000	-4.555212000
H	3.372902000	-2.457303000	-3.833715000
C	2.173173000	-1.319656000	-2.419900000
H	2.664092000	-1.786269000	-1.561206000
H	1.274459000	-1.928707000	-2.603683000
C	5.049558000	-0.070436000	-2.719783000
H	5.889565000	0.586629000	-2.975705000
Ni	0.807361000	0.635887000	-0.390640000
C	2.590188000	0.979089000	-1.329971000
H	2.603515000	2.024176000	-1.651826000
C	4.291168000	-0.517342000	-3.731368000
H	4.588117000	-0.212967000	-4.742689000
H	-0.573250000	2.285582000	-1.553146000
C	1.787534000	0.099270000	-2.084762000

TS2
E(B3LYP-D3(BJ)) = -3344.420729 E_h
E(M06-D3) = -3342.686211 E_h
ΔΔG(353.15) = 0.922980 E_h
Lowest frequency = -78.75 cm^{-1}

B	-0.809406000	-0.961612000	0.548550000
B	-0.802580000	0.706795000	0.695295000
C	1.634294000	3.265897000	-2.562350000
H	1.394022000	3.714774000	-3.520305000

C	0.796459000	-2.282761000	2.355735000
H	0.420420000	-2.403699000	3.390086000
H	1.894362000	-2.231689000	2.444716000
H	0.559739000	-3.215223000	1.816512000
C	0.162599000	-1.077162000	1.695498000
C	-0.140256000	0.224554000	2.409578000
H	-1.115655000	0.154428000	2.928497000
C	0.821613000	0.918806000	3.409922000
C	2.284790000	0.540534000	3.148121000
H	2.986585000	1.272571000	3.581574000
H	2.470138000	0.466660000	2.065877000
H	2.527732000	-0.442842000	3.574933000
C	0.461444000	0.482528000	4.845404000
H	1.135453000	0.942409000	5.589618000
H	0.533457000	-0.611333000	4.957025000
H	-0.569993000	0.778007000	5.098254000
C	0.636870000	2.438553000	3.314513000
H	1.281308000	2.978771000	4.030758000
H	-0.409326000	2.710628000	3.525787000
H	0.861627000	2.789767000	2.301242000
C	-1.749577000	-2.068950000	0.010273000
N	-2.102820000	-2.448569000	-1.255452000
C	-3.121356000	-3.391326000	-1.221206000
H	-3.542606000	-3.830418000	-2.119409000
C	-3.408210000	-3.613864000	0.088261000
H	-4.133525000	-4.279298000	0.543541000
N	-2.559203000	-2.813048000	0.834258000
C	-1.591111000	-1.825884000	-2.478199000
H	-0.722892000	-1.243214000	-2.143061000
C	-2.651160000	-0.879654000	-3.044261000
H	-2.295178000	-0.407295000	-3.970104000
H	-2.890904000	-0.092008000	-2.315874000
H	-3.575113000	-1.429728000	-3.283697000

C	-1.129222000	-2.874638000	-3.488259000
H	-0.634115000	-2.379577000	-4.336527000
H	-1.975106000	-3.454242000	-3.891288000
H	-0.413451000	-3.574950000	-3.035013000
C	-2.678236000	-2.581991000	2.285374000
H	-1.695919000	-2.208381000	2.589489000
C	-2.970106000	-3.880627000	3.029178000
H	-2.916136000	-3.697955000	4.112428000
H	-2.233454000	-4.658188000	2.776177000
H	-3.979352000	-4.268755000	2.814870000
C	-3.704801000	-1.481320000	2.553995000
H	-3.750392000	-1.264002000	3.631924000
H	-4.710744000	-1.782593000	2.217395000
H	-3.415533000	-0.558540000	2.030368000
C	-1.295922000	3.338647000	0.492197000
H	-1.910562000	4.070053000	-0.051329000
H	-0.259473000	3.440607000	0.138402000
H	-1.288228000	3.640934000	1.551837000
C	-1.767849000	1.906784000	0.352128000
C	-3.043956000	1.578263000	0.028931000
H	-3.242205000	0.501685000	-0.030710000
C	-4.298619000	2.434734000	-0.178488000
C	-5.496666000	1.474227000	-0.307556000
H	-6.439174000	2.027083000	-0.452141000
H	-5.600091000	0.852540000	0.596401000
H	-5.365141000	0.794884000	-1.165969000
C	-4.230702000	3.280977000	-1.465949000
H	-5.165994000	3.849464000	-1.606742000
H	-4.088740000	2.639994000	-2.349480000
H	-3.403550000	4.004491000	-1.440212000
C	-4.551969000	3.353792000	1.034056000
H	-5.505338000	3.897523000	0.918429000
H	-3.754333000	4.098303000	1.159263000

H	-4.604370000	2.763107000	1.962035000
C	1.482916000	1.746831000	-0.854376000
N	1.010983000	2.121838000	-2.086767000
C	2.522012000	3.643952000	-1.610484000
H	3.203053000	4.488045000	-1.576424000
N	2.419022000	2.719337000	-0.582156000
C	-0.045016000	1.430357000	-2.828140000
H	1.786411000	-0.942732000	-1.978292000
C	0.547150000	0.679658000	-4.022559000
H	-0.238109000	0.139686000	-4.571061000
H	1.306853000	-0.046454000	-3.700088000
H	1.024199000	1.378305000	-4.728581000
C	-1.137200000	2.413658000	-3.243491000
H	-1.984378000	1.872777000	-3.685566000
H	-0.776683000	3.134010000	-3.995278000
H	-1.507857000	2.962826000	-2.370581000
C	3.217496000	2.823137000	0.642777000
H	2.812004000	2.053232000	1.300637000
C	3.027270000	4.188527000	1.305375000
H	3.480931000	4.181563000	2.307557000
H	1.961499000	4.430079000	1.415786000
H	3.508583000	4.992950000	0.726189000
C	4.692916000	2.522101000	0.377512000
H	5.254111000	2.537303000	1.324202000
H	5.141337000	3.273610000	-0.292628000
H	4.821672000	1.532353000	-0.079891000
H	3.499988000	-3.455092000	0.954732000
C	4.367628000	-2.898851000	0.577259000
H	5.184378000	-3.114949000	1.285362000
H	3.714922000	-1.164967000	1.668959000
C	4.081819000	-1.378314000	0.653535000
H	5.044701000	-0.844642000	0.580027000
C	2.451048000	-3.744864000	-1.876160000

H	2.171087000	-3.457099000	-2.906546000
H	2.083334000	-4.784356000	-1.765344000
C	1.687468000	-2.836874000	-0.893590000
H	1.811269000	-3.184106000	0.133244000
H	0.613684000	-2.936413000	-1.099626000
C	4.763507000	-3.398481000	-0.788727000
H	5.842433000	-3.461399000	-0.975954000
Ni	1.065829000	0.081676000	0.122460000
C	3.166049000	-0.790510000	-0.400530000
H	3.550301000	0.109614000	-0.881442000
C	3.958264000	-3.746108000	-1.803109000
H	4.449379000	-4.090584000	-2.721832000
H	-0.461009000	0.725559000	-2.097095000
C	2.095740000	-1.393273000	-1.029407000

(4-Ni)c

E(B3LYP-D3(BJ)) = -3344.426616 E_h

E(M06-D3) = -3342.692429 E_h

ΔΔG(353.15) = 0.923938 E_h

Lowest frequency = 16.08 cm^{-1}

B	0.793134000	0.959998000	0.502074000
B	0.793991000	-0.700763000	0.845679000
C	-1.492007000	-3.626717000	-2.125989000
H	-1.124093000	-4.268094000	-2.919151000
C	-0.764504000	2.273128000	2.367442000
H	-0.361505000	2.365793000	3.394239000
H	-1.859806000	2.209786000	2.484683000
H	-0.548879000	3.222360000	1.849446000
C	-0.143315000	1.087169000	1.665298000
C	0.253495000	-0.224285000	2.361848000
H	1.184177000	-0.039667000	2.943489000
C	-0.695268000	-0.950189000	3.372690000
C	-2.160837000	-0.615387000	3.083096000

H	-2.854655000	-1.345527000	3.530378000
H	-2.317008000	-0.593811000	1.994207000
H	-2.431766000	0.379586000	3.462450000
C	-0.365081000	-0.493371000	4.806470000
H	-1.041459000	-0.958343000	5.545196000
H	-0.459204000	0.599836000	4.907098000
H	0.668173000	-0.765789000	5.078039000
C	-0.482074000	-2.465493000	3.293906000
H	-1.098741000	-3.002744000	4.036243000
H	0.572166000	-2.718244000	3.485342000
H	-0.732396000	-2.840735000	2.292978000
C	1.724925000	2.019254000	-0.121955000
N	2.074489000	2.277617000	-1.419922000
C	3.098559000	3.213150000	-1.477501000
H	3.517534000	3.565166000	-2.414399000
C	3.395886000	3.552112000	-0.195822000
H	4.129899000	4.249934000	0.192067000
N	2.545735000	2.830730000	0.626606000
C	1.555485000	1.543574000	-2.575485000
H	0.732997000	0.942411000	-2.166166000
C	2.645049000	0.613946000	-3.113843000
H	2.282483000	0.053288000	-3.986133000
H	2.952559000	-0.101804000	-2.338457000
H	3.529257000	1.188695000	-3.431997000
C	1.000226000	2.493528000	-3.634786000
H	0.524292000	1.916908000	-4.441344000
H	1.795083000	3.106872000	-4.088906000
H	0.246305000	3.167522000	-3.204180000
C	2.671674000	2.738156000	2.091268000
H	1.693709000	2.387435000	2.433181000
C	2.953219000	4.104134000	2.708468000
H	2.905045000	4.024650000	3.804435000
H	2.208637000	4.847580000	2.385699000

H	3.957819000	4.479328000	2.453502000
C	3.709400000	1.678154000	2.461470000
H	3.757239000	1.563479000	3.555004000
H	4.712088000	1.956712000	2.096923000
H	3.430968000	0.707264000	2.027222000
C	1.429303000	-3.331836000	0.639459000
H	2.053056000	-4.018969000	0.049301000
H	0.381158000	-3.501554000	0.353697000
H	1.515279000	-3.644271000	1.692085000
C	1.804882000	-1.873285000	0.475863000
C	3.056560000	-1.482034000	0.129888000
H	3.190279000	-0.398725000	0.035349000
C	4.364713000	-2.263461000	-0.050331000
C	5.500922000	-1.230002000	-0.180174000
H	6.478388000	-1.723697000	-0.305634000
H	5.553364000	-0.589897000	0.715078000
H	5.337243000	-0.572330000	-1.049818000
C	4.369385000	-3.131308000	-1.324586000
H	5.345418000	-3.630694000	-1.450206000
H	4.187174000	-2.516657000	-2.219454000
H	3.598672000	-3.913933000	-1.292239000
C	4.662783000	-3.147468000	1.177566000
H	5.653638000	-3.624058000	1.083171000
H	3.918292000	-3.945369000	1.300240000
H	4.658765000	-2.543575000	2.098487000
C	-1.529603000	-1.799026000	-0.745079000
N	-0.894090000	-2.422146000	-1.789554000
C	-2.540668000	-3.784699000	-1.281863000
H	-3.259012000	-4.593435000	-1.194286000
N	-2.563526000	-2.666051000	-0.461161000
C	0.165207000	-1.804531000	-2.589737000
H	-1.634314000	1.104295000	-2.029042000
C	-0.467369000	-1.077513000	-3.780031000

H	0.299379000	-0.605026000	-4.409768000
H	-1.167495000	-0.301850000	-3.439529000
H	-1.027029000	-1.787566000	-4.409840000
C	1.200054000	-2.832955000	-3.033281000
H	2.063900000	-2.320553000	-3.476839000
H	0.798657000	-3.520215000	-3.795461000
H	1.559099000	-3.417498000	-2.178721000
C	-3.658213000	-2.431608000	0.487641000
H	-3.434673000	-1.464085000	0.941166000
C	-3.684264000	-3.497329000	1.581436000
H	-4.454311000	-3.251064000	2.327909000
H	-2.716003000	-3.555634000	2.093314000
H	-3.922798000	-4.491353000	1.169398000
C	-4.998350000	-2.325282000	-0.244019000
H	-5.788212000	-2.035204000	0.465311000
H	-5.292095000	-3.285298000	-0.697592000
H	-4.957110000	-1.568915000	-1.040929000
H	-3.490025000	3.342182000	1.030377000
C	-4.354162000	2.835005000	0.583012000
H	-5.181792000	2.981768000	1.296077000
H	-3.752105000	0.983938000	1.500735000
C	-4.077985000	1.314766000	0.501664000
H	-5.037462000	0.799934000	0.323582000
C	-2.402619000	3.906163000	-1.770034000
H	-2.121112000	3.682913000	-2.816079000
H	-2.035364000	4.936773000	-1.594936000
C	-1.642635000	2.941041000	-0.841246000
H	-1.792634000	3.216557000	0.203358000
H	-0.565729000	3.068647000	-1.019566000
C	-4.726780000	3.472448000	-0.731869000
H	-5.802996000	3.565471000	-0.922161000
Ni	-1.070491000	-0.062560000	0.078446000
C	-3.113164000	0.842536000	-0.566687000

H	-3.453587000	-0.024526000	-1.132674000
C	-3.909561000	3.908381000	-1.701781000
H	-4.392115000	4.344302000	-2.585670000
H	0.640665000	-1.089573000	-1.905904000
C	-2.024183000	1.505024000	-1.085790000

(4-Ni)d

E(B3LYP-D3(BJ)) = -3032.258001 E_h

E(M06-D3) = -3030.809423 E_h

ΔΔG(353.15) = 0.749153 E_h

Lowest frequency = 14.48 cm^{-1}

B	-1.105174000	-1.049988000	-0.381656000
B	0.942839000	-1.205817000	-0.566081000
C	0.792871000	4.472612000	1.289203000
H	0.585425000	5.237070000	2.031801000
C	-0.293421000	-0.777418000	-3.060138000
H	0.153614000	-1.657189000	-3.559759000
H	0.230282000	0.105065000	-3.474213000
H	-1.339343000	-0.715323000	-3.410790000
C	-0.176836000	-0.869194000	-1.564740000
C	-0.028758000	-1.677754000	0.664135000
H	-0.041035000	-2.785462000	0.613527000
C	0.054937000	-1.270453000	2.168135000
C	1.093107000	-0.161579000	2.411141000
H	1.095474000	0.162419000	3.467407000
H	0.879356000	0.707607000	1.772210000
H	2.105771000	-0.503174000	2.157575000
C	0.441885000	-2.491902000	3.021438000
H	0.516806000	-2.233820000	4.092150000
H	1.413084000	-2.904093000	2.705102000
H	-0.305562000	-3.297991000	2.923249000
C	-1.316151000	-0.763919000	2.656478000
H	-1.292838000	-0.470111000	3.720433000

H	-2.092664000	-1.537810000	2.541499000
H	-1.633524000	0.109212000	2.065521000
C	-2.682347000	-1.184431000	-0.438338000
N	-3.590889000	-0.305782000	-0.945996000
C	-4.855356000	-0.870918000	-0.964047000
H	-5.729209000	-0.336531000	-1.322131000
C	-4.735775000	-2.126063000	-0.452312000
H	-5.484368000	-2.893824000	-0.293570000
N	-3.402112000	-2.299407000	-0.121821000
C	-3.300038000	1.078640000	-1.352203000
H	-2.214736000	1.206493000	-1.148738000
C	-4.087031000	2.054225000	-0.482589000
H	-3.778497000	3.084147000	-0.709036000
H	-3.902966000	1.865430000	0.584747000
H	-5.172163000	1.981927000	-0.663329000
C	-3.554389000	1.282299000	-2.841882000
H	-3.244132000	2.297371000	-3.132222000
H	-4.621898000	1.170000000	-3.095440000
H	-2.973408000	0.566144000	-3.438281000
C	-2.792032000	-3.558389000	0.349030000
H	-1.961853000	-3.244775000	0.992128000
C	-2.209518000	-4.329423000	-0.834409000
H	-1.693836000	-5.233915000	-0.478067000
H	-1.479458000	-3.705688000	-1.370942000
H	-3.002959000	-4.634102000	-1.536388000
C	-3.776505000	-4.377092000	1.175763000
H	-3.243918000	-5.226347000	1.627980000
H	-4.592685000	-4.793439000	0.563524000
H	-4.214448000	-3.778705000	1.989134000
C	3.035384000	-0.992454000	-2.147452000
H	4.121201000	-0.826735000	-2.172924000
H	2.542780000	-0.076163000	-2.503249000
H	2.803135000	-1.763620000	-2.902891000

C	2.488424000	-1.405188000	-0.796220000
C	3.211934000	-2.040346000	0.153956000
H	2.656895000	-2.307827000	1.058440000
C	4.677177000	-2.475653000	0.186871000
C	4.902276000	-3.286272000	1.477017000
H	5.948489000	-3.623394000	1.558410000
H	4.253863000	-4.177069000	1.499512000
H	4.668567000	-2.680939000	2.367807000
C	5.614097000	-1.249932000	0.219503000
H	6.667126000	-1.568130000	0.306715000
H	5.380772000	-0.603173000	1.079747000
H	5.521739000	-0.639971000	-0.689696000
C	5.029048000	-3.375181000	-1.015177000
H	6.067147000	-3.740066000	-0.933542000
H	4.934669000	-2.843041000	-1.971133000
H	4.361875000	-4.250732000	-1.052708000
C	0.425316000	2.583769000	0.056187000
N	-0.065009000	3.411554000	1.035265000
C	1.852056000	4.324179000	0.448974000
H	2.742052000	4.933596000	0.324872000
N	1.609921000	3.180935000	-0.294342000
C	-1.325264000	3.179978000	1.741655000
C	-2.290141000	4.345556000	1.523844000
H	-3.276319000	4.112358000	1.952978000
H	-2.416239000	4.554983000	0.451738000
H	-1.923907000	5.263986000	2.010453000
C	-1.083734000	2.892656000	3.223176000
H	-2.027962000	2.611996000	3.714231000
H	-0.683458000	3.779034000	3.741938000
H	-0.373167000	2.065637000	3.345339000
C	2.540443000	2.596052000	-1.264914000
H	1.966874000	1.766898000	-1.707603000
C	3.761288000	2.015153000	-0.555707000

H	4.442475000	1.556411000	-1.286272000
H	3.453993000	1.236118000	0.156056000
H	4.318552000	2.796846000	-0.013728000
C	2.896393000	3.596446000	-2.361923000
H	3.504169000	3.102540000	-3.135077000
H	3.483514000	4.443347000	-1.969863000
H	1.987796000	3.995793000	-2.837390000
Ni	-0.116818000	0.878813000	-0.594725000
H	-1.727772000	2.273846000	1.265264000

6

E(B3LYP-D3(BJ)) = -3032.291963 E_h

E(M06-D3) = -3030.851901 E_h

ΔΔG(353.15) = 0.753487 E_h

Lowest frequency = 21.32 cm^{-1}

Ni	-0.498504000	-0.157456000	-0.449530000
B	1.491294000	-0.451002000	-1.463347000
B	0.021544000	-0.716131000	-2.605565000
H	-0.388844000	-1.002026000	-3.712454000
C	0.477651000	0.607028000	-1.982411000
C	0.616598000	2.027881000	-2.438158000
H	1.237611000	2.089444000	-3.350104000
H	1.106233000	2.670537000	-1.685564000
H	-0.349327000	2.503125000	-2.680387000
C	0.478540000	-1.614692000	-1.439722000
C	0.429952000	-3.124531000	-1.314837000
C	-1.037449000	-3.596176000	-1.312037000
H	-1.110795000	-4.696341000	-1.260278000
H	-1.553109000	-3.257395000	-2.223956000
H	-1.568882000	-3.171933000	-0.447466000
C	1.107512000	-3.641637000	-0.038385000
H	1.135477000	-4.744572000	-0.020009000
H	0.556391000	-3.306318000	0.850039000

H	2.142103000	-3.274822000	0.038600000
C	1.135623000	-3.747987000	-2.538837000
H	1.082900000	-4.850023000	-2.504813000
H	2.195790000	-3.459182000	-2.571996000
H	0.665515000	-3.408782000	-3.473736000
C	3.958813000	-1.377646000	-1.757532000
H	5.030343000	-1.146024000	-1.704656000
H	3.717001000	-1.603640000	-2.809440000
H	3.790486000	-2.317475000	-1.204312000
C	3.047542000	-0.292129000	-1.236099000
C	3.451794000	0.803960000	-0.558149000
H	2.652195000	1.499232000	-0.272110000
C	4.846359000	1.263982000	-0.126241000
C	5.716727000	1.590899000	-1.358447000
H	6.700497000	1.982977000	-1.048107000
H	5.227361000	2.351533000	-1.986453000
H	5.888263000	0.704892000	-1.985329000
C	5.553589000	0.211478000	0.752015000
H	6.536902000	0.582044000	1.087782000
H	5.715608000	-0.733021000	0.215603000
H	4.955591000	-0.015619000	1.648484000
C	4.689634000	2.554619000	0.700474000
H	5.668826000	2.937142000	1.031077000
H	4.073178000	2.379856000	1.596646000
H	4.199281000	3.342927000	0.106704000
C	-1.660890000	1.359257000	-0.380230000
N	-1.602372000	2.503840000	0.356700000
C	-2.653418000	3.361904000	0.058508000
H	-2.785395000	4.325913000	0.539326000
C	-3.392113000	2.743635000	-0.901515000
H	-4.288569000	3.070973000	-1.418538000
N	-2.778034000	1.524397000	-1.155304000
C	-0.535772000	2.755110000	1.327817000

H	0.219657000	2.002976000	1.063973000
C	-1.024060000	2.493245000	2.751780000
H	-0.207076000	2.647237000	3.472390000
H	-1.381230000	1.458687000	2.855129000
H	-1.847245000	3.175901000	3.018920000
C	0.072675000	4.142496000	1.143383000
H	0.964499000	4.243638000	1.779987000
H	-0.629461000	4.943211000	1.427330000
H	0.376444000	4.296715000	0.097683000
C	-3.206570000	0.573050000	-2.193788000
H	-2.590895000	-0.320416000	-2.012753000
C	-2.883426000	1.105971000	-3.588258000
H	-3.165326000	0.361974000	-4.347953000
H	-1.808630000	1.298695000	-3.686971000
H	-3.437518000	2.036405000	-3.795293000
C	-4.683769000	0.211826000	-2.043501000
H	-4.945614000	-0.575980000	-2.765192000
H	-5.338058000	1.075010000	-2.246522000
H	-4.910829000	-0.158311000	-1.034634000
C	-0.833817000	-0.953532000	1.270370000
N	-2.030410000	-1.236671000	1.874036000
C	-1.870693000	-1.566247000	3.212885000
H	-2.700651000	-1.829263000	3.861086000
C	-0.538376000	-1.499848000	3.467706000
H	0.012336000	-1.693143000	4.382256000
N	0.081352000	-1.136226000	2.280430000
C	-3.325447000	-1.164536000	1.194575000
H	-3.060725000	-0.971591000	0.147970000
C	-4.147283000	0.007375000	1.732846000
H	-5.103012000	0.089197000	1.194194000
H	-3.603535000	0.954317000	1.613263000
H	-4.377598000	-0.128654000	2.801838000
C	-4.069054000	-2.496916000	1.275683000

H	-4.985429000	-2.452633000	0.667579000
H	-4.367433000	-2.736457000	2.309026000
H	-3.442558000	-3.316756000	0.895912000
C	1.536881000	-0.946116000	2.155564000
H	1.733060000	-0.951377000	1.076709000
C	2.310689000	-2.086110000	2.819663000
H	3.367581000	-2.022153000	2.521511000
H	1.929900000	-3.069307000	2.517128000
H	2.274781000	-2.018450000	3.919280000
C	1.969101000	0.401021000	2.731706000
H	3.061783000	0.496268000	2.674655000
H	1.669610000	0.494636000	3.788233000
H	1.535439000	1.233425000	2.168107000

References

[1] N. Kuhn, T. Kratz, *Synthesis* **1993**, 561-562.

[2] H. C. Brown, S. K. Gupta, *J. Am. Chem. Soc.* **1975**, *97*, 5249-5255.

[3] L. E. Crascall, J. L. Spencer, R. A. Doyle, R. J. Angelici, *Inorganic Syntheses: Reagents for Transition Metal Complex and Organometallic Syntheses* **1990**, *28*, 126-132.

[4] C. F. Lane, G. W. Kabalka, *Tetrahedron* **1976**, *32*, 981-990.

[5] S. Stoll, A. Schweiger, *J. Magn. Res.* **2006**, *178*, 42-55.

[6] G. Sheldrick, *Acta Cryst. A* **2015**, *71*, 3-8.

[7] G. Sheldrick, *Acta Cryst. A* **2008**, *64*, 112-122.

[8] A. L. Spek, *Acta Cryst.* **2009**, *D65*, 148-155.

[9] A. D. Becke, *J. Chem. Phys.* **1993**, *98*, 5648–5652.

[10] C. Lee, W. Yang, R. G. Parr, *Phys. Rev. B* **1988**, *37*, 785–789.

[11] S. H. Vosko, L. Wilk, M. Nusair, *Can. J. Phys.* **1980**, *58*, 1200–1211.

[12] P. J. Stephens, F. J. Devlin, C. F. Chabalowski, M. J. Frisch, *J. Phys. Chem.* **1994**, *98*, 11623–11627.

[13] T. Yanai, D. P. Tew, N. C. Handy, *Chem. Phys. Lett.* **2004**, *393*, 51–57.

[14] J.-D. Chai, M. Head-Gordon, *Phys. Chem. Chem. Phys.* **2008**, *10*, 6615.

[15] R. Krishnan, J. S. Binkley, R. Seeger, J. A. Pople, *J. Chem. Phys.* **1980**, *72*, 650–654.

[16] R. D. Johnson III, "NIST Computational Chemistry Comparison and Benchmark Database.," **2013**.

[17] R. Seeger, J. A. Pople, *J. Chem. Phys.* **1977**, *66*, 3045–3050.

[18] R. Bauernschmitt, R. Ahlrichs, *J. Chem. Phys.* **1996**, *104*, 9047–9052.

[19] R. Bauernschmitt, R. Ahlrichs, *Chem. Phys. Lett.* **1996**, *256*, 454–464.

[20] R. E. Stratmann, G. E. Scuseria, M. J. Frisch, *J. Chem. Phys.* **1998**, *109*, 8218–8224.

[21] F. Furche, R. Ahlrichs, *J. Chem. Phys.* **2002**, *117*, 7433–7447.

[22] C. Adamo, D. Jacquemin, *Chem. Soc. Rev.* **2013**, *42*, 845–856.

[23] A. V. Marenich, C. J. Cramer, D. G. Truhlar, *J. Phys. Chem. B* **2009**, *113*, 6378–6396.

[24] T. H. Dunning, *J. Chem. Phys.* **1989**, *90*, 1007–1023.

[25] D. Figgen, K. A. Peterson, M. Dolg, H. Stoll, *J. Chem. Phys.* **2009**, *130*, 164108.

[26] M. P. Mitoraj, A. Michalak, T. Ziegler, *J. Chem. Theory Comput.* **2009**, *5*, 962–975.

[27] K. Morokuma, *J. Chem. Phys.* **1971**, *55*, 1236–1244.

[28] T. Ziegler, A. Rauk, *Theor. Chim. Acta* **1977**, *46*, 1–10.

[29] M. von Hopffgarten, G. Frenking, *Wiley Interdiscip. Rev. Comput. Mol. Sci.* **2012**, *2*, 43–62.

[30] M. P. Mitoraj, A. Michalak, T. Ziegler, *Organometallics* **2009**, *28*, 3727–3733.

[31] a) E. van Lenthe, E. J. Baerends, J. G. Snijders, *J. Chem. Phys.* **1993**, *99*, 4597–4610; b) E. van Lenthe, E. J. Baerends, J. G. Snijders, *J. Chem. Phys.* **1994**, *101*, 9783–9792; c) E. van Lenthe, A. Ehlers, E.-J. Baerends, *J. Chem. Phys.* **1999**, *110*, 8943–8953.

[32] I. Mayer, *Chem. Phys. Lett.* **1983**, *97*, 270–274.

[33] I. Mayer, *Int. J. Quantum Chem.* **1984**, *26*, 151–154.

[34] K. B. Wiberg, *Tetrahedron* **1968**, *24*, 1083–1096.

[35] A. J. Bridgeman, G. Cavigliasso, L. R. Ireland, J. Rothery, *J. Chem. Soc. Dalt. Trans.* **2001**, 2095–2108.

[36] a) F. Weigend, R. Ahlrichs, *Phys. Chem. Chem. Phys*. **2005**, *7*, 3297; b) Y. Zhao, D. G. Truhlar, *Theor. Chem. Acc*. **2008**, *120*, 215–241; c) A. V. Marenich, C. J. Cramer, D. G. Truhlar, *J. Phys. Chem. B* **2009**, *113*, 6378–6396; d) S. Grimme, J. Antony, S. Ehrlich, H. Krieg, *J. Chem. Phys.* **2010**, *132*, 154104; e) S. Grimme, S. Ehrlich, L. Goerigk, *J. Comput. Chem*. **2011**, *32*, 1456–1465.

[37] H. Braunschweig, K. Radacki, R. Shang, C. W. Tate, *Angew. Chemie Int. Ed*. **2013**, *52*, 729–733

[38] C. P. Kelly, C. J. Cramer, D. G. Truhlar, *J. Chem. Theory Comput.* **2005**, *1*, 1133–1152.

[39] a) K. Fukui, *Acc. Chem. Res.* **1981**, *14*, 363–368; b) H. P. Hratchian, H. B. Schlegel, in *Theory and Applications of Computational Chemistry* (Eds.: C.E. Dykstra, G. Frenking, K.S. Kim, G. Scuseria), Elsevier, Amsterdam, **2005**, 195–249.

[40] a) A. Klamt, G. Schüürmann, *J. Chem. Soc., Perkin Trans. 2* **1993**, 799–805; b) A. Klamt, *J. Phys. Chem.* **1995**, *99*, 2224–2235; c) A. Klamt, V. Jonas, *J. Chem. Phys.* **1996**, *105*, 9972–9981.

[41] M. J. Frisch, G. W. Trucks, H. B. Schlegel, G. E. Scuseria, M. A. Robb, J. R. Cheeseman, G. Scalmani, V. Barone, B. Mennucci, G. A. Petersson, H. Nakatsuji, M. Caricato, X. Li, H. P. Hratchian, A. F. Izmaylov, J. Bloino, G. Zheng, J. L. Sonnenberg, M. Hada, M. Ehara, K. Toyota, R. Fukuda, J. Hasegawa, M. Ishida, T. Nakajima, Y. Honda, O. Kitao, H. Nakai, T. Vreven, J. A. Montgomery, Jr., J. E. Peralta, F. Ogliaro, M. Bearpark, J. J. Heyd, E. Brothers, K. N. Kudin, V. N. Staroverov, R. Kobayashi, J. Normand, K. Raghavachari, A. Rendell, J. C. Burant, S. S. Iyengar, J. Tomasi, M. Cossi, N. Rega, J. M. Millam, M. Klene, J. E. Knox, J. B. Cross, V. Bakken, C. Adamo, J. Jaramillo, R. Gomperts, R. E. Stratmann, O. Yazyev, A. J. Austin, R. Cammi, C. Pomelli, J. W.

Ochterski, R. L. Martin, K. Morokuma, V. G. Zakrzewski, G. A. Voth, P. Salvador, J. J. Dannenberg, S. Dapprich, A. D. Daniels, O. Farkas, J. B. Foresman, J. V. Ortiz, J. Cioslowski, D. J. Fox, *Gaussian 16, Revision B.01*; Gaussian, Inc.: Wallingford CT, **2016**.

[42] G. te Velde, F. M. Bickelhaupt, E. J. Baerends, C. Fonseca Guerra, S. J. A. van Gisbergen, J. G. Snijders, T. Ziegler, *J. Comput. Chem.* **2001**, *22*, 931–967.
